# Evaluating the Effect of Irradiation on the Densities of Two RNA Viruses in *Glossina morsitans morsitans*

**DOI:** 10.3390/insects14040397

**Published:** 2023-04-20

**Authors:** Caroline K. Mirieri, Adly M.M. Abd-Alla, Vera I.D. Ros, Monique M. van Oers

**Affiliations:** 1 Laboratory of Virology, Wageningen University and Research, Droevendaalsesteeg 1, 6708 PB Wageningen, The Netherlands; 2 Insect Pest Control Laboratory, Joint FAO/IAEA Programme of Nuclear Techniques in Food and Agriculture, International Atomic Energy Agency, Vienna International Centre, P.O. Box 100, 1400 Vienna, Austria; a.m.m.abd-alla@iaea.org

**Keywords:** *Glossina*, tsetse, iflavirus, negevirus, sterile insect technique, irradiation

## Abstract

**Simple Summary:**

Tsetse flies transmit Trypanosoma parasites, which cause severe diseases in humans and animals. To reduce the number of tsetse flies, male flies are sterilized through irradiation and released into the field to compete with wild male flies for mating with wild females. Recently, two insect-specific viruses were discovered in mass-reared *Glossina morsitans morsitans* tsetse flies. The aim of this study was to evaluate whether these viruses are affected by the irradiation treatment. The results showed that irradiation did not change the number of viral genome copies in the flies.

**Abstract:**

Tsetse flies are cyclic vectors of Trypanosoma parasites, which cause debilitating diseases in humans and animals. To decrease the disease burden, the number of flies is reduced using the sterile insect technique (SIT), where male flies are sterilized through irradiation and released into the field. This procedure requires the mass rearing of high-quality male flies able to compete with wild male flies for mating with wild females. Recently, two RNA viruses, an iflavirus and a negevirus, were discovered in mass-reared *Glossina morsitans morsitans* and named GmmIV and GmmNegeV, respectively. The aim of this study was to evaluate whether the densities of these viruses in tsetse flies are affected by the irradiation treatment. Therefore, we exposed tsetse pupae to various doses (0–150 Gy) of ionizing radiation, either in air (normoxia) or without air (hypoxia), for which oxygen was displaced by nitrogen. Pupae and/or emerging flies were collected immediately afterwards, and at three days post irradiation, virus densities were quantified through RT-qPCR. Generally, the results show that irradiation exposure had no significant impact on the densities of GmmIV and GmmNegeV, suggesting that the viruses are relatively radiation-resistant, even at higher doses. However, sampling over a longer period after irradiation would be needed to verify that densities of these insect viruses are not changed by the sterilisation treatment.

## 1. Introduction

For many decades, tsetse flies and trypanosomiasis have been a scourge on the African continent, exacerbating the disease burden in humans and livestock, and enlarging economic constraints that already come from other sources [1,2,3,4]. Tsetse flies (*Glossina* spp.) are prevalent in several countries in sub-Saharan Africa [5,6], of which the *Glossina morsitans morsitans* (Gmm) species is mainly found in Zimbabwe [7]. These flies transmit protozoans of the Trypanosoma spp., the causative agents of trypanosomiasis, diseases of humans (human African trypanosomiasis) [8] and livestock (African animal trypanosomiasis) [9].

Tsetse flies have been a focus of many control tactics aimed at suppressing and eradicating both the flies and the transmitted diseases [10,11]. Vector control methods such as bush clearing and the use of residual chemicals were used early on [12], but in recent decades, a transition to more environmentally friendly tactics has been witnessed, along with the use of an area-wide integrated pest management (AW-IPM) approach that targets entire vector populations [13,14]. These control methods rely on the utilization of several tactics to suppress vector populations, including trapping with live baits, and the use of stationery targets and non-residual chemicals, culminating in the use of the sterile insect technique (SIT) for the eradication of these vectors [13,15,16]. SIT has previously been successfully used for the eradication of tsetse flies in Zanzibar [17], and as a result, several African countries have endeavoured to use the same technique to also eradicate tsetse flies from their own area [18,19,20]. SIT relies on the successful mass rearing and release of high-quality male flies that have been sterilized through exposure to ionizing radiation to compete with wild males for mating with wild females [21]. Therefore, attention must be paid to the health of mass-reared tsetse flies to ensure high performance during release in order to achieve the aim of an SIT program in a target area [21,22]. Viral infections may challenge the health and performance of mass-reared insect colonies, as reported for various mass-reared insects including honeybees, silkworms, fruit flies, tsetse flies, soldier flies and crickets [23,24,25,26,27,28], and may cause enormous economic losses [29,30,31,32]. These viral infections may be present in a covert (asymptomatic or sub-lethal) state, either as latent (non-productive) or persistent (involving a low-level production of virus progeny) infections. The viral infections may rely on vertical and/or horizontal transmission [28,33], and the activation of latent or persistent viruses may lead to symptomatic or even lethal disease when the insect host is under stress, compromising the immune system [33,34,35].

For mass-reared tsetse flies, the salivary gland hypertrophy virus (SGHV) was the first virus to be reported in either a covert or an overt state [32]. Overt infections have been majorly reported in *Glossina pallidipes*, unlike other *Glossina* spp. where the virus has been reported as a covert infection [31,36]. Infections with SGHV in mass-reared tsetse flies resulted in several negative consequences including reduced fly fertility and fecundity, and even colony collapse [31,37,38,39,40]. Mass-reared *G. pallidipes* succumbed to SGHV, which manifested by hypertrophied salivary glands, sterility in males and reduced fecundity and fertility in females [24]. SGHV infections were also found to reduce pupal weight and to prolong the first and second pregnancy cycles in *G. pallidipes* [41]. Furthermore, in a different study, infected males were of reduced size and could not form a complete spermatophore [42]. SGHV was the only reported virus in *Glossina* spp. until a recent study in which infections with Glossina morsitans morsitans iflavirus (GmmIV) and Glossina morsitans morsitans negevirus (GmmNegeV) were discovered and characterised in the tsetse species *Glossina morsitans morsitans* (Gmm) [43]. Iflaviruses (family *Iflaviridae*) and negeviruses (family *Negeviridae*) are insect-specific viruses, with members of the family *Negeviridae* so far restricted to dipteran insects, including mosquitoes, phelobotomine sandflies and dung flies from a wide range of geographical areas [44,45,46]. In contrast, iflaviruses have a wide host range within arthropods, having been detected in lepidopterans, hymenopterans (including bees), hemipterans and acarinans (bee parasitic mites) [44]. Generally, iflavirus particles are icosahedral, non-enveloped and usually about 30 nm in diameter, with a polyadenylated positive-sense single strand RNA genome ranging from 8.8 to 10.3 kb [44,47,48,49]. Negeviruses have spherical particles of 45–55 nm in diameter and have polyadenylated positive-sense single strand RNA genomes ranging between 9 and 10 kb [45,46,50]. The newly characterized GmmIV encodes a single polyprotein, which is post-translationally processed into structural and non-structural proteins [43], while the GmmNegeV genome contains two overlapping ORFs [43]. In situ hybridization experiments demonstrated that GmmIV and GmmNegeV RNA can be found in the brain, fat bodies, entire gut, malpighian tubules, milk glands, ovaries and salivary glands of the infected flies [43]. However, the impact of these two recently discovered viruses on Gmm in mass rearing facilities has not been elucidated, leaving it to speculation as to whether they are beneficial or detrimental to the immunity, fitness and performance of the fly. How these viruses are affected by SIT irradiation treatments is also unknown.

The sterilization of the tsetse males uses ionizing radiation, which causes damage to the chromosomes of gonial cells (specifically, germ cell chromosome fragmentation), including dominant lethal mutations, translocations and other aberrations. Consequently, imbalanced gametes are produced, leading to the inhibition of mitosis and death of the fertilized eggs or embryos, resulting in a lack of progeny from the sterile males mating with wild females [51,52]. Ionizing radiation and conditions during irradiation (presence or absence of oxygen) may bring about the needed sterilization, but may also cause damage to the biological quality of the tsetse flies, affecting their emergence, flight, mating competitiveness and survival, among other qualities. Additionally, irradiation may affect any associated microbial communities, including viruses, which may affect the competitiveness of the flies after release [53,54,55,56]. The current irradiation procedure used for tsetse flies in many mass-rearing facilities is conducted with ionizing radiation at the pupal stage, just after the female pupae have emerged or are separated. The irradiation is usually carried out in air (oxygen), but studies have reported benefits to the performance of fruit flies when pupae were irradiated in hypoxia (low-oxygen conditions) [57,58]. Irradiation in air conditions (as conducted currently for tsetse flies) is hypothesized to cause oxidative stress [59], enhancing the negative effect of damaging free radicals produced during the normal metabolism of cells and tissues of the insect, thus explaining the protective effect of hypoxia found in fruit flies [60,61,62]. While there is still a concern about the impact of GmmIV and GmmNegeV infections on the mass rearing of tsetse flies (mainly Gmm), it is also imperative to know how irradiation affects the densities of these viruses, as this may eventually affect the performance of tsetse flies for SIT [54,55]. Therefore, we aimed to determine whether GmmIV and GmmNegeV densities in Gmm pupae and flies were affected by irradiation in the pupal stage either under normoxic or hypoxic conditions.

## 2. Materials and Methods

### 2.1. Tsetse Flies

The pupae used for the experiments were derived from the Gmm colony originating from Zimbabwe and maintained at the Insect Pest Control Laboratory (IPCL), FAO/IAEA Joint Center of Nuclear Techniques in Food and Agriculture, Seibersdorf, Austria. The colony was maintained using an in vitro membrane feeding system with thawed and heated bovine blood (Svaman spol, s.r.o., Myjava, Slovak Republic). The blood was kept frozen at −20 °C and irradiated with 1 kGy in a commercial 220 PBq Cobalt-60 (^60^Co) wet storage panoramic shuffle irradiator. The flies were offered a warm blood meal on a sterile membrane three times a week, and were maintained under a 12L:12D light cycle. Pupae were incubated at 24.1 ± 0.1 °C and 78.8 ± 3.7% R.H., and adults emerged at the same temperature and humidity and were collected using the self-stocking production cage system [63]. The above conditions will, henceforth, be referred to as standard laboratory rearing conditions.

### 2.2. Irradiation Conditions

The Gmm pupae were irradiated at the IPCL, Seibersdorf, Austria, using a Gammacell^®^ 220 (MDS Nordion Ltd., Ottawa, ON, Canada) ^60^Co irradiator. The dose rate was measured through alanine dosimetry as 2.144 Gy·sec−1 on 3 March 2015 with an expanded uncertainty (k = 2) of 1.6%. The radiation field was mapped using Gafchromic HD-V2 film, and the dose uniformity ratio in the volume used for the experiments was <1.1. One at a time, the irradiated groups were removed from the box containing phase change packs (Climsel C7, Climator Sweden AB, Mejselvägen 15, SE-541 34 Skövde, Sweden), which kept the temperature below 10 °C, and each bottle was placed at the bottom of a polycarbonate jar (2200 mL) during irradiation at the various doses previously mentioned.

### 2.3. The Irradiation Process and Collection of Samples

A one-day collection of mainly male pupae that were ready to emerge (between 29 and 30 days old) was divided into 11 groups of 80–100 pupae. The first control group (no chilling, no irradiation and no hypoxia), labelled as ‘no treatment’, was immediately placed in an emergence cage, while the other groups were maintained under chilling at 10 °C until they were placed in emergence cages after the irradiation process. To prepare for irradiation, these pupae were placed in 250 mL plastic bottles (Corning^®^ PET Media Bottles with Leak-proof Screw Cap, Corning, New-York, NY, USA) (Supplementary Figure S1) for the purpose of establishing hypoxic conditions by displacing oxygen with nitrogen. At one end of each bottle, an inlet was drilled on the lower side, and at the opposite end, an outlet was drilled on the upper side and fitted with a three-way stopcock, with a rotating male luer lock–female connector (Grayline Medical, Norwalk, CA, USA) to control the flushing of nitrogen through the bottle. On the upper side of the inlet, an extra hole was drilled and covered with a Styrofoam septa disc to allow piercing through for the measuring of oxygen levels during hypoxia using a gas analyser needle (Supplementary Figure S1a–c). Hypoxic conditions were achieved by capping the bottles and flushing nitrogen through the inlet, allowing for the displaced oxygen to be flushed out through the outlet for at least 1 min. The outlets, followed by the inlets, were closed, and the sealing in of air was reinforced by wrapping the bottles with cling paper, before the oxygen levels (and also the CO_2_ levels as an indicator of metabolism) were measured using a gas analyser (Dansensor^®^ CheckMate 3, Mocon Inc., Brooklyn Park, MN, USA) (Supplementary Figure S1d). The other set of bottles to be used for irradiation in normoxia were prepared by cutting out large rectangular openings on all sides of each bottle, which were covered with a netting material to ensure sufficient circulation of oxygen.

A total of three bottles of pupae were irradiated in normoxia at the doses of 70, 110 or 150 Gy, and an additional bottle served as control group of ‘chilling only (0 Gy)’ and was not irradiated, but was held under chilling through the whole nitrogen flushing and irradiation period of the other groups. Three other bottles of pupae were irradiated in hypoxia at the doses of 110, 150, or 190 Gy, and a fourth bottle served as control group of ‘hypoxia only (0 Gy)’, which was held under hypoxia and was chilled as explained for the normoxia control group. The experiment was conducted in 3 biological replicates for the doses of 70, 110 and 150 Gy in normoxia, and for the doses of 110 and 150 Gy in hypoxia. However, there were 2 only biological replicates for the 70 Gy, and one for the 190 Gy in hypoxia. For one replicate, the dose of 70 Gy was replaced by 190 Gy to have an idea of what would happen at higher doses under hypoxia conditions, given that higher doses are required to achieve the same levels of sterility in hypoxia, as seen at lower doses when irradiation is performed in normoxia. 

After one hour in hypoxia, the pupae from both the normoxia and hypoxia were irradiated in the irradiator under the conditions previously mentioned, and immediately afterwards, the level of O_2_ was measured (<5%) again. The hypoxia was broken and samples of 4 pupae from each treatment (and replicate) used in this study were collected in individual Eppendorf tubes containing TRIzol^TM^ reagent (Thermo Fischer Scientific, Waltham, MA, USA) (1 pupae in each tube) and preserved in a −80 °C freezer for the extraction of RNA. The remaining pupae from each treatment were then placed in separate emergence cages for 3 days to allow for the emergence, and samples of 4 adult flies (from each treatment and replicate) were also collected in Eppendorf tubes (1 fly in each tube) containing TRIzol^TM^ and preserved in a −80 °C freezer for RNA extraction at the IAEA laboratory.

### 2.4. RNA Extraction and DNase Treatment

The RNA extraction process was conducted according to the manufacturer’s instructions (Invitrogen user guide for TRIzol™ Reagent, Thermo Fisher Scientific, Waltham, MA, USA) with slight modifications. All the reagents used in the extraction process were halved since the pupae and adult flies were less than 50 mg (the recommended tissue weight for the protocol as it is). A total of 4 pupae or 4 adult flies from each treatment for each replicate were individually transferred from the RNAlater solution (Thermo Fisher Scientific, Waltham, MA, USA) into an empty Eppendorf tube, to which 0.5 mL of cold TRIzol^TM^ (4 °C) was added. Briefly, each pupa or adult fly was crushed using a plastic pestle inside the Eppendorf tube with 0.5 mL of TRIzol^TM^, and due to the high content of fat and other debris from the whole fly, the lysate was centrifuged for 5 min at 12,000× *g* at 4–10 °C, and the clear supernatant was transferred to a new Eppendorf tube. The clear supernatant was incubated for 5 min to allow the complete dissociation of the nucleoprotein complexes, after which 0.1 mL of chloroform was added to the tube, which was then incubated for 2–3 min with shaking of the tube for proper mixing of the supernatant and the chloroform. The sample was centrifuged again for 15 min at 12,000× *g* at 4–10 °C, causing the mixture to separate into three layers, the uppermost of which was a colourless aqueous phase containing the RNA. The aqueous phase was transferred to a new tube by pipetting the solution from the tube by tilting the tube at an angle of 45°. A total of 0.25 mL of isopropanol was then added to this aqueous phase, which was then incubated for 10 min, after which the sample was centrifuged at 12,000× *g* at 4–10 °C for another 10 min. The supernatant was discarded and the resulting white gel-like pellet at the bottom of the tube was resuspended in 0.5 mL of 75% ethanol. The sample was vortexed briefly and centrifuged for 5 min at 7500× *g* at 4 °C, and the supernatant was also discarded. The sample was left to dry on a clean surface for approximately 10–15 min. The pellet was then resuspended in autoclaved milliQ water (RNase-free water) preheated on a heat block at 55 °C, and incubated in a water bath at the same temperature for approximately 15 min. The quality and concentration (RNA yield) of the sample was measured with a NanoDrop-1000 spectrometer (Thermo Fisher Scientific, Waltham, MA, USA), obtaining an absorbance ratio at 260 nm and 280 nm. This process was repeated concurrently for all the samples within each biological replicate. The samples were stored at −80 °C for downstream applications.

Due to economic reasons, the viral densities were measured in pooled RNA from 4 pupae/flies of each treatment in 3 replicates. Dilutions of the samples were made in new tubes to standardize the RNA concentrations to 80 ng/µL by adding milliQ water, in order to calculate volumes and concentrations of the total RNA according to the original concentration of each fly sample. This was followed by DNase treatment of the sample following the manufacturer’s instructions (Invitrogen, DNA-free TM Kit, Waltham, MA, USA) to remove any host DNA from the sample. Briefly, 10 µL of the standardized total RNA concentrations of each fly from the same treatment was pooled together in a new tube, making a total volume of 40 µL, and treated with a 4 µL unit of DNase 1 buffer and 1 µL of DNase in a total volume of 45 µL, following the supplier instructions. The tube was gently flicked to mix the contents, and the sample was incubated at 37 °C for 30 min. After incubation, 4.5 µL of DNase Inactivation Reagent was added to the tube containing the RNA, and the tubes were incubated again for 2 min at room temperature while flicking to redisperse the DNase Inactivation Reagent. The tubes were centrifuged at 10,000× *g* for 1.5 min at room temperature, after which at least 15–20 µL of the supernatant was transferred to a new tube. The tubes were stored at −80 °C until cDNA synthesis.

### 2.5. cDNA Synthesis

cDNA was synthesized using the SuperScript^®^ III First-Strand Synthesis System for RT-PCR (Invitrogen, Waltham, MA, USA) from the DNA-free total RNA that had been stored in the −80 °C freezer, according to the manufacturer’s instructions. The total amount of 1 µL oligo(dT) and 1 µL 10 mM dNTP needed for each of the samples was mixed in one tube, and thereafter, 2 µL was distributed for each reaction (each well) in a 96-well PCR plate. A total amount of 8 µL of the total RNA of each sample was also added to each well, and the plate was centrifuged before placement in the PCR machine for incubation at 65 °C for 5 min, then placed on ice for 1 min. In another tube, 2 µL 10× RT buffer, 4 µL 25 mM MgCL_2_, 2 µL 0.1 M DTT, 0.5 µL RNase OUT™ (40 U/µL) and 1 µL superscript III (200 U/µL) were added in that order and mixed for each reaction. This mixture (10 µL) was added to each sample in the plate, bringing the final volume in each well to 20 µL. The plate containing the samples, together with the negative and positive controls, was placed in the PCR machine at 50 °C for 50 min and 85 °C for 5 min before being chilled on ice and being used for the next step of qPCR.

### 2.6. Relative Quantitative PCR

To compare the relative copy number of the viral RNA virus to the housekeeping gene (β-tubulin) expression levels (hereafter referred to as “density”) after each treatment, a relative quantitative PCR (qPCR) was performed. The reagents, including the SYBR Green qPCR master mix (two-fold concentrated) (Biorad, Hercules, CA, USA) and the forward and reverse primers (sequence and primer positions shown in Table 1) at 10 pM concentrations, were mixed with either autoclaved milliQ water (for negative control reaction) or with water and cDNA for test analysis in a clean sterile tube at volumes of 7.5 µL, 0.5 µL, 0.5 µL and 4.5 µL, respectively, totalling a volume of 15 µL for each reaction.

Three independent reactions were used for each sample for the tsetse fly housekeeping gene (β-tubulin), the GmmIV and the GmmNegeV as they each had their own primers. The mixture for each reaction was distributed in a 96-well white BR qPCR plate (brand) where half the plate contained β-tubulin and the other half either GmmIV or GmmNegeV at a time. A total volume of 2 µL of each cDNA sample was added to each well in duplicates for tubulin and duplicates for GmmIV/GmmNegeV. Only 22 samples were loaded each time, with the 23rd and 24th as the positive (tsetse samples proven infected with GmmIV or GmmNegeV) and negative controls for the qPCR reaction. The samples were centrifuged and placed in a CFX96 Real-Time System cycler (Bio-Rad, Hercules, CA, USA) for the quantification of the virus densities according to the irradiation treatments. The machine was set at a default threshold and a melting temperature of 62 °C, and the plate was set according to the target genes and fluorophore (SYBR Green). The efficiency and the melting peaks of the primers had been previously determined. The normalization of the viral density was calculated in CFX-Maestro V.2.2 software by dividing the viral relative florescent units by those of tubulin obtained for the same sample. The resulting data were also normalized against the controls (0 Gy chilling only and 0 Gy hypoxia only) before using them for statistical analysis.

### 2.7. Statistical Analysis

The data were organised in Excel sheets and saved in csv format for analysis using R software. The following packages were used in the analysis: datasets, ggplot2 [64] and lme4 [65] for fitting and analyzing mixed models; nlme [66] for linear and nonlinear mixed effects models; MuMin [67] for model selection; plyr [68] and dplyr [69] for data manipulation; ranger [70] and MASS for data transformation [71]; tidyverse [72] for science data structure and knitr [73] and R Markdown to produce a report of the analysis [74]. The raw data from the real-time thermocycler (CFX Biorad) were normalized against the housekeeping gene, after which they were normalized against the control treatments (0 Gy chilling only and 0 Gy hypoxia only) before proceeding with the analysis. The data were checked for normality using the Box–Cox method, which generated a lambda value that was used to transform the data as they were not normally distributed. A histogram, qqnorm and qqline plots were used to visualize the data before and after transformation to confirm the transformation. The differences among and between groups (species, irradiation doses, irradiation conditions (air status) and life stages) were determined using ANOVA. Generalized linear mixed models (GLMM) fit by maximum likelihood (Laplace approximation) [65] and linear models were used to model the best effects of the individual and combined treatments. The figures were generated using the Tidyverse package [72] in R software. Heat maps were also used to visualize the virus densities under a 2-dimensional influence of either irradiation doses, conditions or the days post irradiation (coinciding with life stages and age of the flies).

## 3. Results

### 3.1. Statistical Distribution of the Data

Box–Cox analysis of the iflavirus and negevirus density data revealed that the data were not normally distributed and, therefore, the data were transformed with a lambda value = −0.1 following the formula “Transformed = (Normalized_expression ^ lambda-1)/lambda”. Consequently, a linear model and ANOVA analysis were used (as detailed in Supplementary File S1).

### 3.2. Impact of Irradiation Treatment on GmmIV Densities

**Figure 1:**
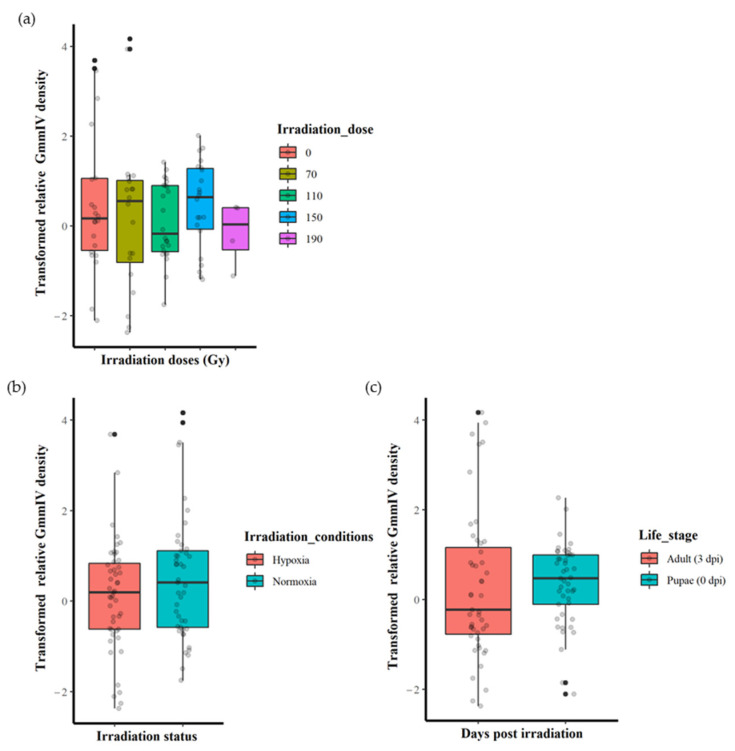
Effect of irradiation doses, irradiation conditions and host life stage on the GmmIV densities in *G. morsitans morsitans*. (**a**) GmmIV densities as affected by irradiation dose, regardless of irradiation conditions (normoxia or hypoxia) and host life stages. (**b**) GmmIV densities as influenced by irradiation conditions (irradiation in normoxia or hypoxia), regardless of irradiation dose and life stages. (**c**) GmmIV densities as affected by life stages, regardless of irradiation dose and irradiation conditions (irradiation normoxia or hypoxia). There were no significant differences observed (*p* > 0.05). The dots refer to individual measurements, black dots indicate a second measurement with equal value.

The irradiation dose (0, 70, 110, 150 or 190 Gy) did not significantly affect the GmmIV density (F = 0.5443, df = 4, 87, *p* = 0.7036) when the variation in irradiation conditions and life stages were not taken into account (Figure 1a and Supplementary File S1). There were also no significant differences in virus densities (F = 2.2361, df = 1, 90, *p* = 0.1383) between irradiation in normoxia or hypoxia when differences in irradiation doses and life stages were disregarded (Figure 1b and Supplementary File S1). Furthermore, there were no significant differences in the GmmIV density in teneral adults and pupae (F = 0.0759, df = 1, 90, *p* = 0.7836) when differences in irradiation doses and conditions were disregarded (Figure 1c and Supplementary File S1). The linear models (mod 8) showed that irradiation conditions (as a single factor) were the major influence, although not significant (t = 1.495, *p* = 0.138), on the variations in GmmIV densities observed in the pupae/flies (Supplementary File S1). To investigate the impact of additive or interactive effects between irradiation doses, irradiation conditions and host life stages, different linear models were tested, and the AICc values indicated that analysing each factor separately produced the lowest AICc value, with irradiation conditions (mod8 AICc = 315.2693) followed by host life stage (mod9 AICc = 317.4496) and irradiation doses (mod7 AICc = 321.9685). All interaction models produced higher AICc values, indicating lower-fit models (Supplementary File S1).

### 3.3. Impact of Irradiation Conditions and Host Stage on GmmIV Densities

**Figure 2:**
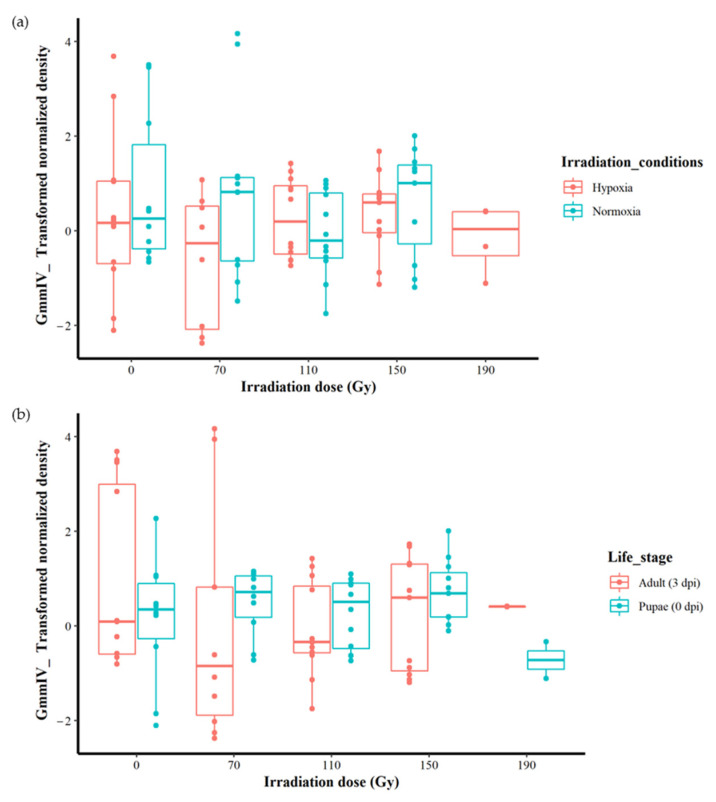
Effect of irradiation conditions and host life stage on the GmmIV densities after exposure to different irradiation doses in *G. morsitans morsitans*. (**a**) Effect of irradiation conditions regardless host life stage. (**b**) Effect of host life stage regardless irradiation conditions.

Although no significant differences were found between different irradiation doses regardless of the irradiation conditions or host stages (Figure 2a,b), more detailed analysis was conducted to explore any hidden effects. As the irradiation conditions produced the best-fit model with the lowest AICc value, we analysed the impact of irradiation doses and host life stages under both normoxia and hypoxia separately. In normoxic conditions, the results indicate that there were no differences in iflavirus density between the irradiation doses (0, 70, 110 and 150 Gy) (F = 1.0729, df = 3, 41, *p* = 0.3712). Similarly, there were also no differences in GmmIV density between host life stages (F = 0.0567, df = 1, 43, *p* = 0.8129) (Supplementary Figure S2a, Supplementary File S1). Testing different linear models indicated that host life stages and irradiation doses produced the lowest AICc values, 163.7558 and 165.3674, respectively, indicating that the host life stages produced the best-fit model compared to irradiation.

Under irradiation in hypoxia, although the irradiation doses and the host life stage had no significant effect on the virus densities when considered as independent factors (F = 1.033, df = 4, 42, *p* = 0.4015 for the irradiation doses and F = 1.0115, df = 1, 45, *p* = 0.883 for the host life stages), there was a significant effect (F = 3.3110, df = 4, 37, *p* < 0.05) on the iflavirus densities with the interactive effects of irradiation and life stages (Supplementary Figure S2b and Supplementary File S1). However, the linear models test indicated that the model including the interaction between the irradiation dose and host life stages is not the best-fit model, with a high AICc value (161.5522) compared with the models including the life stage (AICc = 157.3715) and irradiation doses (AICc = 160.5258) (Supplementary File S1).

### 3.4. Impact of Irradiation Treatment Doses on GmmNegeV Densities

**Figure 3:**
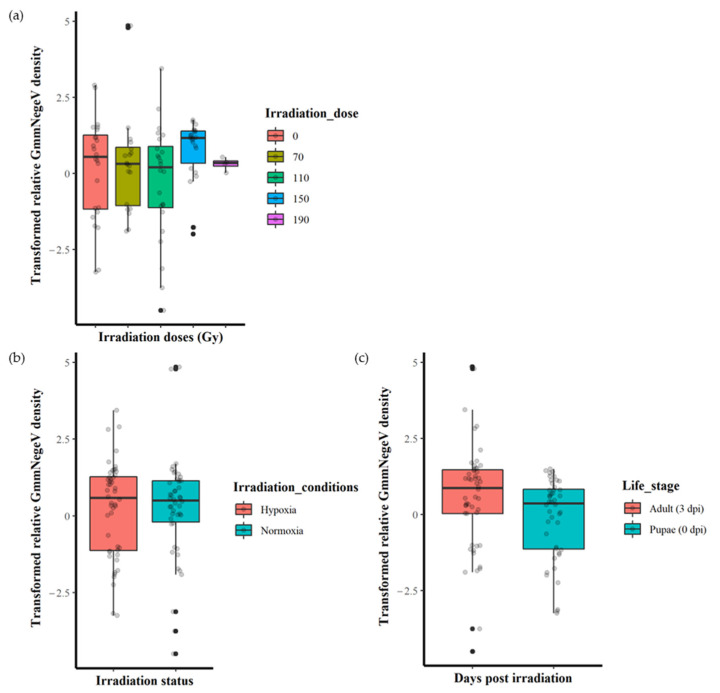
Effect of irradiation doses, irradiation conditions and host stage on the GmmNegeV densities in *G. morsitans morsitans*. (**a**) GmmNegeV densities as affected by irradiation dose, regardless of irradiation conditions (normoxia or hypoxia) and life stages. (**b**) GmmNegeV densities as influenced by irradiation conditions (irradiation in normoxia or hypoxia), regardless of irradiation dose and life stages. (**c**) GmmNegeV densities as affected by life stages, regardless of irradiation dose and irradiation conditions (irradiation normoxia or hypoxia). A significant difference was observed between the life stages (**c**), with higher densities observed at 3 d post irradiation (dpi) (adults) (F = 4.318, df = 1, 92, *p* < 0.05). The dots refer to individual measurements, black dots indicate a second measurement with equal value.

The irradiation doses (0, 70, 110, 150 and 190 Gy) had no significant effect (F = 1.237, df = 4, 89, *p* = 0.301) on the GmmNegeV density (Figure 3a). Additionally, there were no significant differences (F = 0.003, df = 1, 92, *p* = 0.953) in GmmNegeV density when irradiation was performed in normoxia or hypoxia (Figure 3b). However, there were significant differences (F = 4.318, df = 1, 92, *p* < 0.05) in GmmNegeV density as influenced by life stages, with higher densities in the adults (3 dpi) (Figure 3c). 

### 3.5. Impact of Irradiation Conditions and Host Life Stages on GmmNegeV Densities

**Figure 4:**
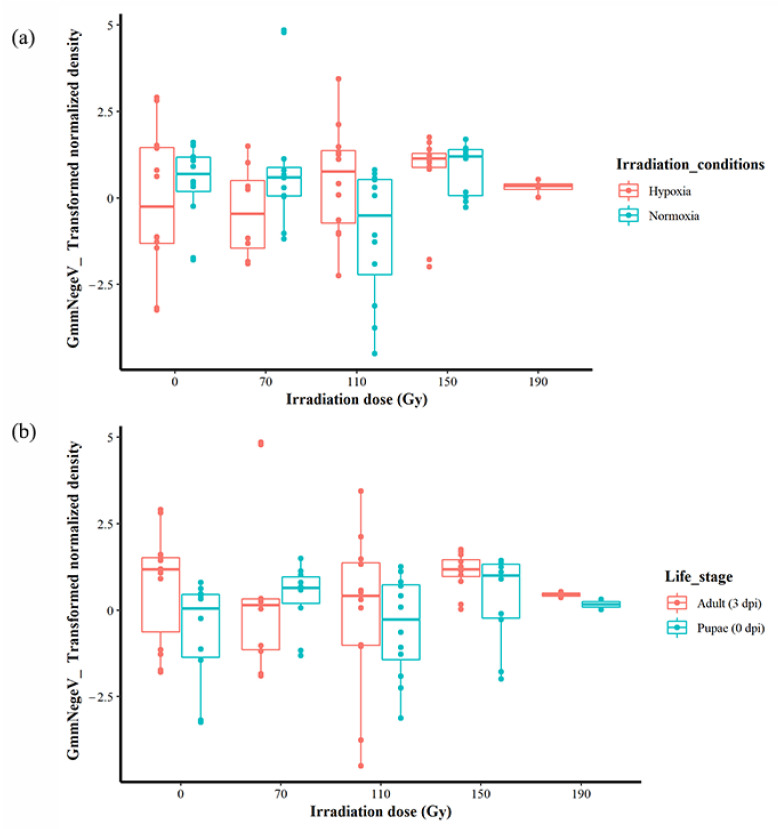
Effect of irradiation conditions and host life stage on the GmmNegeV densities after exposure to different irradiation doses in *G. morsitans morsitans*. (**a**) Effect of irradiation conditions regardless of host life stage. (**b**) Effect of host life stage regardless of irradiation conditions.

When the life stages were not considered, there was no significant effect when irradiation dose and conditions were analysed additively, but there was a significant difference interactively (F = 3.5332, df = 3, 85, 92, *p* < 0.05) (Figure 4a). Additionally, when the irradiation conditions were not taken into account, there was a significant effect of the irradiation dose and the life stages both additively (F = 4.2207, df = 1, 88, *p* < 0.05) and interactively (F = 3.5332, df = 3, 85, *p* < 0.05) (Figure 4b, Supplementary File S1). A linear modelling of all the factors that included interactive and additive effects revealed that the differences in the GmmNegeV density were best explained by life stages/dpi (t = −2.078, *p* < 0.05) (mod9 in Supplementary File S1) in comparison to other factors. The highest density of GmmNegeV was at 0 Gy in hypoxia, followed by 70 Gy (also in hypoxia), in comparison to irradiation in normoxia (Supplementary File S1).

Further analysis to identify hidden effects during irradiation in normoxia revealed significant differences in GmmNegeV density in Gmm, influenced by irradiation doses when analysed as a single factor in addition to and interactively with the fly life stage (F = 4.245, df = 3, 42, *p* < 0.05; F = 4.1086; df = 3, 41, *p* < 0.05; F = 3.8467, df = 3, 38, *p* < 0.05, respectively) (Supplementary Figure S3a and Supplementary File S1). However, there were no differences in GmmNegeV density due to host life stage when considered singly (F = 0.4477, df = 1, 44, *p* = 0.5069), in addition to (F = 0.5426, df = 1, 41, *p* = 0.46556) or in interaction (F = 0.5080, df = 1, 38, *p* = 0.4804) with the irradiation doses (Supplementary Figure S3a and Supplementary File S1). The best model with the lowest AICc values (177.4776) (mod1, Supplementary File S1) revealed that the irradiation doses majorly contributed to the variation in GmmNegeV density observed during irradiation in normoxia, with a significant contribution from the irradiation dose of 110 Gy (t = −2.335, *p* < 0.05), which showed decreased GmmNegeV density compared to the other doses (0, 70 and 150 Gy) in both pupae and adults (Supplementary Figure S3a and Supplementary File S1). However, Tukey’s HSD analysis revealed significant differences between the 110 Gy and 70 Gy dose only (*p* = 0.0126273).

The analysis of the data on irradiation in hypoxia revealed that host life stage of the Gmm flies had a significant impact on the GmmNegeV density, including the additive (F = 5.4991, df = 1, 42, *p* < 0.05) and interactive effects (F = 5.7190, df = 1, 38, *p* < 0.05) with irradiation dose, when its effect was considered as a single factor (F = 5.534, df = 1, 46, *p* < 0.05) (Supplementary Figure S3b and Supplementary File S1). Contrastingly, the irradiation dose did not affect the GmmNegeV density when its effect was considered as a single factor (F = 0.8397, df = 4, 43, *p* = 0.507), in addition to (F = 0.9276, df = 4, 42, *p* = 0.457) or in interaction with host life stages (F = 1.4199, df = 4, 38, *p* = 0.246) (Supplementary Figure S3b and Supplementary File S1). The linear model test of the effect of factors showed that host life stages best explained the variation in GmmNegeV density as observed during irradiation in hypoxia in increasing days after irradiation and increasing negevirus densities in Gmm. The best model (mod11) showed a significant increase in GmmNegeV density at adult stage at 3 dpi (t = 2.352, df = 1, 46, *p* < 0.05) compared to the density in pupae at 0 dpi (Supplementary File S1).

## 4. Discussion

This study was conducted to determine the impact of irradiation on the densities of the newly discovered iflavirus (order *Picornavirales*, family *Iflaviridae* and genus *Iflavirus*) and negevirus (family *Negeviridae* and genus *Negevirus*) in the tsetse species *Glossina morsitans morsitans* [43]. All sample pools of flies (all populations) in this study tested positive for these viruses, although the flies had no obvious signs of infection (hence asymptomatic). The high prevalence of both viruses in Gmm is in agreement with a previous study [43]. The asymptomatic infection implies sublethal replication and the ability of these viruses to silence or evade the immune system, likely through mechanisms such as apoptosis or miRNAs [75,76,77]. Prevalence in the whole population (and possibly all tissues, as our investigations were on the RNA extracted from the whole fly body) concurs with a previous study where both viruses were found in all sample pools of investigated tissues [43]. Additionally, the presence of the GmmIV and GmmNegeV viruses in both pupae and adults suggests the possibility of their transmission from generation to generation, as also found in the aforementioned study, where the viruses were also found in the reproductive organs (ovaries, testes and spermatheca) [43]. Vertical transmission through eggs during covert infections has been demonstrated for the deformed winged virus (DWV) in studies of honey bees (*Apis mellifera*) [78]. However, the horizontal and/or vertical transmission of GmmIV and GmmNegeV is yet to be proven.

Overall, the analysis of our results showed that there were no significant differences in the GmmIV and GmmNegeV densities in irradiated Gmm. Additionally, no differences were found between the densities of the two viruses as influenced by the irradiation doses and the irradiation conditions (normoxia vs. hypoxia), as well as the host life stages, pupae (at 0 dpi) and adults (at 3 dpi). There were no differences in GmmIV densities affected by irradiation doses, irradiation conditions or host life stages. However, during irradiation in hypoxia, the density of GmmIV at 70 Gy was significantly lower than that of the non-irradiated adults. In contrast, there was a significantly higher density of GmmNegeV in adults compared to pupae, but there were no differences in densities for the different irradiation doses and irradiation conditions. Additionally, during irradiation in normoxia, irradiation doses (in addition to and interactively with the fly stage) affected the GmmNegeV density, with the dose of 110 Gy significantly decreasing the density compared to the control, but the life stage had no effect on the density on its own. However, during irradiation in hypoxia, it is the life stages/dpi (in addition to and interactively with the irradiation doses) that had the major effect on the GmmNegeV densities, while the irradiation doses had no effect on their own. Notably, greater differences in GmmNegeV density were observed without irradiation in the control (only hypoxia at 0 Gy), demonstrating that the irradiation dose was not the main influence in the observed difference in density and, therefore, it is highly possible that hypoxia increases virus densities compared to normoxia.

The differences in density as affected by fly life stage were mainly evident between pupae and adults for GmmNegeV, but GmmIV densities remained relatively stable. The increase in GmmNegeV density in life stages may be attributed to the replication of this virus, corroborating with investigations in the previous study [43]. It is also congruent with additional preliminary data from our studies (not published), which indicated that GmmNegeV densities increased with fly age although GmmIV density decreased over time/in adults (unlike our study, where there are no differences in pupae and adults/dpi) in non-irradiated adult flies. Replication of the salivary gland hypertrophy virus (GpSGHV) in tsetse flies over time and life stages has also been shown in a previous study [36]. Similarly, it was observed that after infection with iflaviruses 1 and 2 (SeIV-1 and SeIV-2) in *Spodoptera exigua* (Noctuidae; Lepidoptera), the abundance of SeIV-1 increased (~10,000 fold), whereas that of SeIV-2 remained constant, across insect developmental stages [35], also evidence for differential replication rates and patterns in the co-infection of viruses. The subtle differences in GmmIV and GmmNegeV in our study are perhaps because they do not replicate at the same rate in Gmm or that they compete for some resources (e.g., nutrition). Studies on co-infecting viruses have shown that competition can occur during replication or virulence in mosquito experiments [18,27,28,29], opening up the possibility that this may occur in tsetse flies as well. Furthermore, the increase in GmmNegeV density with the increasing age of the fly generally showed that older flies (adults in our study) have higher densities of viruses. A study on biting midges (*Culicoides sonorensis*) also showed that increasing age correlates with increasing whole body virus densities [79]. However, another study showed increased densities of some bacteria in mature flies, comparing teneral flies in an irradiation experiment on the Queensland fruit fly [55]. In mosquitoes, age and radiation were shown to affect the microbiome, with age having the highest impact in triggering changes in diversity of the bacteriome [54]. Increasing density with age may also have been contributed by compromised immunity (declining immunity), as observed in a study where the immune defence against infections, including bacteria, declined with age in *Drosophila* [80,81], a possible cause of increased infections in the adult flies.

Unlike GmmIV, the density of GmmNegeV tended to be higher, although none of the tested parameters showed a significant effect, suggesting their ability to thrive in different conditions Some viruses have been shown to thrive in hypoxia while others thrive in oxygen (as they are strictly aerobic, e.g., influenza viruses) and others are in between (aero–anaerobic) [82]. Adenoviruses’ (tumour-selective viruses with the potential for treating cancers in humans) replication was inhibited and the herpes simplex virus replication was enhanced in a hypoxic tumour microenvironment [83]. The effects of short-term pre-conditioning in hypoxia (1 h in hypoxia before irradiation in hypoxia in this study) likely lasted longer than the irradiation day, given the higher virus density in adults. Additionally, hypoxia may enhance or compromise the immunity of tsetse flies to promote or inhibit the virus replication, as short-term pre-conditioning in hypoxia (up to 1 h) has been reported as hormetic in other irradiation studies [60,61,84]. The pre-conditioning in hypoxia acts as a mild stress, signalling the immune system to increase the production of antioxidants, which protect the fly during irradiation [85], differing from prolonged hypoxia, which acts as a stressor resulting in detrimental effects as seen in tephritids [86]. The existence of the antioxidant pathway in tsetse flies has been proven in other studies [87,88] and may have similar effects on the viruses in tsetse flies (GmmIV and GmmNegeV in this study), e.g., increased GmmNegeV densities at the adult stage downregulating the antioxidant genes over the days post irradiation. Increased antioxidants due to the hypoxic conditions may also have favoured the replication of the virus as observed in tsetse flies, where they promoted trypanosome establishment [89]. Tsetse flies have also been shown to mount immune responses against SGHV through the RNAi mechanism and antioxidant pathways to the point of maintaining homeostasis within cells and, therefore, maintaining the latency of the virus [76,77]. As previously mentioned, deteriorating immunity and increased infection with increasing age has also been observed in *Drosophila* [25,26]. Additionally, aging contributed to immuno-senescence, predisposing organisms to infection [37]. Evidently, in our study, the pre-conditioning in hypoxia reduced the impact of irradiation on both the GmmIV and GmmNegeV (perhaps due to the increased immunity of Gmm due to antioxidant production), as there was no evidence for increasing densities due to irradiation doses. Consequently, higher doses (as also required to achieve sterility in hypoxic conditions) may be required before the compromised immunity is evident and/or the virus densities increase due to irradiation.

The lack of differences in viral densities caused by irradiation doses in our study may mean that irradiation was hormetic in cases where the virus tended to increase (e.g., at 150 Gy in both viruses). It may also be evidence that these viruses are highly radioresistant (against destruction of the virions), as found in serum inactivation protocols where extremely high doses (25 KGy) are needed [90]. Sanitization of the combs of honey bees also showed that very high doses (25 KGy) are required to reduce viral densities [91]. The radio resistance of these viruses may also be attributed to the size of their genome (as previously described) [43]. Smaller viruses have been reported to be highly radioresistant compared to larger ones [90]. Despite our observations, evidence for insects negatively affected by irradiation is abundant in studies of the morphology, physiology and performance of tsetse flies and other irradiated insects [92,93,94]. The midgut ultrastructure of tsetse species (*Glossina palpalis palpalis* and *Glossina palpalis gambiensis*) irradiated at a dose of 130 Gy showed damaged cells and tissues due to irradiation, which compromised the immunity of the flies in preventing trypanosoma infections [92]. Additionally, *Spodoptera littoralis* pupae irradiated at different doses and infected with the *Spodoptera littoralis* nucleopolyhedrovirus (SpliNPV) revealed a high sensitivity to different concentrations of the virus with increasing irradiation dose, resulting in an increased percentage of mortality [53], thus revealing the compromised immunity of the *S. littoralis* pupae. Similarly, while the currently used irradiation doses may not affect the virus densities, it seems that the presence of other stressors (i.e., longevity (life stages/dpi) and irradiation conditions (hypoxia/normoxia)) may expose and exacerbate the effects of irradiation doses, as seen in some instances in our study (e.g., irradiation of GmmNegeV in normoxia), through additive and interactive effects. This agrees with other studies on the synergistic effects of stressors [86,95].

## 5. Conclusions

In summary, both the GmmIV and the GmmNegeV are present and stable in Gmm, although the current triggers of disease symptomatic infections remain unknown. The differing responses of GmmIV and GmmNegeV to the effect of various parameters in this study are evidence of their independence in pathogenesis in Gmm, as also found in a previous study where they replicated and localised in different cells (e.g., adipocytes) [43]. Therefore, each virus may respond to different immune pathways and will need different control methods. Furthermore, the general lack of variation in virus densities attributed to irradiation doses when irradiation was conducted in hypoxia (especially for GmmNegeV) shows that it is preferable to irradiate in hypoxia. Our findings are significant for SIT, as irradiation at the current doses clearly does not vary the densities from the status quo and, hence, concerns of increased viral densities from the initial infections should be limited. As the GmmIV and GmmNegeV are newly discovered viruses in Gmm, this forms the first report of the virus densities after irradiation at different doses, in different irradiation conditions (normoxia/hypoxia) and at different life stages, including the days post irradiation (dpi). It is also the first report on the impact of irradiation on the known virome of tsetse flies, among many other irradiated insects.

The effects of irradiation on the immune response of the Gmm towards the two viruses, as well as the level of destruction caused to the virion during the irradiation of tsetse flies, remain to be elucidated. Moreover, the impacts of these viral infections on the fitness and performance of Gmm and other tsetse species, taking into consideration various stressors that may trigger symptomatic infections, are needed to confirm our findings. The impacts of the irradiation doses 170 and 190 Gy on GmmIV and GmmNegeV densities are limited to a small indication of what the effect of irradiation at higher doses in hypoxia is like, as there is only one replicate of each in our study. Further sampling at these high doses and all the doses in general is needed in future studies to confirm conclusions in this study.

## Figures and Tables

**Table 1:** Primers used for quantitative PCR.

Gene/Genome	Primer Name	Sequence 5′ to 3′ End
β-tubulin (Tsetse)	Tse-TubqPCR-F	GATGGTCAAGTGCGATCCT
β-tubulin (Tsetse)	Tse-TubqPCR-R	TGAGAACTCGCCTTCTTCC
Iflavirus (Gmm)	Ifla_qPCR2_7848F	AGAAATTGAAGGACAGATGTTTGGT
Iflavirus (Gmm)	Ifla_qPCR2_7947R	ACCTAAGAAATTACCAGTACCCTCC
Negevirus (Gmm)	Nege-qPCR1-2411F	CAACATAGACTTGAACCAGAGCA
Negevirus (Gmm)	Nege-qPCR1-2529R	GAAACATCAAACACACTCCCATTAG

## Data Availability

The underlying data of this study have been deposited at https://dataverse.harvard.edu/dataset.xhtml?persistentId=doi:10.7910/DVN/ERXAB3, deposited 10 March 2023.

## Supplementary Materials

The following supporting information can be downloaded at: https://www.mdpi.com/article/10.3390/insects14040397/s1, Supplementary File S1: Evaluating the effect of irradiation on the densities of two RNA viruses in *Glossina morsitans morsitans*; Figure S1: Materials and equipment to apply a hypoxic environment before irradiation of pupae; Figure S2: Effect irradiation doses on the density of GmmIV in different life stage of *G. morsitans morsitans* following irradiation in different irradiation conditions; Figure S3: Effect irradiation doses on the density of GmmNegeV in different life stage of G. morsitans morsitans following irradiation in different irradiation conditions.
