# Supplementary material for: Evaluating the Effect of Irradiation on the Densities of Two RNA Viruses in Glossina morsitans morsitans
Source: Insects. 2023 Apr 20;14(4):397. doi: 10.3390/insects14040397 (PMC10140815; doi:10.3390/insects14040397)
Supplement: Supplementary file 1 [file insects-14-00397-s001.zip › S1_Numbered_April2023.pdf]

Evaluating the effect of irradiation on the densities of two RNA viruses in Glossina morsitans morsitans

Mirieri, C.K., et al.

13-7-2022

```
#setwd("D:/analysis/iflanegemanuscript2023/final")

library(ggplot2)
library(MASS)
library(rmarkdown)
library(knitr)
library(lme4)

## Loading required package: Matrix

library(MuMIn)
library(ggthemes) # Load
library(datasets)
library(plyr)
library(dplyr)

##
## Attaching package: 'dplyr'

## The following objects are masked from 'package:plyr':
##
##     arrange, count, desc, failwith, id, mutate, rename, summarise,
##     summarize

## The following object is masked from 'package:MASS':
##
##     select

## The following objects are masked from 'package:stats':
##
##     filter, lag

## The following objects are masked from 'package:base':
##
##     intersect, setdiff, setequal, union

library(tidyverse)

## ── Attaching packages ───────────────────────────────────────
tidyverse 1.3.1 ──
```

```
## ✔ tibble  3.1.7     ✔ purrr   0.3.4
## ✔ tidyr   1.2.0     ✔ stringr 1.4.0
## ✔ readr   2.1.2     ✔ forcats 0.5.1

## ── Conflicts ──────────────────────────────────────────
tidyverse_conflicts() ──
## ✖ dplyr::arrange()   masks plyr::arrange()
## ✖ purrr::compact()   masks plyr::compact()
## ✖ dplyr::count()     masks plyr::count()
## ✖ tidyr::expand()    masks Matrix::expand()
## ✖ dplyr::failwith()  masks plyr::failwith()
## ✖ dplyr::filter()    masks stats::filter()
## ✖ dplyr::id()        masks plyr::id()
## ✖ dplyr::lag()       masks stats::lag()
## ✖ dplyr::mutate()    masks plyr::mutate()
## ✖ tidyr::pack()      masks Matrix::pack()
## ✖ dplyr::rename()    masks plyr::rename()
## ✖ dplyr::select()    masks MASS::select()
## ✖ dplyr::summarise() masks plyr::summarise()
## ✖ dplyr::summarize() masks plyr::summarize()
## ✖ tidyr::unpack()    masks Matrix::unpack()
```

Working directory

```
#-------------------------------------------------------

##Normality for neg, b and C

#Normality for neg

qpcr <- read.csv("Iflaneg_may_2023.csv",sep=",", row.names=NULL)

#qpcr$Life_stage=as.factor(qpcr$Life_stage)
qpcr$Irradiation_dose=as.factor(qpcr$Irradiation_dose)
str(qpcr)

## 'data.frame':    186 obs. of  11 variables:
##  $ Target                : chr  "GmmIV" "GmmIV" "GmmIV" "GmmIV" ...
##  $ Sample                : chr  "17A" "17B" "18A" "18B" ...
##  $ Irradiation_dose      : Factor w/ 5 levels "0","70","110",..: 1 1
1 1 1 1 1 1 2 2 ...
##  $ Dpi                   : int  3 3 3 3 0 0 0 0 3 3 ...
##  $ Life_stage            : chr  "Adult (3 dpi)" "Adult (3 dpi)"
"Adult (3 dpi)" "Adult (3 dpi)" ...
##  $ Irradiation_conditions: chr  "Hypoxia" "Hypoxia" "Normoxia"
"Normoxia" ...
##  $ Replicate             : int  1 1 1 1 1 1 1 1 1 1 ...
##  $ Expression            : num  0.109 0.125 0.135 0.125 1.013 ...
```

```
##  $ Mean.Cq               : num  22 22.2 21.7 21.9 20.1 ...
##  $ Normalized_expression : num  0.46 0.529 0.569 0.529 3.096 ...
##  $ Transformed           : num  -0.806 -0.658 -0.581 -0.657 1.069
...

attach(qpcr)
#head(neg)
qpcr=na.omit(qpcr)
qpcr

##      Target Sample Irradiation_dose Dpi    Life_stage
Irradiation_conditions
## 1     GmmIV    17A                0   3 Adult (3 dpi)
Hypoxia
## 2     GmmIV    17B                0   3 Adult (3 dpi)
Hypoxia
## 3     GmmIV    18A                0   3 Adult (3 dpi)
Normoxia
## 4     GmmIV    18B                0   3 Adult (3 dpi)
Normoxia
## 5     GmmIV     8A                0   0 Pupae (0 dpi)
Hypoxia
## 6     GmmIV     8B                0   0 Pupae (0 dpi)
Hypoxia
## 7     GmmIV     9A                0   0 Pupae (0 dpi)
Normoxia
## 8     GmmIV     9B                0   0 Pupae (0 dpi)
Normoxia
## 9     GmmIV    11A               70   3 Adult (3 dpi)
Hypoxia
## 10    GmmIV    11B               70   3 Adult (3 dpi)
Hypoxia
## 11    GmmIV    14A               70   3 Adult (3 dpi)
Normoxia
## 12    GmmIV    14B               70   3 Adult (3 dpi)
Normoxia
## 13    GmmIV     2A               70   0 Pupae (0 dpi)
Hypoxia
## 14    GmmIV     2B               70   0 Pupae (0 dpi)
Hypoxia
## 15    GmmIV     5A               70   0 Pupae (0 dpi)
Normoxia
## 16    GmmIV     5B               70   0 Pupae (0 dpi)
Normoxia
## 17    GmmIV    12A              110   3 Adult (3 dpi)
Hypoxia
## 18    GmmIV    12B              110   3 Adult (3 dpi)
Hypoxia
## 19    GmmIV    15A              110   3 Adult (3 dpi)
Normoxia
```

```
## 20    GmmIV    15B               110   3 Adult (3 dpi)
Normoxia
## 21    GmmIV     3A               110   0 Pupae (0 dpi)
Hypoxia
## 22    GmmIV     3B               110   0 Pupae (0 dpi)
Hypoxia
## 23    GmmIV     6A               110   0 Pupae (0 dpi)
Normoxia
## 24    GmmIV     6B               110   0 Pupae (0 dpi)
Normoxia
## 25    GmmIV    13A               150   3 Adult (3 dpi)
Hypoxia
## 26    GmmIV    13B               150   3 Adult (3 dpi)
Hypoxia
## 27    GmmIV    16B               150   3 Adult (3 dpi)
Normoxia
## 28    GmmIV     4A               150   0 Pupae (0 dpi)
Hypoxia
## 29    GmmIV     4B               150   0 Pupae (0 dpi)
Hypoxia
## 30    GmmIV     7A               150   0 Pupae (0 dpi)
Normoxia
## 31    GmmIV     7B               150   0 Pupae (0 dpi)
Normoxia
## 32    GmmIV     1A                 0   0 Pupae (0 dpi)
Normoxia
## 33    GmmIV     1B                 0   0 Pupae (0 dpi)
Normoxia
## 34    GmmIV     1A                 0   0 Pupae (0 dpi)
Hypoxia
## 35    GmmIV     1b                 0   0 Pupae (0 dpi)
Hypoxia
## 36    GmmIV     2a                 0   3 Adult (3 dpi)
Hypoxia
## 37    GmmIV     2b                 0   3 Adult (3 dpi)
Hypoxia
## 38    GmmIV     3a                 0   3 Adult (3 dpi)
Normoxia
## 39    GmmIV     3b                 0   3 Adult (3 dpi)
Normoxia
## 40    GmmIV    21A                70   0 Pupae (0 dpi)
Normoxia
## 41    GmmIV    21B                70   0 Pupae (0 dpi)
Normoxia
## 42    GmmIV     3A                70   3 Adult (3 dpi)
Hypoxia
## 43    GmmIV     3B                70   3 Adult (3 dpi)
Hypoxia
## 44    GmmIV     6A                70   3 Adult (3 dpi)
Normoxia
```

```
## 45    GmmIV     6B                70   3 Adult (3 dpi)
Normoxia
## 46    GmmIV     2A                70   0 Pupae (0 dpi)
Hypoxia
## 47    GmmIV     2B                70   0 Pupae (0 dpi)
Hypoxia
## 48    GmmIV    22A               110   0 Pupae (0 dpi)
Normoxia
## 49    GmmIV    22B               110   0 Pupae (0 dpi)
Normoxia
## 50    GmmIV     4A               110   3 Adult (3 dpi)
Hypoxia
## 51    GmmIV     4B               110   3 Adult (3 dpi)
Hypoxia
## 52    GmmIV     7A               110   3 Adult (3 dpi)
Normoxia
## 53    GmmIV     7B               110   3 Adult (3 dpi)
Normoxia
## 54    GmmIV     3A               110   0 Pupae (0 dpi)
Hypoxia
## 55    GmmIV     3B               110   0 Pupae (0 dpi)
Hypoxia
## 56    GmmIV    20A               150   0 Pupae (0 dpi)
Hypoxia
## 57    GmmIV    20B               150   0 Pupae (0 dpi)
Hypoxia
## 58    GmmIV    23A               150   0 Pupae (0 dpi)
Normoxia
## 59    GmmIV    23B               150   0 Pupae (0 dpi)
Normoxia
## 60    GmmIV     5A               150   3 Adult (3 dpi)
Hypoxia
## 61    GmmIV     5B               150   3 Adult (3 dpi)
Hypoxia
## 62    GmmIV     8A               150   3 Adult (3 dpi)
Normoxia
## 63    GmmIV     8B               150   3 Adult (3 dpi)
Normoxia
## 64    GmmIV    16A                 0   0 Pupae (0 dpi)
Hypoxia
## 65    GmmIV    16B                 0   0 Pupae (0 dpi)
Hypoxia
## 66    GmmIV    17A                 0   3 Adult (3 dpi)
Normoxia
## 67    GmmIV    17B                 0   3 Adult (3 dpi)
Normoxia
## 68    GmmIV     4a                 0   3 Adult (3 dpi)
Hypoxia
## 69    GmmIV     4b                 0   3 Adult (3 dpi)
Hypoxia
```

```
## 70    GmmIV    10A                 110   0 Pupae (0 dpi)
Hypoxia
## 71    GmmIV    10B                 110   0 Pupae (0 dpi)
Hypoxia
## 72    GmmIV    13A                  70   0 Pupae (0 dpi)
Normoxia
## 73    GmmIV    13B                  70   0 Pupae (0 dpi)
Normoxia
## 74    GmmIV    19A                 110   3 Adult (3 dpi)
Hypoxia
## 75    GmmIV    19B                 110   3 Adult (3 dpi)
Hypoxia
## 76    GmmIV    22A                  70   3 Adult (3 dpi)
Normoxia
## 77    GmmIV    22B                  70   3 Adult (3 dpi)
Normoxia
## 78    GmmIV    11A                 150   0 Pupae (0 dpi)
Hypoxia
## 79    GmmIV    14A                 110   0 Pupae (0 dpi)
Normoxia
## 80    GmmIV    14B                 110   0 Pupae (0 dpi)
Normoxia
## 81    GmmIV    20A                 150   3 Adult (3 dpi)
Hypoxia
## 82    GmmIV    20B                 150   3 Adult (3 dpi)
Hypoxia
## 83    GmmIV     4A                 110   3 Adult (3 dpi)
Normoxia
## 84    GmmIV     4B                 110   3 Adult (3 dpi)
Normoxia
## 85    GmmIV    12A                 190   0 Pupae (0 dpi)
Hypoxia
## 86    GmmIV    12B                 190   0 Pupae (0 dpi)
Hypoxia
## 87    GmmIV    15A                 150   0 Pupae (0 dpi)
Normoxia
## 88    GmmIV    15B                 150   0 Pupae (0 dpi)
Normoxia
## 89    GmmIV    21A                 190   3 Adult (3 dpi)
Hypoxia
## 90    GmmIV    21B                 190   3 Adult (3 dpi)
Hypoxia
## 91    GmmIV     5A                 150   3 Adult (3 dpi)
Normoxia
## 92    GmmIV     5B                 150   3 Adult (3 dpi)
Normoxia
## 93  GmmNegV    17A                   0   3 Adult (3 dpi)
Hypoxia
## 94  GmmNegV    17B                   0   3 Adult (3 dpi)
Hypoxia
```

```
## 95  GmmNegV    18A                 0   3 Adult (3 dpi)
Normoxia
## 96  GmmNegV    18B                 0   3 Adult (3 dpi)
Normoxia
## 97  GmmNegV     8A                 0   0 Pupae (0 dpi)
Hypoxia
## 98  GmmNegV     8B                 0   0 Pupae (0 dpi)
Hypoxia
## 99  GmmNegV     9A                 0   0 Pupae (0 dpi)
Normoxia
## 100 GmmNegV     9B                 0   0 Pupae (0 dpi)
Normoxia
## 101 GmmNegV    16B                 0   0 Pupae (0 dpi)
Hypoxia
## 102 GmmNegV    16B                 0   0 Pupae (0 dpi)
Hypoxia
## 103 GmmNegV    17A                 0   3 Adult (3 dpi)
Normoxia
## 104 GmmNegV    17B                 0   3 Adult (3 dpi)
Normoxia
## 105 GmmNegV     6A                 0   3 Adult (3 dpi)
Normoxia
## 106 GmmNegV     6B                 0   3 Adult (3 dpi)
Normoxia
## 107 GmmNegV     1A                 0   0 Pupae (0 dpi)
Hypoxia
## 108 GmmNegV     1B                 0   0 Pupae (0 dpi)
Hypoxia
## 109 GmmNegV     2A                 0   3 Adult (3 dpi)
Hypoxia
## 110 GmmNegV     2B                 0   3 Adult (3 dpi)
Hypoxia
## 111 GmmNegV     3a                 0   3 Adult (3 dpi)
Normoxia
## 112 GmmNegV     3b                 0   3 Adult (3 dpi)
Normoxia
## 113 GmmNegV     4a                 0   3 Adult (3 dpi)
Hypoxia
## 114 GmmNegV     4b                 0   3 Adult (3 dpi)
Hypoxia
## 115 GmmNegV    11A                70   3 Adult (3 dpi)
Hypoxia
## 116 GmmNegV    11B                70   3 Adult (3 dpi)
Hypoxia
## 117 GmmNegV    14A                70   3 Adult (3 dpi)
Normoxia
## 118 GmmNegV    14B                70   3 Adult (3 dpi)
Normoxia
## 119 GmmNegV    21A                70   0 Pupae (0 dpi)
Normoxia
```

```
## 120 GmmNegV    21B                  70   0 Pupae (0 dpi)
Normoxia
## 121 GmmNegV     2A                  70   0 Pupae (0 dpi)
Hypoxia
## 122 GmmNegV     2B                  70   0 Pupae (0 dpi)
Hypoxia
## 123 GmmNegV     5A                  70   0 Pupae (0 dpi)
Normoxia
## 124 GmmNegV     5B                  70   0 Pupae (0 dpi)
Normoxia
## 125 GmmNegV    10A                 110   0 Pupae (0 dpi)
Hypoxia
## 126 GmmNegV    10B                 110   0 Pupae (0 dpi)
Hypoxia
## 127 GmmNegV    13A                  70   0 Pupae (0 dpi)
Normoxia
## 128 GmmNegV    13B                  70   0 Pupae (0 dpi)
Normoxia
## 129 GmmNegV    19A                 110   3 Adult (3 dpi)
Hypoxia
## 130 GmmNegV    19B                 110   3 Adult (3 dpi)
Hypoxia
## 131 GmmNegV    22A                  70   3 Adult (3 dpi)
Normoxia
## 132 GmmNegV    22B                  70   3 Adult (3 dpi)
Normoxia
## 133 GmmNegV     3A                  70   3 Adult (3 dpi)
Hypoxia
## 134 GmmNegV     3B                  70   3 Adult (3 dpi)
Hypoxia
## 135 GmmNegV     6A                  70   3 Adult (3 dpi)
Normoxia
## 136 GmmNegV     6B                  70   3 Adult (3 dpi)
Normoxia
## 137 GmmNegV     2A                  70   0 Pupae (0 dpi)
Hypoxia
## 138 GmmNegV     2B                  70   0 Pupae (0 dpi)
Hypoxia
## 139 GmmNegV    12A                 110   3 Adult (3 dpi)
Hypoxia
## 140 GmmNegV    12B                 110   3 Adult (3 dpi)
Hypoxia
## 141 GmmNegV    15A                 110   3 Adult (3 dpi)
Normoxia
## 142 GmmNegV    15B                 110   3 Adult (3 dpi)
Normoxia
## 143 GmmNegV    22A                 110   0 Pupae (0 dpi)
Normoxia
## 144 GmmNegV    22B                 110   0 Pupae (0 dpi)
Normoxia
```

```
## 145 GmmNegV      3A              110   0 Pupae (0 dpi)
Hypoxia
## 146 GmmNegV      3B              110   0 Pupae (0 dpi)
Hypoxia
## 147 GmmNegV      6A              110   0 Pupae (0 dpi)
Normoxia
## 148 GmmNegV      6B              110   0 Pupae (0 dpi)
Normoxia
## 149 GmmNegV     11A              150   0 Pupae (0 dpi)
Hypoxia
## 150 GmmNegV     11B              150   0 Pupae (0 dpi)
Hypoxia
## 151 GmmNegV     14A              110   0 Pupae (0 dpi)
Normoxia
## 152 GmmNegV     14B              110   0 Pupae (0 dpi)
Normoxia
## 153 GmmNegV     20A              150   3 Adult (3 dpi)
Hypoxia
## 154 GmmNegV     20B              150   3 Adult (3 dpi)
Hypoxia
## 155 GmmNegV      4A              110   3 Adult (3 dpi)
Hypoxia
## 156 GmmNegV      4B              110   3 Adult (3 dpi)
Hypoxia
## 157 GmmNegV      7A              110   3 Adult (3 dpi)
Normoxia
## 158 GmmNegV      7B              110   3 Adult (3 dpi)
Normoxia
## 159 GmmNegV      3A              110   0 Pupae (0 dpi)
Hypoxia
## 160 GmmNegV      3B              110   0 Pupae (0 dpi)
Hypoxia
## 161 GmmNegV      4A              110   3 Adult (3 dpi)
Normoxia
## 162 GmmNegV      4B              110   3 Adult (3 dpi)
Normoxia
## 163 GmmNegV     13A              150   3 Adult (3 dpi)
Hypoxia
## 164 GmmNegV     13B              150   3 Adult (3 dpi)
Hypoxia
## 165 GmmNegV     16A              150   3 Adult (3 dpi)
Normoxia
## 166 GmmNegV     16B              150   3 Adult (3 dpi)
Normoxia
## 167 GmmNegV     20A              150   0 Pupae (0 dpi)
Hypoxia
## 168 GmmNegV     20B              150   0 Pupae (0 dpi)
Hypoxia
## 169 GmmNegV     23A              150   0 Pupae (0 dpi)
Normoxia
```

```
## 170 GmmNegV    23B              150   0 Pupae (0 dpi)
Normoxia
## 171 GmmNegV     4A              150   0 Pupae (0 dpi)
Hypoxia
## 172 GmmNegV     4B              150   0 Pupae (0 dpi)
Hypoxia
## 173 GmmNegV     7A              150   0 Pupae (0 dpi)
Normoxia
## 174 GmmNegV     7B              150   0 Pupae (0 dpi)
Normoxia
## 175 GmmNegV    12A              190   0 Pupae (0 dpi)
Hypoxia
## 176 GmmNegV    12B              190   0 Pupae (0 dpi)
Hypoxia
## 177 GmmNegV    21A              190   3 Adult (3 dpi)
Hypoxia
## 178 GmmNegV    21B              190   3 Adult (3 dpi)
Hypoxia
## 179 GmmNegV     5A              150   3 Adult (3 dpi)
Hypoxia
## 180 GmmNegV     5B              150   3 Adult (3 dpi)
Hypoxia
## 181 GmmNegV     8A              150   3 Adult (3 dpi)
Normoxia
## 182 GmmNegV     8B              150   3 Adult (3 dpi)
Normoxia
## 183 GmmNegV     5A              150   3 Adult (3 dpi)
Normoxia
## 184 GmmNegV     5B              150   3 Adult (3 dpi)
Normoxia
## 185 GmmNegV     1A                0   0 Pupae (0 dpi)
Normoxia
## 186 GmmNegV     1B                0   0 Pupae (0 dpi)
Normoxia
##     Replicate  Expression  Mean.Cq Normalized_expression Transformed
## 1           1   0.1090300 22.03000            0.46045991 -0.80639449
## 2           1   0.1252000 22.18000            0.52874971 -0.65798208
## 3           1   0.1346500 21.74000            0.56865933 -0.58070931
## 4           1   0.1253500 21.86000            0.52938320 -0.65670601
## 5           1   1.0128188 20.06000            3.09645293  1.06872315
## 6           1   0.9783009 20.14000            2.99092264  1.03769990
## 7           1   4.2774600 20.65000           13.07731817  2.26699785
## 8           1   0.2131900 24.97000            0.65177780 -0.43734511
## 9           1   0.1312300 21.22000            0.55421585 -0.60796567
## 10          1   0.0281800 23.20000            0.11901092 -2.37204012
## 11          1  51.4884900 23.08000          217.44827590  4.16199580
## 12          1  35.4320700 23.73000          149.63815280  3.93967708
## 13          1   0.3535200 23.98000            1.08080345  0.07740358
## 14          1   0.5363300 23.34000            1.63970161  0.48248615
## 15          1   1.1104700 21.11000            3.39499832  1.15055481
```

```
## 16           1   0.7600200 21.47000             2.32358067  0.80854586
## 17           1   0.1811900 21.22000             0.76520895 -0.27121916
## 18           1   0.1300200 21.37000             0.54910573 -0.61779662
## 19           1   0.1700800 20.48000             0.71828874 -0.33641872
## 20           1   0.1377400 20.66000             0.58170915 -0.55672992
## 21           1   1.0421000 21.21000             3.18597328  1.09414157
## 22           1   0.8134100 21.31000             2.48680791  0.87073579
## 23           1   0.3025000 22.33000             0.92482191 -0.07846029
## 24           1   0.1771300 22.62000             0.54153291 -0.63255198
## 25           1   0.1016900 21.38000             0.42946133 -0.88197175
## 26           1   0.0811200 21.68000             0.34258927 -1.13070376
## 27           1   0.0890500 22.69000             0.37607957 -1.02737202
## 28           1   0.3971000 21.14000             1.21403895  0.19208399
## 29           1   0.2948100 21.36000             0.90131157 -0.10444596
## 30           1   3.0791500 19.95000             9.41376991  2.00858578
## 31           1   0.9467200 20.14000             2.89437158  1.00824280
## 32           2  41.6958900 18.76000             1.52845712  0.41538497
## 33           2  44.1936500 18.90000             1.62001816  0.47098498
## 34           2   4.9830300 20.88000             0.18266423 -1.85317370
## 35           2   4.0377800 21.10000             0.14801396 -2.10513769
## 36           2   2.4963200 21.57000             1.12002369  0.11270985
## 37           2   2.4351600 21.58000             1.09258304  0.08815380
## 38           2   2.4411800 21.48000             1.09528403  0.09060080
## 39           2   1.7750200 21.63000             0.79639808 -0.23026726
## 40           2  77.4999800 18.05000             2.84093700  0.99147185
## 41           2  89.1371500 18.02000             3.26752377  1.11662237
## 42           2   0.3540300 21.12000             0.15884261 -2.01996763
## 43           2   0.2905300 21.09000             0.13035207 -2.25993610
## 44           2   0.7990400 21.06000             0.35850521 -1.08027279
## 45           2   0.5569800 21.31000             0.24990017 -1.48744234
## 46           2  85.4123400 18.18000             3.13098244  1.07862208
## 47           2  51.9678200 18.22000             1.90499794  0.62415220
## 48           2  77.0431500 18.06000             2.82419086  0.98614442
## 49           2  70.0133600 18.17000             2.56649801  0.89948632
## 50           2   1.5843400 20.05000             0.71084570 -0.34719100
## 51           2   1.4308200 19.84000             0.64196589 -0.45318904
## 52           2   0.7566700 25.64000             0.33949507 -1.14080706
## 53           2   0.4437700 25.93000             0.19910625 -1.75145145
## 54           2  70.2479300 17.60000             2.57509671  0.90252972
## 55           2  54.1456100 17.67000             1.98482976  0.66256323
## 56           2  63.0303700 19.38000             2.31052073  0.80336367
## 57           2  55.6890600 19.66000             2.04140841  0.68877095
## 58           2 131.2552700 17.80000             4.81145869  1.45381433
## 59           2 103.3295600 18.06000             3.78777865  1.24690704
## 60           2  14.0394200 23.05000             6.29906542  1.68097579
## 61           2   8.8575800 23.14000             3.97412969  1.28884390
## 62           2   1.0936700 21.94000             0.49069683 -0.73788318
## 63           2   0.7212700 21.90000             0.32361215 -1.19431485
## 64           3  14.4368800 17.24000             1.32861836  0.28014077
## 65           3  13.5849800 17.28000             1.25021845  0.22084321
```

```
## 66            3   9.0625900 17.96000            75.16455171  3.50769831
## 67            3   8.3693500 18.03000            69.41486274  3.45582728
## 68            3  11.9261400 19.77000            98.91465539  3.68353730
## 69            3   3.4096000 21.27000            28.27900805  2.84097988
## 70            3   5.3506800 18.51000             0.49242022 -0.73411916
## 71            3   5.9361300 18.40000             0.54629887 -0.62323942
## 72            3   5.9905200 17.58000             0.55130436 -0.61355458
## 73            3   5.4069100 17.79000             0.49759504 -0.72290345
## 74            3   0.5584300 20.94000             4.63158331  1.42118995
## 75            3   0.4598800 21.13000             3.81421581  1.25299301
## 76            3   0.2825900 19.52000             2.34378369  0.81649964
## 77            3   0.2831800 19.69000             2.34867712  0.81841480
## 78            3  11.0635400 18.09000             1.01817168  0.01799234
## 79            3  15.4973400 18.25000             1.42621193  0.34879382
## 80            3   7.1197500 18.13000             0.65522679 -0.43183803
## 81            3   0.2620600 19.74000             2.17350917  0.74697251
## 82            3   0.2224400 20.03000             1.84490338  0.59405061
## 83            3   0.3690400 19.24000             3.06079456  1.05837233
## 84            3   0.2667800 19.50000             2.21265655  0.76347523
## 85            3   7.8034500 17.96000             0.71814734 -0.33662223
## 86            3   3.7812600 18.02000             0.34798734 -1.11331580
## 87            3  13.1375900 17.86000             1.20904539  0.18804067
## 88            3  13.0945400 17.75000             1.20508352  0.18481962
## 89            3   0.1813300 19.87000             1.50393962  0.39987341
## 90            3   0.1838700 20.09000             1.52500622  0.41321829
## 91            3   0.8061700 18.81000             6.68632330  1.73046170
## 92            3   0.4974200 19.22000             4.12557021  1.32136144
## 93            1   0.8102000 26.68000             5.20627169  1.52094764
## 94            1   0.7363400 26.76000             4.73165403  1.43950847
## 95            1   0.5395100 26.91000             3.46684231  1.16906697
## 96            1   0.4863700 27.07000             3.12536949  1.07702116
## 97            1   0.8843100 27.01000             0.26036264 -1.44042433
## 98            1   1.1734000 26.50000             0.34547786 -1.12136202
## 99            1   2.6828300 28.13000             0.78989122 -0.23866353
## 100           1   4.7126700 28.02000             1.38752611  0.32221692
## 101           3  10.7132300 24.32000             2.31479499  0.80506324
## 102           3   8.7765400 24.43000             1.89633666  0.61987867
## 103           3   1.0089800 27.78000             5.75491231  1.60547496
## 104           3   0.9038900 28.06000             5.15551119  1.51263603
## 105           3   0.6253100 25.68000             3.56657636  1.19407774
## 106           3   0.4556300 25.73000             2.59877371  0.91085245
## 107           2   1.4848600 26.97000             0.06325263 -3.17929376
## 108           2   1.4112400 26.96000             0.06011654 -3.24648346
## 109           2   1.8656500 26.34000             0.33823712 -1.14494355
## 110           2   1.6668200 26.47000             0.30218980 -1.27124838
## 111           2   1.1214100 26.94000             0.20330849 -1.72693311
## 112           2   1.0658700 26.71000             0.19323925 -1.78665215
## 113           3   5.4109400 25.25000            30.86234137  2.90328911
## 114           3   4.8051100 25.12000            27.40687295  2.81851841
## 115           1   0.2189000 27.03000             1.40663154  0.33544271
```

```
## 116          1   0.2003100 27.24000            1.28717389  0.24928915
## 117          1 118.9140500 28.95000          764.13089580  4.85147028
## 118          1 105.1212000 29.33000          675.49929310  4.78760271
## 119          2  42.6129100 26.04000            1.81524102  0.57879247
## 120          2  43.9302600 26.02000            1.87135800  0.60743277
## 121          1   1.1265200 29.92000            0.33167523 -1.16679892
## 122          1   0.9836700 29.74000            0.28961667 -1.31924979
## 123          1  11.2072300 25.01000            3.29968452  1.12531886
## 124          1   7.8449400 25.13000            2.30974354  0.80305427
## 125          3   2.4951400 27.69000            0.53912196 -0.63729731
## 126          3   0.6102700 27.92000            0.13186032 -2.24584019
## 127          3   9.2634100 23.79000            2.00153409  0.67038546
## 128          3   4.9444100 24.38000            1.06833285  0.06588137
## 129          3  11.9216400 25.73000           67.99737630  3.44231148
## 130          3   1.8955200 25.77000           10.81146442  2.11845159
## 131          3   0.2362600 26.01000            1.34755454  0.29388652
## 132          3   0.1822200 26.73000            1.03932696  0.03849905
## 133          2   1.0139200 25.99000            0.18382086 -1.84569432
## 134          2   0.9682700 25.82000            0.17554464 -1.90039133
## 135          2   2.0803000 26.05000            0.37715256 -1.02423071
## 136          2   1.8040700 26.21000            0.32707284 -1.18241362
## 137          2 118.9116100 24.16000            5.06544219  1.49766400
## 138          2  68.6617600 24.27000            2.92487988  1.01766607
## 139          1   0.7697600 26.10000            4.94640792  1.47742156
## 140          1   0.6476200 26.37000            4.16154736  1.32889359
## 141          1   0.2797500 26.65000            1.79764812  0.56961265
## 142          1   0.2673800 26.72000            1.71815962  0.52686668
## 143          2  54.4506800 25.55000            2.31951087  0.80693441
## 144          2  48.5452000 25.79000            2.06794697  0.70078990
## 145          1  11.1907100 25.10000            3.29482063  1.12400963
## 146          1  13.0064500 24.81000            3.82941920  1.25647192
## 147          1   1.2239900 26.97000            0.36037280 -1.07451714
## 148          1   1.0268300 27.19000            0.30232404 -1.27074779
## 149          3  17.6618300 24.45000            3.81617081  1.25344121
## 150          3  11.8749900 24.66000            2.56581510  0.89924413
## 151          3   0.3052100 29.31000            0.06594637 -3.12444399
## 152          3   0.8055700 28.08000            0.17405856 -1.91051283
## 153          3   1.2146200 25.03000            6.92781976  1.75975087
## 154          3   0.4163500 25.54000            2.37473264  0.82853891
## 155          2   2.0482300 25.96000            0.37133836 -1.04137139
## 156          2   2.1100900 25.84000            0.38255341 -1.00856706
## 157          2   0.2268300 33.84000            0.04112364 -3.75912578
## 158          2   0.1342400 33.91000            0.02433734 -4.50015672
## 159          2  35.6770200 25.03000            1.51978333  0.40992878
## 160          2  25.7247600 25.20000            1.09583315  0.09109747
## 161          3   0.2381900 26.33000            1.35856267  0.30177999
## 162          3   0.1865100 26.48000            1.06379581  0.06165263
## 163          1   0.5453500 25.99000            3.50436962  1.17856965
## 164          1   0.4553800 26.18000            2.92623056  1.01808076
## 165          1   0.6008200 27.23000            3.86081480  1.26360819
```

```
## 166          1   0.5227800 27.15000          3.35933685  1.14120515
## 167          2 106.8144500 26.32000          4.55012275  1.40595371
## 168          2  74.9821900 26.55000          3.19411998  1.09641565
## 169          2 101.2498400 25.20000          4.31307937  1.35985054
## 170          2 111.2807100 25.17000          4.74037820  1.44108525
## 171          1   0.6616200 27.27000          0.19479722 -1.77719115
## 172          1   0.5530500 26.96000          0.16283154 -1.99019225
## 173          1   3.0770200 25.60000          0.90595047 -0.09926004
## 174          1   2.6007700 25.51000          0.76573074 -0.27051903
## 175          3   6.3921000 25.01000          1.38113352  0.31774685
## 176          3   4.6987000 25.00000          1.01524258  0.01511614
## 177          3   0.2544100 25.98000          1.45107657  0.36546038
## 178          3   0.3049500 26.03000          1.73934122  0.53846673
## 179          2  31.8433400 28.23000          5.77310837  1.60812456
## 180          2  19.2974000 28.22000          3.49856458  1.17710704
## 181          2   5.6814612 26.98748          1.03003300  0.02954710
## 182          2   6.5011374 27.14410          1.17863801  0.16301621
## 183          3   1.1308500 24.78000          6.45002139  1.70065374
## 184          3   0.7980400 25.00000          4.55177527  1.40626577
## 185          2  37.9748200 25.58000          1.61766589  0.46960025
## 186          2  35.9224600 25.59000          1.53023867  0.41650142

# check for normality of the data
Box=boxcox(Normalized_expression~1,  lambda = seq(-35,15,0.1))
```

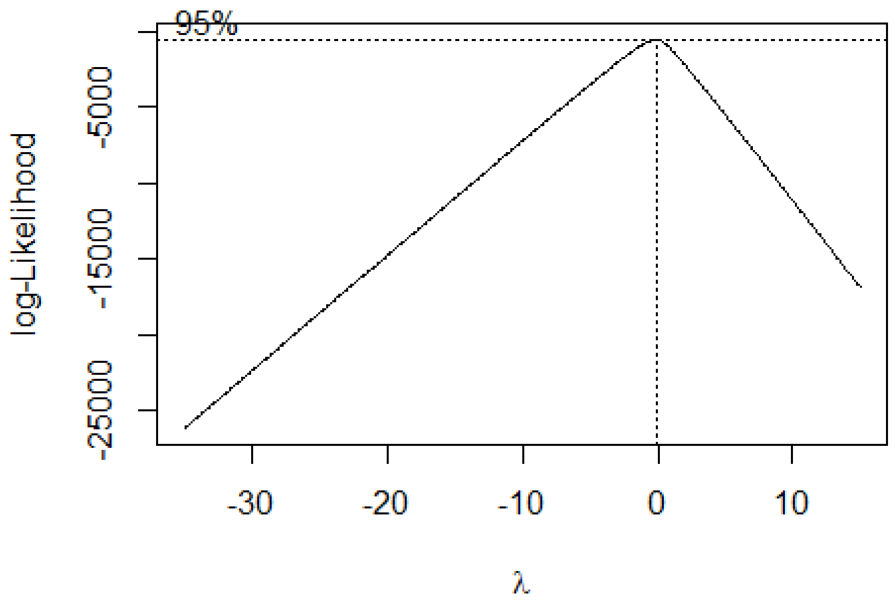

```
Cox = data.frame(Box$x, Box$y)
Cox2 = Cox[with(Cox, order(-Cox$Box.y)),]
Cox2[1,]
```

```
##     Box.x     Box.y
## 350  -0.1 -565.2725
```

```
lambda = Cox2[1, "Box.x"]
lambda
```

```
## [1] -0.1
```

```
Transformed = (Normalized_expression ^ lambda - 1)/lambda
Transformed
```

```
##   [1] -0.80639449 -0.65798208 -0.58070931 -0.65670601  1.06872315
1.03769990
##   [7]  2.26699785 -0.43734511 -0.60796568 -2.37204013  4.16199580
3.93967708
##  [13]  0.07740358  0.48248615  1.15055481  0.80854586 -0.27121916 -
0.61779662
##  [19] -0.33641872 -0.55672992  1.09414157  0.87073579 -0.07846029 -
0.63255198
##  [25] -0.88197175 -1.13070376 -1.02737202  0.19208399 -0.10444596
2.00858578
##  [31]  1.00824280  0.41538497  0.47098498 -1.85317370 -2.10513769
0.11270985
##  [37]  0.08815380  0.09060080 -0.23026726  0.99147185  1.11662237 -
2.01996763
##  [43] -2.25993610 -1.08027279 -1.48744234  1.07862208  0.62415220
0.98614442
##  [49]  0.89948632 -0.34719100 -0.45318904 -1.14080706 -1.75145145
0.90252972
##  [55]  0.66256323  0.80336367  0.68877095  1.45381433  1.24690704
1.68097579
##  [61]  1.28884390 -0.73788318 -1.19431485  0.28014077  0.22084321
3.50769831
##  [67]  3.45582728  3.68353730  2.84097988 -0.73411916 -0.62323942 -
0.61355458
##  [73] -0.72290345  1.42118995  1.25299301  0.81649964  0.81841480
0.01799234
##  [79]  0.34879382 -0.43183803  0.74697251  0.59405061  1.05837233
0.76347523
##  [85] -0.33662223 -1.11331580  0.18804067  0.18481962  0.39987341
0.41321829
##  [91]  1.73046170  1.32136144  1.52094764  1.43950847  1.16906697
1.07702116
##  [97] -1.44042433 -1.12136202 -0.23866353  0.32221692  0.80506324
0.61987867
## [103]  1.60547496  1.51263603  1.19407774  0.91085245 -3.17929376 -
3.24648346
```

```
## [109] -1.14494355 -1.27124838 -1.72693311 -1.78665215  2.90328911
2.81851841
## [115]  0.33544271  0.24928915  4.85147028  4.78760271  0.57879247
0.60743277
## [121] -1.16679892 -1.31924979  1.12531886  0.80305427 -0.63729731 -
2.24584019
## [127]  0.67038546  0.06588137  3.44231148  2.11845159  0.29388652
0.03849905
## [133] -1.84569432 -1.90039133 -1.02423071 -1.18241362  1.49766400
1.01766607
## [139]  1.47742156  1.32889359  0.56961265  0.52686668  0.80693441
0.70078990
## [145]  1.12400963  1.25647192 -1.07451714 -1.27074779  1.25344121
0.89924413
## [151] -3.12444399 -1.91051283  1.75975087  0.82853891 -1.04137139 -
1.00856706
## [157] -3.75912578 -4.50015672  0.40992878  0.09109747  0.30177999
0.06165263
## [163]  1.17856965  1.01808076  1.26360819  1.14120515  1.40595371
1.09641565
## [169]  1.35985054  1.44108525 -1.77719115 -1.99019225 -0.09926004 -
0.27051903
## [175]  0.31774685  0.01511614  0.36546038  0.53846673  1.60812456
1.17710704
## [181]  0.02954710  0.16301621  1.70065374  1.40626577  0.46960025
0.41650142

expression <- with(qpcr,Normalized_expression)
trans <- with(qpcr,Transformed)
library(rcompanion)

plotNormalHistogram(Normalized_expression)
```

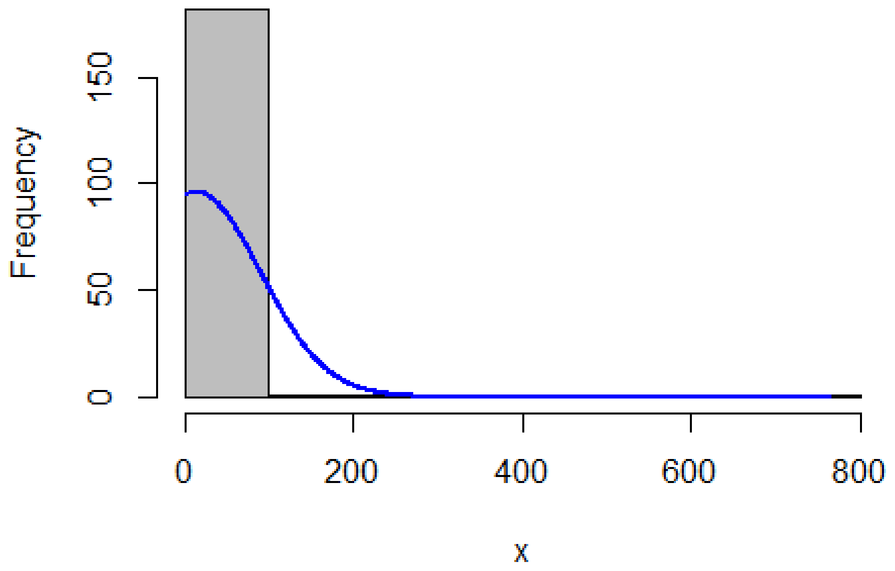


```
qqnorm(Normalized_expression,ylab="Normalized_expression")
qqline(Normalized_expression,
       col="red")
```

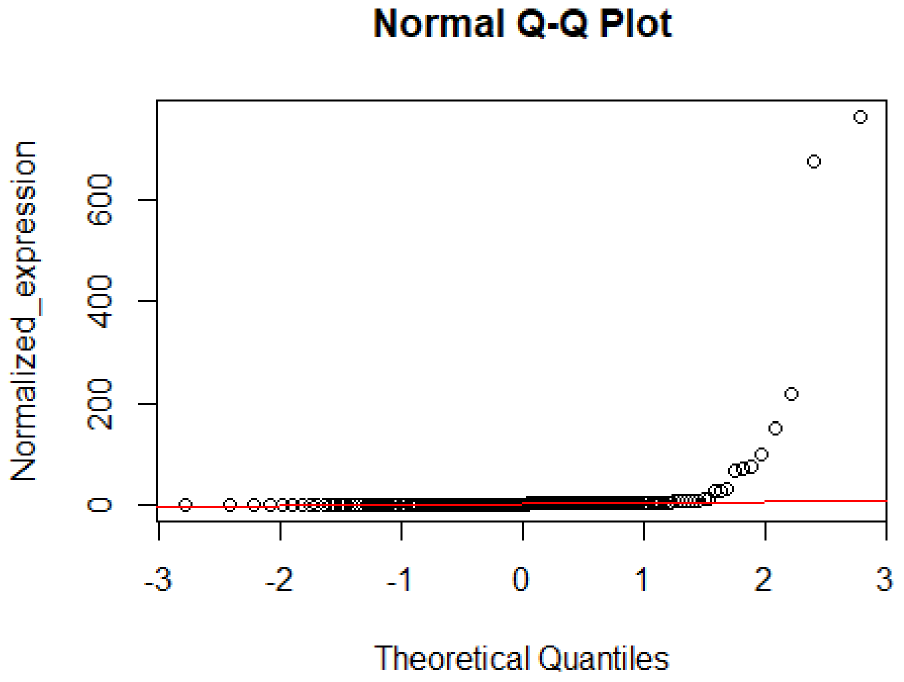

```
plotNormalHistogram(Transformed)
```

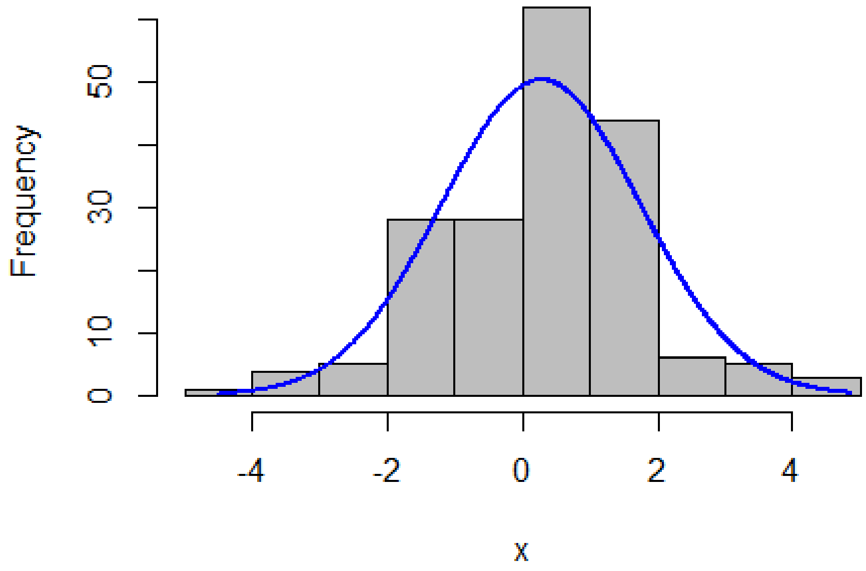


```
qqnorm(Transformed,ylab="Transneg_expression")
qqline(Transformed,
       col="red")
```

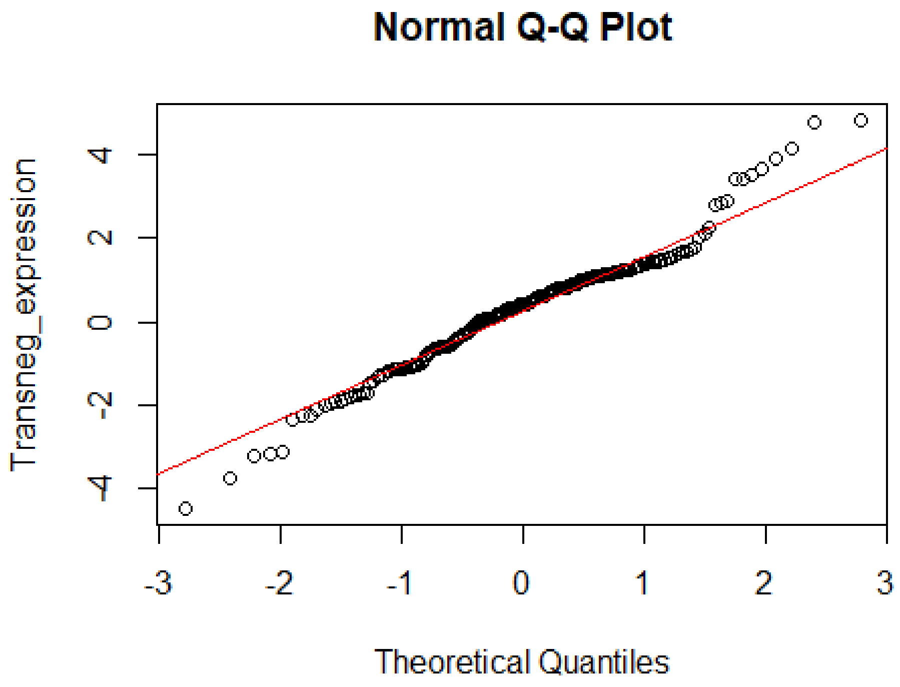


```
#----------------------------------------------------------------

#=======================================================================
=========

ifla<-subset(qpcr, Target=="GmmIV")
ifla

##    Target Sample Irradiation_dose Dpi     Life_stage
Irradiation_conditions
## 1    GmmIV    17A                0   3 Adult (3 dpi)
Hypoxia
## 2    GmmIV    17B                0   3 Adult (3 dpi)
Hypoxia
## 3    GmmIV    18A                0   3 Adult (3 dpi)
Normoxia
## 4    GmmIV    18B                0   3 Adult (3 dpi)
Normoxia
## 5    GmmIV     8A                0   0 Pupae (0 dpi)
Hypoxia
## 6    GmmIV     8B                0   0 Pupae (0 dpi)
Hypoxia
## 7    GmmIV     9A                0   0 Pupae (0 dpi)
Normoxia
## 8    GmmIV     9B                0   0 Pupae (0 dpi)
Normoxia
## 9    GmmIV    11A               70   3 Adult (3 dpi)
```

```
Hypoxia
## 10  GmmIV    11B              70   3 Adult (3 dpi)
Hypoxia
## 11  GmmIV    14A              70   3 Adult (3 dpi)
Normoxia
## 12  GmmIV    14B              70   3 Adult (3 dpi)
Normoxia
## 13  GmmIV     2A              70   0 Pupae (0 dpi)
Hypoxia
## 14  GmmIV     2B              70   0 Pupae (0 dpi)
Hypoxia
## 15  GmmIV     5A              70   0 Pupae (0 dpi)
Normoxia
## 16  GmmIV     5B              70   0 Pupae (0 dpi)
Normoxia
## 17  GmmIV    12A             110   3 Adult (3 dpi)
Hypoxia
## 18  GmmIV    12B             110   3 Adult (3 dpi)
Hypoxia
## 19  GmmIV    15A             110   3 Adult (3 dpi)
Normoxia
## 20  GmmIV    15B             110   3 Adult (3 dpi)
Normoxia
## 21  GmmIV     3A             110   0 Pupae (0 dpi)
Hypoxia
## 22  GmmIV     3B             110   0 Pupae (0 dpi)
Hypoxia
## 23  GmmIV     6A             110   0 Pupae (0 dpi)
Normoxia
## 24  GmmIV     6B             110   0 Pupae (0 dpi)
Normoxia
## 25  GmmIV    13A             150   3 Adult (3 dpi)
Hypoxia
## 26  GmmIV    13B             150   3 Adult (3 dpi)
Hypoxia
## 27  GmmIV    16B             150   3 Adult (3 dpi)
Normoxia
## 28  GmmIV     4A             150   0 Pupae (0 dpi)
Hypoxia
## 29  GmmIV     4B             150   0 Pupae (0 dpi)
Hypoxia
## 30  GmmIV     7A             150   0 Pupae (0 dpi)
Normoxia
## 31  GmmIV     7B             150   0 Pupae (0 dpi)
Normoxia
## 32  GmmIV     1A               0   0 Pupae (0 dpi)
Normoxia
## 33  GmmIV     1B               0   0 Pupae (0 dpi)
Normoxia
## 34  GmmIV     1A               0   0 Pupae (0 dpi)
```

```
Hypoxia
## 35  GmmIV     1b                0   0 Pupae (0 dpi)
Hypoxia
## 36  GmmIV     2a                0   3 Adult (3 dpi)
Hypoxia
## 37  GmmIV     2b                0   3 Adult (3 dpi)
Hypoxia
## 38  GmmIV     3a                0   3 Adult (3 dpi)
Normoxia
## 39  GmmIV     3b                0   3 Adult (3 dpi)
Normoxia
## 40  GmmIV    21A               70   0 Pupae (0 dpi)
Normoxia
## 41  GmmIV    21B               70   0 Pupae (0 dpi)
Normoxia
## 42  GmmIV     3A               70   3 Adult (3 dpi)
Hypoxia
## 43  GmmIV     3B               70   3 Adult (3 dpi)
Hypoxia
## 44  GmmIV     6A               70   3 Adult (3 dpi)
Normoxia
## 45  GmmIV     6B               70   3 Adult (3 dpi)
Normoxia
## 46  GmmIV     2A               70   0 Pupae (0 dpi)
Hypoxia
## 47  GmmIV     2B               70   0 Pupae (0 dpi)
Hypoxia
## 48  GmmIV    22A              110   0 Pupae (0 dpi)
Normoxia
## 49  GmmIV    22B              110   0 Pupae (0 dpi)
Normoxia
## 50  GmmIV     4A              110   3 Adult (3 dpi)
Hypoxia
## 51  GmmIV     4B              110   3 Adult (3 dpi)
Hypoxia
## 52  GmmIV     7A              110   3 Adult (3 dpi)
Normoxia
## 53  GmmIV     7B              110   3 Adult (3 dpi)
Normoxia
## 54  GmmIV     3A              110   0 Pupae (0 dpi)
Hypoxia
## 55  GmmIV     3B              110   0 Pupae (0 dpi)
Hypoxia
## 56  GmmIV    20A              150   0 Pupae (0 dpi)
Hypoxia
## 57  GmmIV    20B              150   0 Pupae (0 dpi)
Hypoxia
## 58  GmmIV    23A              150   0 Pupae (0 dpi)
Normoxia
## 59  GmmIV    23B              150   0 Pupae (0 dpi)
```

```
Normoxia
## 60  GmmIV     5A              150   3 Adult (3 dpi)
Hypoxia
## 61  GmmIV     5B              150   3 Adult (3 dpi)
Hypoxia
## 62  GmmIV     8A              150   3 Adult (3 dpi)
Normoxia
## 63  GmmIV     8B              150   3 Adult (3 dpi)
Normoxia
## 64  GmmIV    16A                0   0 Pupae (0 dpi)
Hypoxia
## 65  GmmIV    16B                0   0 Pupae (0 dpi)
Hypoxia
## 66  GmmIV    17A                0   3 Adult (3 dpi)
Normoxia
## 67  GmmIV    17B                0   3 Adult (3 dpi)
Normoxia
## 68  GmmIV     4a                0   3 Adult (3 dpi)
Hypoxia
## 69  GmmIV     4b                0   3 Adult (3 dpi)
Hypoxia
## 70  GmmIV    10A              110   0 Pupae (0 dpi)
Hypoxia
## 71  GmmIV    10B              110   0 Pupae (0 dpi)
Hypoxia
## 72  GmmIV    13A               70   0 Pupae (0 dpi)
Normoxia
## 73  GmmIV    13B               70   0 Pupae (0 dpi)
Normoxia
## 74  GmmIV    19A              110   3 Adult (3 dpi)
Hypoxia
## 75  GmmIV    19B              110   3 Adult (3 dpi)
Hypoxia
## 76  GmmIV    22A               70   3 Adult (3 dpi)
Normoxia
## 77  GmmIV    22B               70   3 Adult (3 dpi)
Normoxia
## 78  GmmIV    11A              150   0 Pupae (0 dpi)
Hypoxia
## 79  GmmIV    14A              110   0 Pupae (0 dpi)
Normoxia
## 80  GmmIV    14B              110   0 Pupae (0 dpi)
Normoxia
## 81  GmmIV    20A              150   3 Adult (3 dpi)
Hypoxia
## 82  GmmIV    20B              150   3 Adult (3 dpi)
Hypoxia
## 83  GmmIV     4A              110   3 Adult (3 dpi)
Normoxia
## 84  GmmIV     4B              110   3 Adult (3 dpi)
```

```
Normoxia
## 85  GmmIV    12A             190   0 Pupae (0 dpi)
Hypoxia
## 86  GmmIV    12B             190   0 Pupae (0 dpi)
Hypoxia
## 87  GmmIV    15A             150   0 Pupae (0 dpi)
Normoxia
## 88  GmmIV    15B             150   0 Pupae (0 dpi)
Normoxia
## 89  GmmIV    21A             190   3 Adult (3 dpi)
Hypoxia
## 90  GmmIV    21B             190   3 Adult (3 dpi)
Hypoxia
## 91  GmmIV     5A             150   3 Adult (3 dpi)
Normoxia
## 92  GmmIV     5B             150   3 Adult (3 dpi)
Normoxia
##    Replicate  Expression Mean.Cq Normalized_expression Transformed
## 1          1   0.1090300   22.03             0.4604599 -0.80639449
## 2          1   0.1252000   22.18             0.5287497 -0.65798208
## 3          1   0.1346500   21.74             0.5686593 -0.58070931
## 4          1   0.1253500   21.86             0.5293832 -0.65670601
## 5          1   1.0128188   20.06             3.0964529  1.06872315
## 6          1   0.9783009   20.14             2.9909226  1.03769990
## 7          1   4.2774600   20.65            13.0773182  2.26699785
## 8          1   0.2131900   24.97             0.6517778 -0.43734511
## 9          1   0.1312300   21.22             0.5542158 -0.60796567
## 10         1   0.0281800   23.20             0.1190109 -2.37204012
## 11         1  51.4884900   23.08           217.4482759  4.16199580
## 12         1  35.4320700   23.73           149.6381528  3.93967708
## 13         1   0.3535200   23.98             1.0808034  0.07740358
## 14         1   0.5363300   23.34             1.6397016  0.48248615
## 15         1   1.1104700   21.11             3.3949983  1.15055481
## 16         1   0.7600200   21.47             2.3235807  0.80854586
## 17         1   0.1811900   21.22             0.7652089 -0.27121916
## 18         1   0.1300200   21.37             0.5491057 -0.61779662
## 19         1   0.1700800   20.48             0.7182887 -0.33641872
## 20         1   0.1377400   20.66             0.5817091 -0.55672992
## 21         1   1.0421000   21.21             3.1859733  1.09414157
## 22         1   0.8134100   21.31             2.4868079  0.87073579
## 23         1   0.3025000   22.33             0.9248219 -0.07846029
## 24         1   0.1771300   22.62             0.5415329 -0.63255198
## 25         1   0.1016900   21.38             0.4294613 -0.88197175
## 26         1   0.0811200   21.68             0.3425893 -1.13070376
## 27         1   0.0890500   22.69             0.3760796 -1.02737202
## 28         1   0.3971000   21.14             1.2140389  0.19208399
## 29         1   0.2948100   21.36             0.9013116 -0.10444596
## 30         1   3.0791500   19.95             9.4137699  2.00858578
## 31         1   0.9467200   20.14             2.8943716  1.00824280
## 32         2  41.6958900   18.76             1.5284571  0.41538497
```

```
## 33           2  44.1936500   18.90             1.6200182  0.47098498
## 34           2   4.9830300   20.88             0.1826642 -1.85317370
## 35           2   4.0377800   21.10             0.1480140 -2.10513769
## 36           2   2.4963200   21.57             1.1200237  0.11270985
## 37           2   2.4351600   21.58             1.0925830  0.08815380
## 38           2   2.4411800   21.48             1.0952840  0.09060080
## 39           2   1.7750200   21.63             0.7963981 -0.23026726
## 40           2  77.4999800   18.05             2.8409370  0.99147185
## 41           2  89.1371500   18.02             3.2675238  1.11662237
## 42           2   0.3540300   21.12             0.1588426 -2.01996763
## 43           2   0.2905300   21.09             0.1303521 -2.25993610
## 44           2   0.7990400   21.06             0.3585052 -1.08027279
## 45           2   0.5569800   21.31             0.2499002 -1.48744234
## 46           2  85.4123400   18.18             3.1309824  1.07862208
## 47           2  51.9678200   18.22             1.9049979  0.62415220
## 48           2  77.0431500   18.06             2.8241909  0.98614442
## 49           2  70.0133600   18.17             2.5664980  0.89948632
## 50           2   1.5843400   20.05             0.7108457 -0.34719100
## 51           2   1.4308200   19.84             0.6419659 -0.45318904
## 52           2   0.7566700   25.64             0.3394951 -1.14080706
## 53           2   0.4437700   25.93             0.1991063 -1.75145145
## 54           2  70.2479300   17.60             2.5750967  0.90252972
## 55           2  54.1456100   17.67             1.9848298  0.66256323
## 56           2  63.0303700   19.38             2.3105207  0.80336367
## 57           2  55.6890600   19.66             2.0414084  0.68877095
## 58           2 131.2552700   17.80             4.8114587  1.45381433
## 59           2 103.3295600   18.06             3.7877787  1.24690704
## 60           2  14.0394200   23.05             6.2990654  1.68097579
## 61           2   8.8575800   23.14             3.9741297  1.28884390
## 62           2   1.0936700   21.94             0.4906968 -0.73788318
## 63           2   0.7212700   21.90             0.3236122 -1.19431485
## 64           3  14.4368800   17.24             1.3286184  0.28014077
## 65           3  13.5849800   17.28             1.2502185  0.22084321
## 66           3   9.0625900   17.96            75.1645517  3.50769831
## 67           3   8.3693500   18.03            69.4148627  3.45582728
## 68           3  11.9261400   19.77            98.9146554  3.68353730
## 69           3   3.4096000   21.27            28.2790081  2.84097988
## 70           3   5.3506800   18.51             0.4924202 -0.73411916
## 71           3   5.9361300   18.40             0.5462989 -0.62323942
## 72           3   5.9905200   17.58             0.5513044 -0.61355458
## 73           3   5.4069100   17.79             0.4975950 -0.72290345
## 74           3   0.5584300   20.94             4.6315833  1.42118995
## 75           3   0.4598800   21.13             3.8142158  1.25299301
## 76           3   0.2825900   19.52             2.3437837  0.81649964
## 77           3   0.2831800   19.69             2.3486771  0.81841480
## 78           3  11.0635400   18.09             1.0181717  0.01799234
## 79           3  15.4973400   18.25             1.4262119  0.34879382
## 80           3   7.1197500   18.13             0.6552268 -0.43183803
## 81           3   0.2620600   19.74             2.1735092  0.74697251
## 82           3   0.2224400   20.03             1.8449034  0.59405061
```

```
## 83          3   0.3690400   19.24            3.0607946  1.05837233
## 84          3   0.2667800   19.50            2.2126565  0.76347523
## 85          3   7.8034500   17.96            0.7181473 -0.33662223
## 86          3   3.7812600   18.02            0.3479873 -1.11331580
## 87          3  13.1375900   17.86            1.2090454  0.18804067
## 88          3  13.0945400   17.75            1.2050835  0.18481962
## 89          3   0.1813300   19.87            1.5039396  0.39987341
## 90          3   0.1838700   20.09            1.5250062  0.41321829
## 91          3   0.8061700   18.81            6.6863233  1.73046170
## 92          3   0.4974200   19.22            4.1255702  1.32136144

#select the model

anova(with(ifla,lm(Transformed ~ Irradiation_dose)))

## Analysis of Variance Table
##
## Response: Transformed
##                  Df  Sum Sq Mean Sq F value Pr(>F)
## Irradiation_dose  4   3.875 0.96881  0.5443 0.7036
## Residuals        87 154.840 1.77977

anova(with(ifla,lm(Transformed ~ Life_stage)))

## Analysis of Variance Table
##
## Response: Transformed
##            Df  Sum Sq Mean Sq F value Pr(>F)
## Life_stage  1   0.134 0.13373  0.0759 0.7836
## Residuals  90 158.582 1.76202

anova(with(ifla,lm(Transformed ~ Irradiation_conditions)))

## Analysis of Variance Table
##
## Response: Transformed
##                        Df  Sum Sq Mean Sq F value Pr(>F)
## Irradiation_conditions  1   3.848  3.8478  2.2361 0.1383
## Residuals              90 154.867  1.7207

anova(with(ifla,lm(Transformed ~ Irradiation_dose +Life_stage)))

## Analysis of Variance Table
##
## Response: Transformed
##                  Df  Sum Sq Mean Sq F value Pr(>F)
## Irradiation_dose  4   3.875 0.96881  0.5387 0.7077
## Life_stage        1   0.173 0.17339  0.0964 0.7569
## Residuals        86 154.667 1.79845

anova(with(ifla,lm(Transformed ~
Irradiation_dose+Irradiation_conditions)))
```

```
## Analysis of Variance Table
##
## Response: Transformed
##                          Df  Sum Sq Mean Sq F value Pr(>F)
## Irradiation_dose          4   3.875  0.9688  0.5509 0.6989
## Irradiation_conditions    1   3.591  3.5907  2.0416 0.1567
## Residuals                86 151.249  1.7587

anova(with(ifla,lm(Transformed ~ Irradiation_dose *Life_stage)))

## Analysis of Variance Table
##
## Response: Transformed
##                              Df  Sum Sq Mean Sq F value Pr(>F)
## Irradiation_dose              4   3.875 0.96881  0.5403 0.7065
## Life_stage                    1   0.173 0.17339  0.0967 0.7566
## Irradiation_dose:Life_stage   4   7.638 1.90948  1.0649 0.3792
## Residuals                    82 147.029 1.79303

anova(with(ifla,lm(Transformed ~ Irradiation_dose
*Irradiation_conditions)))

## Analysis of Variance Table
##
## Response: Transformed
##                                          Df  Sum Sq Mean Sq F value
Pr(>F)
## Irradiation_dose                          4   3.875  0.9688  0.5645
0.6891
## Irradiation_conditions                    1   3.591  3.5907  2.0921
0.1518
## Irradiation_dose:Irradiation_conditions   3   8.798  2.9327  1.7087
0.1715
## Residuals                                83 142.451  1.7163

#-----------------------------------------------------------------------
-----------------------------------------


mod1 <- lm(Transformed ~ Irradiation_dose + Irradiation_conditions,
data = ifla)
mod2 <- lm(Transformed ~ Irradiation_dose * Irradiation_conditions,
data = ifla)
mod3 <- lm(Transformed ~ Irradiation_dose + Life_stage +
Irradiation_conditions, data = ifla)
mod4 <- lm(Transformed ~ Irradiation_dose * Life_stage *
Irradiation_conditions, data = ifla)
mod5 <- lm(Transformed ~ Irradiation_dose + Life_stage, data = ifla)
mod6 <- lm(Transformed ~ Irradiation_dose*Life_stage, data = ifla)
mod7 <- lm(Transformed ~ Irradiation_dose, data = ifla)
mod8 <- lm(Transformed ~ Irradiation_conditions, data = ifla)
```

```r
mod9 <- lm(Transformed ~  Life_stage, data = ifla)
mod10<- lm(Transformed ~ Irradiation_dose*Life_stage, data = ifla)

AICc(mod1,mod2,mod3,mod4,mod5,mod6,mod7,mod8,mod9,mod10)

##       df     AICc
## mod1   7 322.1550
## mod2  10 324.0242
## mod3   8 324.4492
## mod4  19 334.6839
## mod5   7 324.2105
## mod6  11 329.5179
## mod7   6 321.9685
## mod8   3 315.2693
## mod9   3 317.4496
## mod10 11 329.5179

anova(mod8, mod9)

## Analysis of Variance Table
##
## Model 1: Transformed ~ Irradiation_conditions
## Model 2: Transformed ~ Life_stage
##   Res.Df    RSS Df Sum of Sq F Pr(>F)
## 1     90 154.87
## 2     90 158.58  0   -3.7141

##no difference, choose either model but went for the lowest

summary(mod8)

##
## Call:
## lm(formula = Transformed ~ Irradiation_conditions, data = ifla)
##
## Residuals:
##     Min      1Q  Median      3Q     Max
## -2.4854 -0.9284 -0.0130  0.6477  3.6395
##
## Coefficients:
##                                  Estimate Std. Error t value Pr(>|t|)
## (Intercept)                        0.1134     0.1913   0.593    0.555
## Irradiation_conditionsNormoxia   0.4091     0.2736   1.495    0.138
##
## Residual standard error: 1.312 on 90 degrees of freedom
## Multiple R-squared:  0.02424,    Adjusted R-squared:  0.0134
## F-statistic: 2.236 on 1 and 90 DF,  p-value: 0.1383

#-----------------------------------------------------------------------
--------
```

```
#===========================================================================
==============
###Irradiation dose

ifla$Irradiation_dose=as.factor(ifla$Irradiation_dose)
label_parse <- function(breaks) {parse(text = breaks)}

Fig1a<-ggplot(ifla,aes(x=Irradiation_dose,y=Transformed,
fill=Irradiation_dose)) +
  geom_boxplot()+
geom_jitter(width=0.1,alpha=0.2)+scale_y_continuous(labels =
label_parse)
Fig1a
```

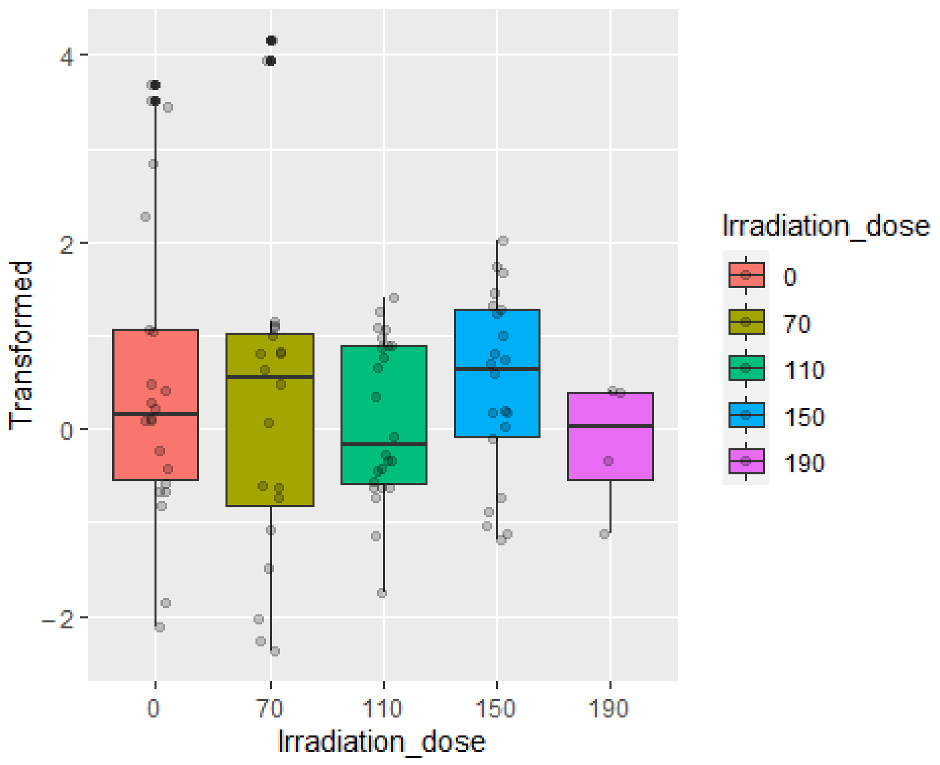


```
tiff("Fig 1a.tiff", width = 4, height = 4, units = 'in', res = 300)
plot(Fig1a+theme_tufte() + theme(axis.line = element_line(size = 1,
colour = "black")) + theme(axis.text.x = element_blank())
+theme(legend.title = element_text(face = "bold")) + theme(legend.text
= element_text(face = "plain"))) + xlab(expression(bold("Irradiation
doses (Gy)"))) + ylab(expression (bold("Transformed relative  GmmIV
density")))
dev.off()

## png
##   2
```

```
###Irradiation status
#ifla$Irradiation_conditions=as.factor(ifla$Irradiation_conditions)

label_parse <- function(breaks) {parse(text = breaks)}

Fig1b<-ggplot(ifla,aes(x=Irradiation_conditions,y=Transformed,
fill=Irradiation_conditions)) +
  geom_boxplot() + geom_jitter(width=0.1,alpha=0.2)+
scale_y_continuous(labels = label_parse)
Fig1b
```

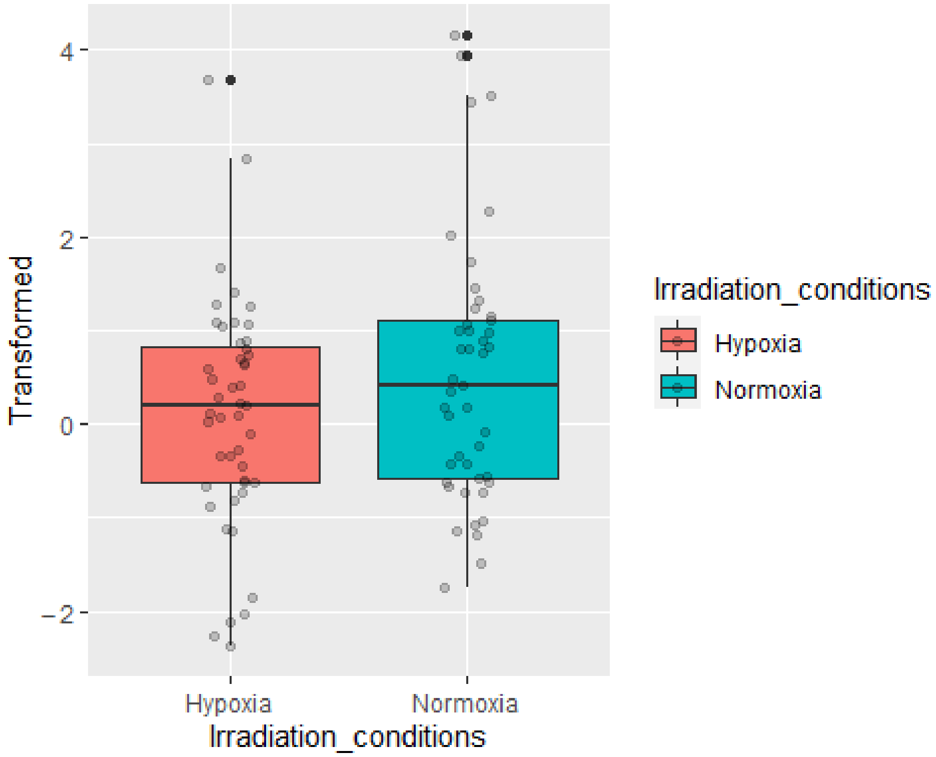


```
tiff("Fig 1b.tiff", width = 4, height = 4, units = 'in', res = 300)
plot(Fig1b+theme_tufte() + theme(axis.line = element_line(size = 1,
colour = "black")) + theme(axis.text.x = element_blank())
+theme(legend.title = element_text(face = "bold")) + theme(legend.text
= element_text(face = "plain"))) + xlab(expression(bold("Irradiation
status "))) + ylab(expression (bold("Transformed  relative GmmIV
density")))
dev.off()

## png
##   2

###Days_post.irradiation
#ifla$Life_stage=as.factor(ifla$Life_stage)

label_parse <- function(breaks) {parse(text = breaks)}
```

```
Fig1c<-ggplot(ifla,aes(x=Life_stage,y=Transformed, fill=Life_stage)) +
  geom_boxplot() + geom_jitter(width=0.1,alpha=0.2)+
scale_y_continuous(labels = label_parse)
Fig1c
```

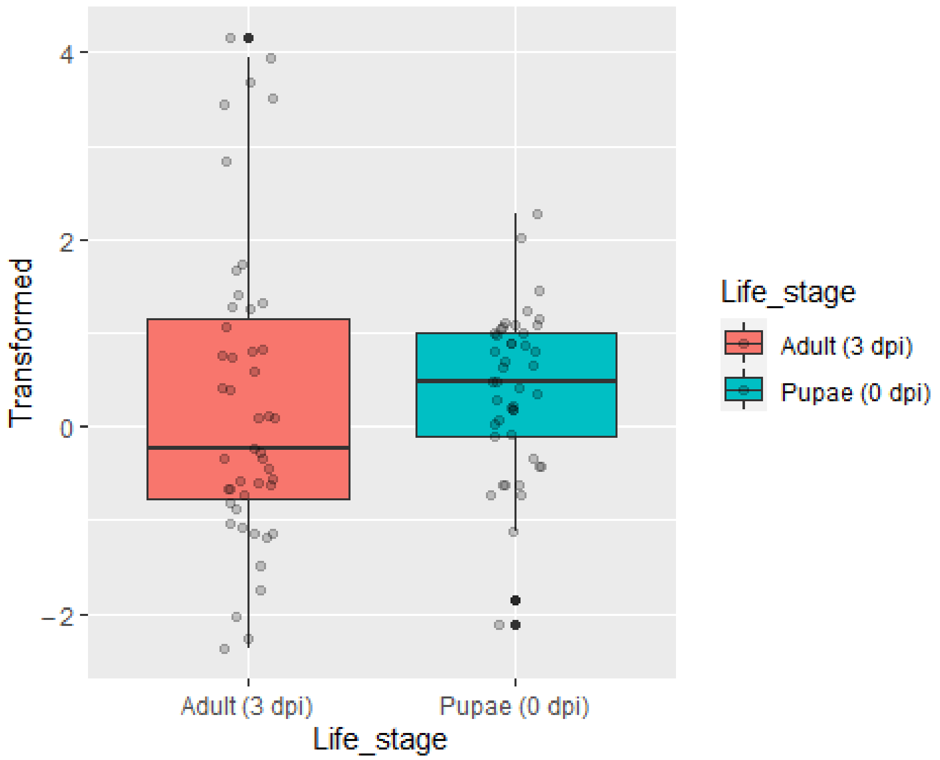


```
tiff("Fig 1c.tiff", width = 4, height = 4, units = 'in', res = 300)
plot(Fig1c+theme_tufte() + theme(axis.line = element_line(size = 1,
colour = "black")) + theme(axis.text.x = element_blank())
+theme(legend.title = element_text(face = "bold")) + theme(legend.text
= element_text(face = "plain"))) + xlab(expression(bold("Days post
irradiation"))) + ylab(expression (bold("Transformed  relative GmmIV
density")))
dev.off()

## png
##   2

###Irradiation dose according to air status

label_parse <- function(breaks) {parse(text = breaks)}


Fig2a<-ggplot(ifla, aes(x=Irradiation_dose,y=Transformed,
colour=Irradiation_conditions)) +
  geom_boxplot(position=position_dodge(0.8))+
  geom_jitter(position=position_dodge(0.8))+ scale_y_continuous(labels
```

```
= label_parse)

Fig2a
```

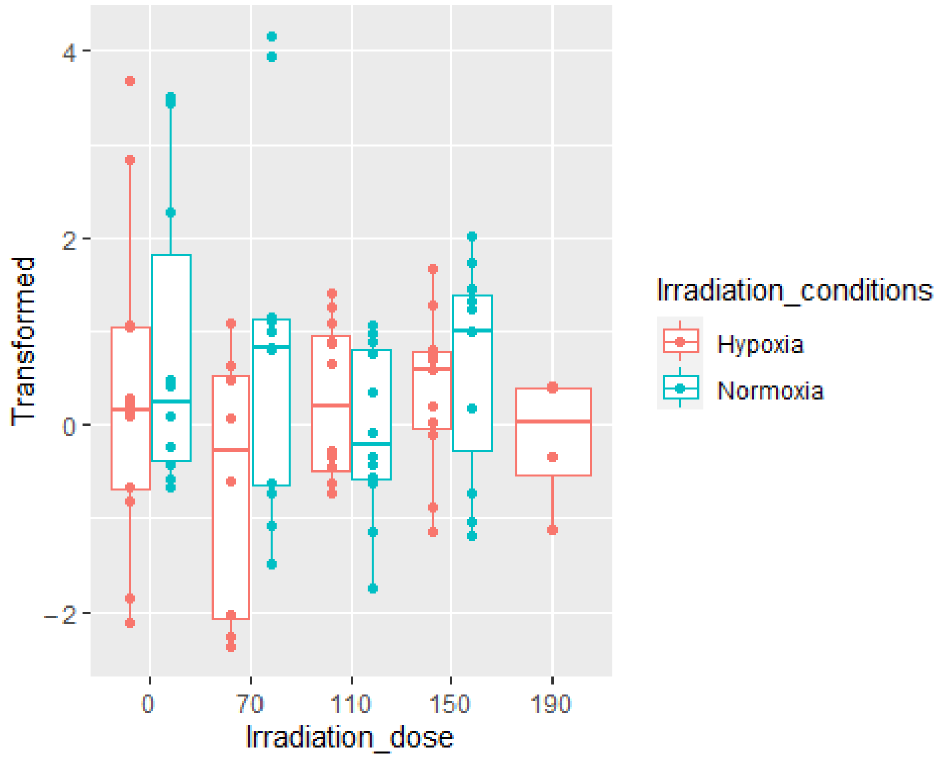


```
tiff("Fig 2a.tiff", width = 7, height = 4, units = 'in',compression =
'lzw',res = 300)
plot(Fig2a+theme_tufte() + theme(axis.line = element_line(size = 1,
colour = "black")) + theme(legend.title= element_text(face = "bold")) +
theme(legend.text = element_text(face = "plain"))) + theme(axis.text.x
= element_text(colour = "black")) + theme(axis.text.y =
element_text(colour = "black")) + xlab(expression(bold("Irradiation
dose (Gy)"))) + ylab(expression(bold("GmmIV_ ")~bold("Transformed
normalized density")))
dev.off()

## png
##   2

###Irradiation dose according to pupae stage/ days post irradiation
label_parse <- function(breaks) {parse(text = breaks)}

Fig2b<-ggplot(ifla, aes(x=Irradiation_dose,y=Transformed,
colour=Life_stage)) +
  geom_boxplot(position=position_dodge(0.8))+
  geom_jitter(position=position_dodge(0.8))+ scale_y_continuous(labels
= label_parse)
Fig2b
```

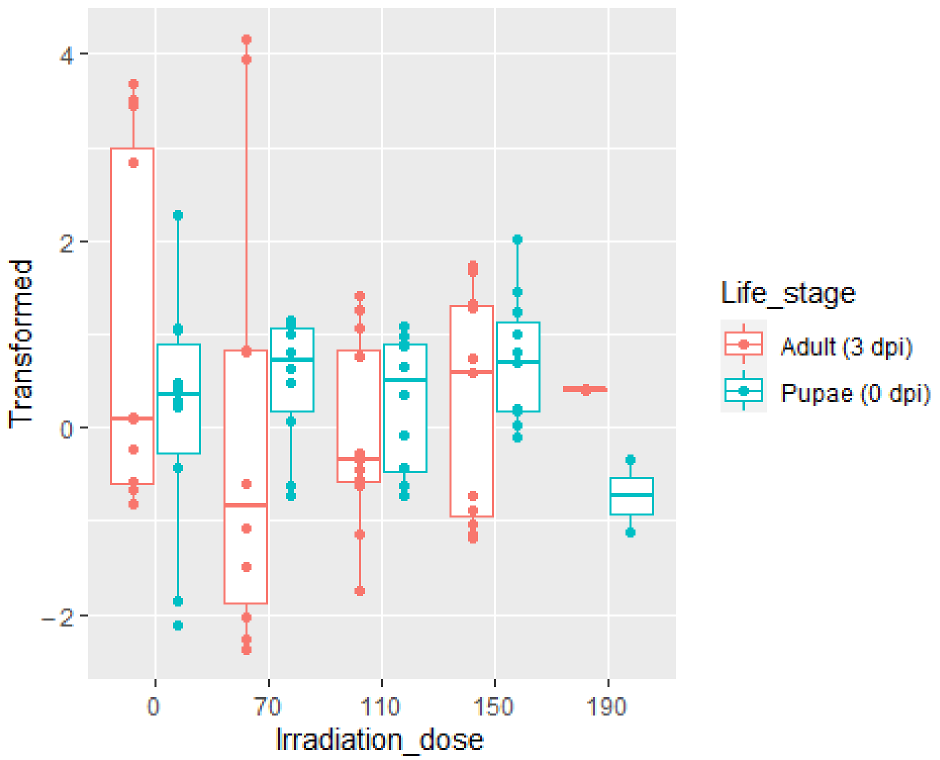


```
tiff("Fig 2b.tiff", width = 7, height = 4, units = 'in',compression =
'lzw',res = 300)
plot(Fig2b+theme_tufte() + theme(axis.line = element_line(size = 1,
colour = "black")) + theme(legend.title= element_text(face = "bold")) +
theme(legend.text = element_text(face = "plain"))) + theme(axis.text.x
= element_text(colour = "black")) + theme(axis.text.y =
element_text(colour = "black")) + xlab(expression(bold("Irradiation
dose (Gy)"))) + ylab(expression(bold("GmmIV_ ")~bold("Transformed
normalized density")))
dev.off()

## png
##   2

# #Statistics iflaevirus  for subsets


#------------------------------------------------------------------

#iflaevirus-Normoxia
#--------------------------------------------
#iflaNorm<- read.csv("iflaevirus_alldata_normalized.csv")
iflaNorm<- subset(ifla, Irradiation_conditions=="Normoxia")
iflaNorm

##    Target Sample Irradiation_dose Dpi    Life_stage
Irradiation_conditions
```

```
## 3   GmmIV    18A                 0   3 Adult (3 dpi)
Normoxia
## 4   GmmIV    18B                 0   3 Adult (3 dpi)
Normoxia
## 7   GmmIV     9A                 0   0 Pupae (0 dpi)
Normoxia
## 8   GmmIV     9B                 0   0 Pupae (0 dpi)
Normoxia
## 11  GmmIV    14A                70   3 Adult (3 dpi)
Normoxia
## 12  GmmIV    14B                70   3 Adult (3 dpi)
Normoxia
## 15  GmmIV     5A                70   0 Pupae (0 dpi)
Normoxia
## 16  GmmIV     5B                70   0 Pupae (0 dpi)
Normoxia
## 19  GmmIV    15A               110   3 Adult (3 dpi)
Normoxia
## 20  GmmIV    15B               110   3 Adult (3 dpi)
Normoxia
## 23  GmmIV     6A               110   0 Pupae (0 dpi)
Normoxia
## 24  GmmIV     6B               110   0 Pupae (0 dpi)
Normoxia
## 27  GmmIV    16B               150   3 Adult (3 dpi)
Normoxia
## 30  GmmIV     7A               150   0 Pupae (0 dpi)
Normoxia
## 31  GmmIV     7B               150   0 Pupae (0 dpi)
Normoxia
## 32  GmmIV     1A                 0   0 Pupae (0 dpi)
Normoxia
## 33  GmmIV     1B                 0   0 Pupae (0 dpi)
Normoxia
## 38  GmmIV     3a                 0   3 Adult (3 dpi)
Normoxia
## 39  GmmIV     3b                 0   3 Adult (3 dpi)
Normoxia
## 40  GmmIV    21A                70   0 Pupae (0 dpi)
Normoxia
## 41  GmmIV    21B                70   0 Pupae (0 dpi)
Normoxia
## 44  GmmIV     6A                70   3 Adult (3 dpi)
Normoxia
## 45  GmmIV     6B                70   3 Adult (3 dpi)
Normoxia
## 48  GmmIV    22A               110   0 Pupae (0 dpi)
Normoxia
## 49  GmmIV    22B               110   0 Pupae (0 dpi)
Normoxia
```

```
## 52  GmmIV     7A              110   3 Adult (3 dpi)
Normoxia
## 53  GmmIV     7B              110   3 Adult (3 dpi)
Normoxia
## 58  GmmIV    23A              150   0 Pupae (0 dpi)
Normoxia
## 59  GmmIV    23B              150   0 Pupae (0 dpi)
Normoxia
## 62  GmmIV     8A              150   3 Adult (3 dpi)
Normoxia
## 63  GmmIV     8B              150   3 Adult (3 dpi)
Normoxia
## 66  GmmIV    17A                0   3 Adult (3 dpi)
Normoxia
## 67  GmmIV    17B                0   3 Adult (3 dpi)
Normoxia
## 72  GmmIV    13A               70   0 Pupae (0 dpi)
Normoxia
## 73  GmmIV    13B               70   0 Pupae (0 dpi)
Normoxia
## 76  GmmIV    22A               70   3 Adult (3 dpi)
Normoxia
## 77  GmmIV    22B               70   3 Adult (3 dpi)
Normoxia
## 79  GmmIV    14A              110   0 Pupae (0 dpi)
Normoxia
## 80  GmmIV    14B              110   0 Pupae (0 dpi)
Normoxia
## 83  GmmIV     4A              110   3 Adult (3 dpi)
Normoxia
## 84  GmmIV     4B              110   3 Adult (3 dpi)
Normoxia
## 87  GmmIV    15A              150   0 Pupae (0 dpi)
Normoxia
## 88  GmmIV    15B              150   0 Pupae (0 dpi)
Normoxia
## 91  GmmIV     5A              150   3 Adult (3 dpi)
Normoxia
## 92  GmmIV     5B              150   3 Adult (3 dpi)
Normoxia
##    Replicate Expression Mean.Cq Normalized_expression Transformed
## 3          1    0.13465   21.74             0.5686593 -0.58070931
## 4          1    0.12535   21.86             0.5293832 -0.65670601
## 7          1    4.27746   20.65            13.0773182  2.26699785
## 8          1    0.21319   24.97             0.6517778 -0.43734511
## 11         1   51.48849   23.08           217.4482759  4.16199580
## 12         1   35.43207   23.73           149.6381528  3.93967708
## 15         1    1.11047   21.11             3.3949983  1.15055481
## 16         1    0.76002   21.47             2.3235807  0.80854586
## 19         1    0.17008   20.48             0.7182887 -0.33641872
```

```
## 20          1    0.13774   20.66             0.5817091 -0.55672992
## 23          1    0.30250   22.33             0.9248219 -0.07846029
## 24          1    0.17713   22.62             0.5415329 -0.63255198
## 27          1    0.08905   22.69             0.3760796 -1.02737202
## 30          1    3.07915   19.95             9.4137699  2.00858578
## 31          1    0.94672   20.14             2.8943716  1.00824280
## 32          2   41.69589   18.76             1.5284571  0.41538497
## 33          2   44.19365   18.90             1.6200182  0.47098498
## 38          2    2.44118   21.48             1.0952840  0.09060080
## 39          2    1.77502   21.63             0.7963981 -0.23026726
## 40          2   77.49998   18.05             2.8409370  0.99147185
## 41          2   89.13715   18.02             3.2675238  1.11662237
## 44          2    0.79904   21.06             0.3585052 -1.08027279
## 45          2    0.55698   21.31             0.2499002 -1.48744234
## 48          2   77.04315   18.06             2.8241909  0.98614442
## 49          2   70.01336   18.17             2.5664980  0.89948632
## 52          2    0.75667   25.64             0.3394951 -1.14080706
## 53          2    0.44377   25.93             0.1991063 -1.75145145
## 58          2  131.25527   17.80             4.8114587  1.45381433
## 59          2  103.32956   18.06             3.7877787  1.24690704
## 62          2    1.09367   21.94             0.4906968 -0.73788318
## 63          2    0.72127   21.90             0.3236122 -1.19431485
## 66          3    9.06259   17.96            75.1645517  3.50769831
## 67          3    8.36935   18.03            69.4148627  3.45582728
## 72          3    5.99052   17.58             0.5513044 -0.61355458
## 73          3    5.40691   17.79             0.4975950 -0.72290345
## 76          3    0.28259   19.52             2.3437837  0.81649964
## 77          3    0.28318   19.69             2.3486771  0.81841480
## 79          3   15.49734   18.25             1.4262119  0.34879382
## 80          3    7.11975   18.13             0.6552268 -0.43183803
## 83          3    0.36904   19.24             3.0607946  1.05837233
## 84          3    0.26678   19.50             2.2126565  0.76347523
## 87          3   13.13759   17.86             1.2090454  0.18804067
## 88          3   13.09454   17.75             1.2050835  0.18481962
## 91          3    0.80617   18.81             6.6863233  1.73046170
## 92          3    0.49742   19.22             4.1255702  1.32136144

#-------------------------------------------------------------------
anova(with(iflaNorm,lm(Transformed~ Life_stage*Irradiation_dose)))

## Analysis of Variance Table
##
## Response: Transformed
##                             Df Sum Sq Mean Sq F value Pr(>F)
## Life_stage                   1  0.114 0.11429  0.0563 0.8138
## Irradiation_dose             3  6.380 2.12671  1.0473 0.3831
## Life_stage:Irradiation_dose  3  5.099 1.69966  0.8370 0.4822
## Residuals                   37 75.135 2.03067

anova(with(iflaNorm,lm(Transformed~ Life_stage +Irradiation_dose)))
```

```
## Analysis of Variance Table
##
## Response: Transformed
##                   Df Sum Sq Mean Sq F value Pr(>F)
## Life_stage         1  0.114 0.11429  0.0570 0.8126
## Irradiation_dose   3  6.380 2.12671  1.0603 0.3767
## Residuals         40 80.234 2.00585

anova(with(iflaNorm,lm(Transformed~ Life_stage)))

## Analysis of Variance Table
##
## Response: Transformed
##             Df Sum Sq Mean Sq F value Pr(>F)
## Life_stage   1  0.114 0.11429  0.0567 0.8129
## Residuals   43 86.614 2.01428

anova(with(iflaNorm,lm(Transformed~ Irradiation_dose)))

## Analysis of Variance Table
##
## Response: Transformed
##                   Df Sum Sq Mean Sq F value Pr(>F)
## Irradiation_dose   3  6.313  2.1043  1.0729 0.3712
## Residuals         41 80.415  1.9613

mod1 <- lm(Transformed ~ Irradiation_dose, data = iflaNorm)
mod5 <- lm(Transformed~ Irradiation_dose+Life_stage, data = iflaNorm)
mod6 <- lm(Transformed ~ Irradiation_dose*Life_stage, data = iflaNorm)
mod11 <- lm(Transformed ~Life_stage, data = iflaNorm)


AICc(mod1, mod5, mod6, mod11)

##        df     AICc
## mod1    5 165.3674
## mod5    6 167.9377
## mod6    9 173.9153
## mod11   3 163.7558

anova(mod11, mod1)

## Analysis of Variance Table
##
## Model 1: Transformed ~ Life_stage
## Model 2: Transformed ~ Irradiation_dose
##   Res.Df    RSS Df Sum of Sq      F Pr(>F)
## 1     43 86.614
## 2     41 80.415  2    6.1985 1.5802 0.2182

summary(mod11)
```

```
##
## Call:
## lm(formula = Transformed ~ Life_stage, data = iflaNorm)
##
## Residuals:
##     Min      1Q  Median      3Q     Max
## -2.2247 -1.0539 -0.1586  0.5765  3.6888
##
## Coefficients:
##                          Estimate Std. Error t value Pr(>|t|)
## (Intercept)                0.4732     0.2959   1.599    0.117
## Life_stagePupae (0 dpi)    0.1008     0.4232   0.238    0.813
##
## Residual standard error: 1.419 on 43 degrees of freedom
## Multiple R-squared:  0.001318,   Adjusted R-squared:  -0.02191
## F-statistic: 0.05674 on 1 and 43 DF,  p-value: 0.8129

#--------------------------------------------------------------

#FIGURE_normoxia
#iflaNorm$Life_stage=as.factor(iflaNorm$Life_stage)

label_parse <- function(breaks) {parse(text = breaks)}

FigS2A<-ggplot(iflaNorm, aes(x=Irradiation_dose,y=Transformed, colour=
Life_stage
)) +geom_boxplot(position=position_dodge(0.8))+
  geom_jitter(position=position_dodge(0.8))+
  scale_y_continuous(labels = label_parse)

FigS2A
```

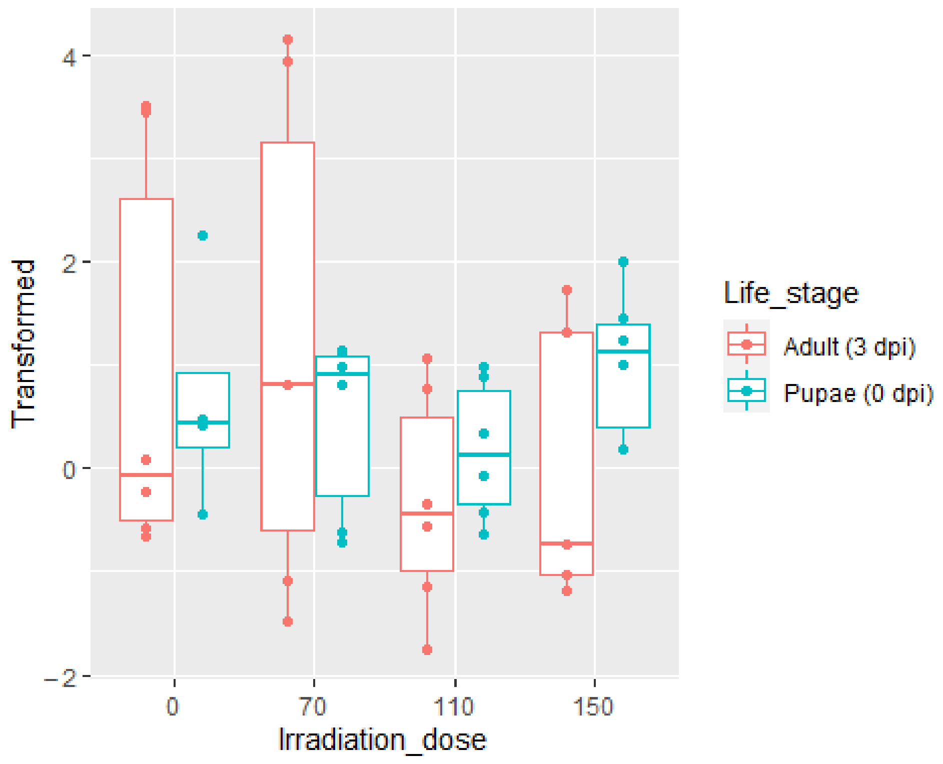


```
tiff("Fig S2A.tiff", width = 7, height = 4, units = 'in',compression =
'lzw',res = 300)
plot(FigS2A+theme_tufte() + theme(axis.line = element_line(size = 1,
colour = "black")) + theme(legend.title= element_text(face = "bold")) +
theme(legend.text = element_text(face = "plain"))) + theme(axis.text.x
= element_text(colour = "black")) + theme(axis.text.y =
element_text(colour = "black")) + xlab(expression(bold("Irradiation
dose (Gy)"))) + ylab(expression(bold("GmmIV_ ")~bold("Normoxia
transformed relative density")))
dev.off()

## png
##   2

#-------------------------------------------------------
#iflaevirus-Hypoxia
#------------------------------------------------
#iflaHypox <- read.csv("iflaevirus_alldata_normalized.csv")
iflaHypox <- subset(ifla , Irradiation_conditions=="Hypoxia")

#----------------------------------------------------------------------
---
anova(with(iflaHypox,lm(Transformed~  Life_stage+Irradiation_dose)))

## Analysis of Variance Table
##
## Response: Transformed
```

```
##                   Df Sum Sq Mean Sq F value Pr(>F)
## Life_stage         1  0.033 0.03318  0.0219 0.8830
## Irradiation_dose   4  6.117 1.52936  1.0115 0.4127
## Residuals         41 61.989 1.51192

anova(with(iflaHypox,lm(Transformed~  Life_stage*Irradiation_dose)))

## Analysis of Variance Table
##
## Response: Transformed
##                              Df Sum Sq Mean Sq F value  Pr(>F)
## Life_stage                    1  0.033  0.0332  0.0269 0.87064
## Irradiation_dose              4  6.117  1.5294  1.2396 0.31107
## Life_stage:Irradiation_dose   4 16.340  4.0849  3.3110 0.02046 *
## Residuals                    37 45.649  1.2338
## ---
## Signif. codes:  0 '***' 0.001 '**' 0.01 '*' 0.05 '.' 0.1 ' ' 1

anova(with(iflaHypox,lm(Transformed~  Life_stage)))

## Analysis of Variance Table
##
## Response: Transformed
##             Df Sum Sq Mean Sq F value Pr(>F)
## Life_stage   1  0.033 0.03318  0.0219  0.883
## Residuals   45 68.106 1.51347

anova(with(iflaHypox,lm(Transformed~  Irradiation_dose)))

## Analysis of Variance Table
##
## Response: Transformed
##                   Df Sum Sq Mean Sq F value Pr(>F)
## Irradiation_dose   4  6.103  1.5258   1.033 0.4015
## Residuals         42 62.036  1.4770

mod1 <- lm(Transformed ~ Irradiation_dose, data = iflaHypox)
mod5 <- lm(Transformed~ Irradiation_dose+Life_stage, data = iflaHypox)
mod6 <- lm(Transformed ~ Irradiation_dose*Life_stage, data = iflaHypox)
mod11 <- lm(Transformed ~Life_stage, data = iflaHypox)

AICc(mod1, mod5, mod6, mod11)

##       df     AICc
## mod1   6 160.5258
## mod5   7 163.2617
## mod6  11 161.5522
## mod11  3 157.3715

anova(mod11, mod6)
```

```
## Analysis of Variance Table
##
## Model 1: Transformed ~ Life_stage
## Model 2: Transformed ~ Irradiation_dose * Life_stage
##   Res.Df    RSS Df Sum of Sq      F  Pr(>F)
## 1     45 68.106
## 2     37 45.649  8    22.457 2.2753 0.04327 *
## ---
## Signif. codes:  0 '***' 0.001 '**' 0.01 '*' 0.05 '.' 0.1 ' ' 1
```

```
summary(mod11)
```

```
##
## Call:
## lm(formula = Transformed ~ Life_stage, data = iflaHypox)
##
## Residuals:
##     Min      1Q  Median      3Q     Max
## -2.4594 -0.7253  0.0516  0.6965  3.5962
##
## Coefficients:
##                          Estimate Std. Error t value Pr(>|t|)
## (Intercept)               0.08738    0.25112   0.348    0.729
## Life_stagePupae (0 dpi)  0.05315    0.35898   0.148    0.883
##
## Residual standard error: 1.23 on 45 degrees of freedom
## Multiple R-squared:  0.0004869,  Adjusted R-squared:  -0.02172
## F-statistic: 0.02192 on 1 and 45 DF,  p-value: 0.883
```

```
summary(mod6)
```

```
##
## Call:
## lm(formula = Transformed ~ Irradiation_dose * Life_stage, data =
iflaHypox)
##
## Residuals:
##      Min       1Q   Median       3Q      Max
## -1.87999 -0.58719 -0.00667  0.51079  2.80670
##
## Coefficients:
##                                                  Estimate Std. Error t
value
## (Intercept)                                       0.87683    0.45346
1.934
## Irradiation_dose70                               -2.69181    0.71698  -
3.754
## Irradiation_dose110                              -0.71270    0.64129  -
1.111
## Irradiation_dose150                              -0.49381    0.64129  -
0.770
```

```
## Irradiation_dose190                         -0.47029    0.90692  -
0.519
## Life_stagePupae (0 dpi)                     -1.10198    0.64129  -
1.718
## Irradiation_dose70:Life_stagePupae (0 dpi)   3.48263    1.01397
3.435
## Irradiation_dose110:Life_stagePupae (0 dpi)  1.29996    0.90692
1.433
## Irradiation_dose150:Life_stagePupae (0 dpi)  1.03851    0.92932
1.117
## Irradiation_dose190:Life_stagePupae (0 dpi) -0.02953    1.28258  -
0.023
##                                             Pr(>|t|)
## (Intercept)                                 0.060834 .
## Irradiation_dose70                          0.000597 ***
## Irradiation_dose110                         0.273583
## Irradiation_dose150                         0.446176
## Irradiation_dose190                         0.607158
## Life_stagePupae (0 dpi)                     0.094084 .
## Irradiation_dose70:Life_stagePupae (0 dpi)  0.001478 **
## Irradiation_dose110:Life_stagePupae (0 dpi) 0.160148
## Irradiation_dose150:Life_stagePupae (0 dpi) 0.270985
## Irradiation_dose190:Life_stagePupae (0 dpi) 0.981755
## ---
## Signif. codes:  0 '***' 0.001 '**' 0.01 '*' 0.05 '.' 0.1 ' ' 1
##
## Residual standard error: 1.111 on 37 degrees of freedom
## Multiple R-squared:  0.3301, Adjusted R-squared:  0.1671
## F-statistic: 2.025 on 9 and 37 DF,  p-value: 0.06392

#----------------------------------------------------------------

###Figure hypoxia
label_parse <- function(breaks) {parse(text = breaks)}

FigS2B<-ggplot(iflaHypox, aes(x=Irradiation_dose,y=Transformed, colour=
Life_stage)) +
  geom_boxplot(position=position_dodge(0.8))+
  geom_jitter(position=position_dodge(0.8)) + scale_y_continuous(labels
= label_parse)

FigS2B
```

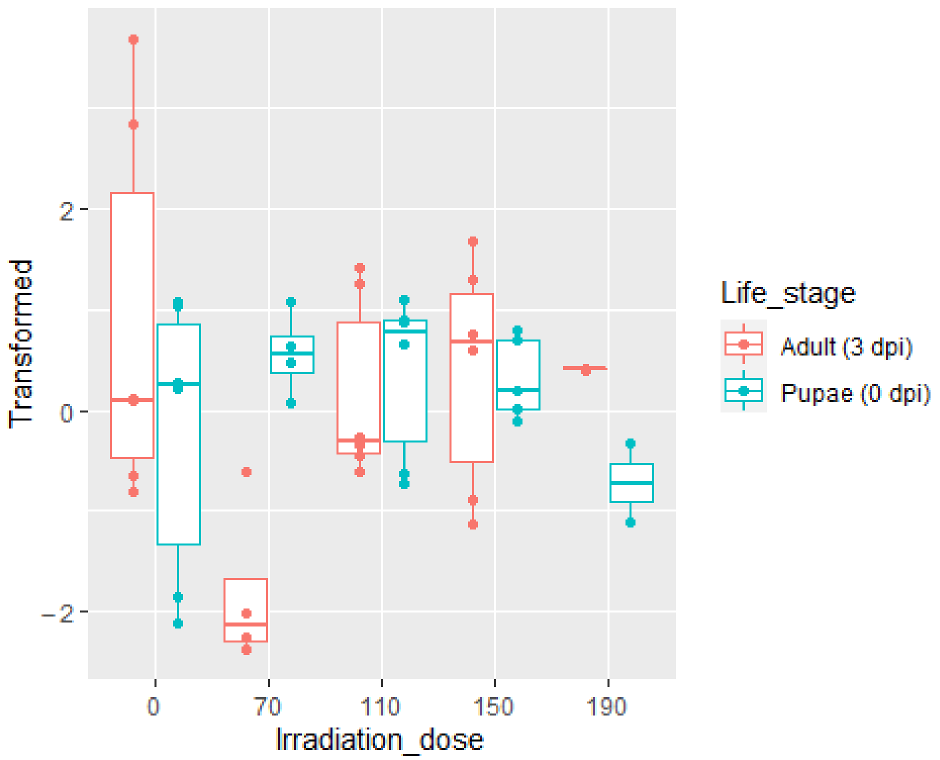


```
tiff("Fig S2B.tiff", width = 7, height = 4, units = 'in',compression =
'lzw',res = 300)
plot(FigS2B+theme_tufte() + theme(axis.line = element_line(size = 1,
colour = "black")) + theme(legend.title= element_text(face = "bold")) +
theme(legend.text = element_text(face = "plain"))) + theme(axis.text.x
= element_text(colour = "black")) + theme(axis.text.y =
element_text(colour = "black")) + xlab(expression(bold("Irradiation
dose (Gy)"))) + ylab(expression(bold("GmmIV_ ")~bold("Hypoxia
Transformed relative density")))+ scale_y_continuous(labels =
label_parse)

## Scale for 'y' is already present. Adding another scale for 'y',
which will
## replace the existing scale.

dev.off()

## png
##   2

#
#work with GmmNegV data

neg<-subset(qpcr, Target=="GmmNegV")
neg

##      Target Sample Irradiation_dose Dpi    Life_stage
Irradiation_conditions
```

```
## 93  GmmNegV    17A                0   3 Adult (3 dpi)
Hypoxia
## 94  GmmNegV    17B                0   3 Adult (3 dpi)
Hypoxia
## 95  GmmNegV    18A                0   3 Adult (3 dpi)
Normoxia
## 96  GmmNegV    18B                0   3 Adult (3 dpi)
Normoxia
## 97  GmmNegV     8A                0   0 Pupae (0 dpi)
Hypoxia
## 98  GmmNegV     8B                0   0 Pupae (0 dpi)
Hypoxia
## 99  GmmNegV     9A                0   0 Pupae (0 dpi)
Normoxia
## 100 GmmNegV     9B                0   0 Pupae (0 dpi)
Normoxia
## 101 GmmNegV    16B                0   0 Pupae (0 dpi)
Hypoxia
## 102 GmmNegV    16B                0   0 Pupae (0 dpi)
Hypoxia
## 103 GmmNegV    17A                0   3 Adult (3 dpi)
Normoxia
## 104 GmmNegV    17B                0   3 Adult (3 dpi)
Normoxia
## 105 GmmNegV     6A                0   3 Adult (3 dpi)
Normoxia
## 106 GmmNegV     6B                0   3 Adult (3 dpi)
Normoxia
## 107 GmmNegV     1A                0   0 Pupae (0 dpi)
Hypoxia
## 108 GmmNegV     1B                0   0 Pupae (0 dpi)
Hypoxia
## 109 GmmNegV     2A                0   3 Adult (3 dpi)
Hypoxia
## 110 GmmNegV     2B                0   3 Adult (3 dpi)
Hypoxia
## 111 GmmNegV     3a                0   3 Adult (3 dpi)
Normoxia
## 112 GmmNegV     3b                0   3 Adult (3 dpi)
Normoxia
## 113 GmmNegV     4a                0   3 Adult (3 dpi)
Hypoxia
## 114 GmmNegV     4b                0   3 Adult (3 dpi)
Hypoxia
## 115 GmmNegV    11A               70   3 Adult (3 dpi)
Hypoxia
## 116 GmmNegV    11B               70   3 Adult (3 dpi)
Hypoxia
## 117 GmmNegV    14A               70   3 Adult (3 dpi)
Normoxia
```

```
## 118 GmmNegV    14B                 70   3 Adult (3 dpi)
Normoxia
## 119 GmmNegV    21A                 70   0 Pupae (0 dpi)
Normoxia
## 120 GmmNegV    21B                 70   0 Pupae (0 dpi)
Normoxia
## 121 GmmNegV     2A                 70   0 Pupae (0 dpi)
Hypoxia
## 122 GmmNegV     2B                 70   0 Pupae (0 dpi)
Hypoxia
## 123 GmmNegV     5A                 70   0 Pupae (0 dpi)
Normoxia
## 124 GmmNegV     5B                 70   0 Pupae (0 dpi)
Normoxia
## 125 GmmNegV    10A                110   0 Pupae (0 dpi)
Hypoxia
## 126 GmmNegV    10B                110   0 Pupae (0 dpi)
Hypoxia
## 127 GmmNegV    13A                 70   0 Pupae (0 dpi)
Normoxia
## 128 GmmNegV    13B                 70   0 Pupae (0 dpi)
Normoxia
## 129 GmmNegV    19A                110   3 Adult (3 dpi)
Hypoxia
## 130 GmmNegV    19B                110   3 Adult (3 dpi)
Hypoxia
## 131 GmmNegV    22A                 70   3 Adult (3 dpi)
Normoxia
## 132 GmmNegV    22B                 70   3 Adult (3 dpi)
Normoxia
## 133 GmmNegV     3A                 70   3 Adult (3 dpi)
Hypoxia
## 134 GmmNegV     3B                 70   3 Adult (3 dpi)
Hypoxia
## 135 GmmNegV     6A                 70   3 Adult (3 dpi)
Normoxia
## 136 GmmNegV     6B                 70   3 Adult (3 dpi)
Normoxia
## 137 GmmNegV     2A                 70   0 Pupae (0 dpi)
Hypoxia
## 138 GmmNegV     2B                 70   0 Pupae (0 dpi)
Hypoxia
## 139 GmmNegV    12A                110   3 Adult (3 dpi)
Hypoxia
## 140 GmmNegV    12B                110   3 Adult (3 dpi)
Hypoxia
## 141 GmmNegV    15A                110   3 Adult (3 dpi)
Normoxia
## 142 GmmNegV    15B                110   3 Adult (3 dpi)
Normoxia
```

```
## 143 GmmNegV    22A              110   0 Pupae (0 dpi)
Normoxia
## 144 GmmNegV    22B              110   0 Pupae (0 dpi)
Normoxia
## 145 GmmNegV     3A              110   0 Pupae (0 dpi)
Hypoxia
## 146 GmmNegV     3B              110   0 Pupae (0 dpi)
Hypoxia
## 147 GmmNegV     6A              110   0 Pupae (0 dpi)
Normoxia
## 148 GmmNegV     6B              110   0 Pupae (0 dpi)
Normoxia
## 149 GmmNegV    11A              150   0 Pupae (0 dpi)
Hypoxia
## 150 GmmNegV    11B              150   0 Pupae (0 dpi)
Hypoxia
## 151 GmmNegV    14A              110   0 Pupae (0 dpi)
Normoxia
## 152 GmmNegV    14B              110   0 Pupae (0 dpi)
Normoxia
## 153 GmmNegV    20A              150   3 Adult (3 dpi)
Hypoxia
## 154 GmmNegV    20B              150   3 Adult (3 dpi)
Hypoxia
## 155 GmmNegV     4A              110   3 Adult (3 dpi)
Hypoxia
## 156 GmmNegV     4B              110   3 Adult (3 dpi)
Hypoxia
## 157 GmmNegV     7A              110   3 Adult (3 dpi)
Normoxia
## 158 GmmNegV     7B              110   3 Adult (3 dpi)
Normoxia
## 159 GmmNegV     3A              110   0 Pupae (0 dpi)
Hypoxia
## 160 GmmNegV     3B              110   0 Pupae (0 dpi)
Hypoxia
## 161 GmmNegV     4A              110   3 Adult (3 dpi)
Normoxia
## 162 GmmNegV     4B              110   3 Adult (3 dpi)
Normoxia
## 163 GmmNegV    13A              150   3 Adult (3 dpi)
Hypoxia
## 164 GmmNegV    13B              150   3 Adult (3 dpi)
Hypoxia
## 165 GmmNegV    16A              150   3 Adult (3 dpi)
Normoxia
## 166 GmmNegV    16B              150   3 Adult (3 dpi)
Normoxia
## 167 GmmNegV    20A              150   0 Pupae (0 dpi)
Hypoxia
```

```
## 168 GmmNegV    20B              150   0 Pupae (0 dpi)
Hypoxia
## 169 GmmNegV    23A              150   0 Pupae (0 dpi)
Normoxia
## 170 GmmNegV    23B              150   0 Pupae (0 dpi)
Normoxia
## 171 GmmNegV     4A              150   0 Pupae (0 dpi)
Hypoxia
## 172 GmmNegV     4B              150   0 Pupae (0 dpi)
Hypoxia
## 173 GmmNegV     7A              150   0 Pupae (0 dpi)
Normoxia
## 174 GmmNegV     7B              150   0 Pupae (0 dpi)
Normoxia
## 175 GmmNegV    12A              190   0 Pupae (0 dpi)
Hypoxia
## 176 GmmNegV    12B              190   0 Pupae (0 dpi)
Hypoxia
## 177 GmmNegV    21A              190   3 Adult (3 dpi)
Hypoxia
## 178 GmmNegV    21B              190   3 Adult (3 dpi)
Hypoxia
## 179 GmmNegV     5A              150   3 Adult (3 dpi)
Hypoxia
## 180 GmmNegV     5B              150   3 Adult (3 dpi)
Hypoxia
## 181 GmmNegV     8A              150   3 Adult (3 dpi)
Normoxia
## 182 GmmNegV     8B              150   3 Adult (3 dpi)
Normoxia
## 183 GmmNegV     5A              150   3 Adult (3 dpi)
Normoxia
## 184 GmmNegV     5B              150   3 Adult (3 dpi)
Normoxia
## 185 GmmNegV     1A                0   0 Pupae (0 dpi)
Normoxia
## 186 GmmNegV     1B                0   0 Pupae (0 dpi)
Normoxia
##     Replicate Expression  Mean.Cq Normalized_expression Transformed
## 93          1   0.810200 26.68000            5.20627169  1.52094764
## 94          1   0.736340 26.76000            4.73165403  1.43950847
## 95          1   0.539510 26.91000            3.46684231  1.16906697
## 96          1   0.486370 27.07000            3.12536949  1.07702116
## 97          1   0.884310 27.01000            0.26036264 -1.44042433
## 98          1   1.173400 26.50000            0.34547786 -1.12136202
## 99          1   2.682830 28.13000            0.78989122 -0.23866353
## 100         1   4.712670 28.02000            1.38752611  0.32221692
## 101         3  10.713230 24.32000            2.31479499  0.80506324
## 102         3   8.776540 24.43000            1.89633666  0.61987867
## 103         3   1.008980 27.78000            5.75491231  1.60547496
```

```
## 104          3   0.903890 28.06000          5.15551119  1.51263603
## 105          3   0.625310 25.68000          3.56657636  1.19407774
## 106          3   0.455630 25.73000          2.59877371  0.91085245
## 107          2   1.484860 26.97000          0.06325263 -3.17929376
## 108          2   1.411240 26.96000          0.06011654 -3.24648346
## 109          2   1.865650 26.34000          0.33823712 -1.14494355
## 110          2   1.666820 26.47000          0.30218980 -1.27124838
## 111          2   1.121410 26.94000          0.20330849 -1.72693311
## 112          2   1.065870 26.71000          0.19323925 -1.78665215
## 113          3   5.410940 25.25000         30.86234137  2.90328911
## 114          3   4.805110 25.12000         27.40687295  2.81851841
## 115          1   0.218900 27.03000          1.40663154  0.33544271
## 116          1   0.200310 27.24000          1.28717389  0.24928915
## 117          1 118.914050 28.95000        764.13089580  4.85147028
## 118          1 105.121200 29.33000        675.49929310  4.78760271
## 119          2  42.612910 26.04000          1.81524102  0.57879247
## 120          2  43.930260 26.02000          1.87135800  0.60743277
## 121          1   1.126520 29.92000          0.33167523 -1.16679892
## 122          1   0.983670 29.74000          0.28961667 -1.31924979
## 123          1  11.207230 25.01000          3.29968452  1.12531886
## 124          1   7.844940 25.13000          2.30974354  0.80305427
## 125          3   2.495140 27.69000          0.53912196 -0.63729731
## 126          3   0.610270 27.92000          0.13186032 -2.24584019
## 127          3   9.263410 23.79000          2.00153409  0.67038546
## 128          3   4.944410 24.38000          1.06833285  0.06588137
## 129          3  11.921640 25.73000         67.99737630  3.44231148
## 130          3   1.895520 25.77000         10.81146442  2.11845159
## 131          3   0.236260 26.01000          1.34755454  0.29388652
## 132          3   0.182220 26.73000          1.03932696  0.03849905
## 133          2   1.013920 25.99000          0.18382086 -1.84569432
## 134          2   0.968270 25.82000          0.17554464 -1.90039133
## 135          2   2.080300 26.05000          0.37715256 -1.02423071
## 136          2   1.804070 26.21000          0.32707284 -1.18241362
## 137          2 118.911610 24.16000          5.06544219  1.49766400
## 138          2  68.661760 24.27000          2.92487988  1.01766607
## 139          1   0.769760 26.10000          4.94640792  1.47742156
## 140          1   0.647620 26.37000          4.16154736  1.32889359
## 141          1   0.279750 26.65000          1.79764812  0.56961265
## 142          1   0.267380 26.72000          1.71815962  0.52686668
## 143          2  54.450680 25.55000          2.31951087  0.80693441
## 144          2  48.545200 25.79000          2.06794697  0.70078990
## 145          1  11.190710 25.10000          3.29482063  1.12400963
## 146          1  13.006450 24.81000          3.82941920  1.25647192
## 147          1   1.223990 26.97000          0.36037280 -1.07451714
## 148          1   1.026830 27.19000          0.30232404 -1.27074779
## 149          3  17.661830 24.45000          3.81617081  1.25344121
## 150          3  11.874990 24.66000          2.56581510  0.89924413
## 151          3   0.305210 29.31000          0.06594637 -3.12444399
## 152          3   0.805570 28.08000          0.17405856 -1.91051283
## 153          3   1.214620 25.03000          6.92781976  1.75975087
```

```
## 154          3   0.416350 25.54000            2.37473264  0.82853891
## 155          2   2.048230 25.96000            0.37133836 -1.04137139
## 156          2   2.110090 25.84000            0.38255341 -1.00856706
## 157          2   0.226830 33.84000            0.04112364 -3.75912578
## 158          2   0.134240 33.91000            0.02433734 -4.50015672
## 159          2  35.677020 25.03000            1.51978333  0.40992878
## 160          2  25.724760 25.20000            1.09583315  0.09109747
## 161          3   0.238190 26.33000            1.35856267  0.30177999
## 162          3   0.186510 26.48000            1.06379581  0.06165263
## 163          1   0.545350 25.99000            3.50436962  1.17856965
## 164          1   0.455380 26.18000            2.92623056  1.01808076
## 165          1   0.600820 27.23000            3.86081480  1.26360819
## 166          1   0.522780 27.15000            3.35933685  1.14120515
## 167          2 106.814450 26.32000            4.55012275  1.40595371
## 168          2  74.982190 26.55000            3.19411998  1.09641565
## 169          2 101.249840 25.20000            4.31307937  1.35985054
## 170          2 111.280710 25.17000            4.74037820  1.44108525
## 171          1   0.661620 27.27000            0.19479722 -1.77719115
## 172          1   0.553050 26.96000            0.16283154 -1.99019225
## 173          1   3.077020 25.60000            0.90595047 -0.09926004
## 174          1   2.600770 25.51000            0.76573074 -0.27051903
## 175          3   6.392100 25.01000            1.38113352  0.31774685
## 176          3   4.698700 25.00000            1.01524258  0.01511614
## 177          3   0.254410 25.98000            1.45107657  0.36546038
## 178          3   0.304950 26.03000            1.73934122  0.53846673
## 179          2  31.843340 28.23000            5.77310837  1.60812456
## 180          2  19.297400 28.22000            3.49856458  1.17710704
## 181          2   5.681461 26.98748            1.03003300  0.02954710
## 182          2   6.501137 27.14410            1.17863801  0.16301621
## 183          3   1.130850 24.78000            6.45002139  1.70065374
## 184          3   0.798040 25.00000            4.55177527  1.40626577
## 185          2  37.974820 25.58000            1.61766589  0.46960025
## 186          2  35.922460 25.59000            1.53023867  0.41650142
```

```
#neg$Life_stage=as.factor(neg$Life_stage)
neg$Irradiation_dose=as.factor(neg$Irradiation_dose)
#select the model

anova(with(neg,lm(Transformed ~ Irradiation_dose)))
```

```
## Analysis of Variance Table
##
## Response: Transformed
##                  Df Sum Sq Mean Sq F value Pr(>F)
## Irradiation_dose  4  12.81  3.2026   1.237  0.301
## Residuals        89 230.43  2.5891
```

```
anova(with(neg,lm(Transformed ~ Life_stage)))
```

```
## Analysis of Variance Table
##
```

```
## Response: Transformed
##            Df  Sum Sq Mean Sq F value Pr(>F)
## Life_stage  1  10.905 10.9047  4.3181 0.0405 *
## Residuals  92 232.335  2.5254
## ---
## Signif. codes:  0 '***' 0.001 '**' 0.01 '*' 0.05 '.' 0.1 ' ' 1
```

```
anova(with(neg,lm(Transformed ~ Irradiation_conditions)))
```

```
## Analysis of Variance Table
##
## Response: Transformed
##                        Df  Sum Sq Mean Sq F value Pr(>F)
## Irradiation_conditions  1   0.009  0.0091  0.0034 0.9533
## Residuals              92 243.230  2.6438
```

```
anova(with(neg,lm(Transformed ~ Irradiation_dose +
Irradiation_conditions)))
```

```
## Analysis of Variance Table
##
## Response: Transformed
##                        Df  Sum Sq Mean Sq F value Pr(>F)
## Irradiation_dose        4  12.810  3.2026  1.2232 0.3068
## Irradiation_conditions  1   0.024  0.0243  0.0093 0.9234
## Residuals              88 230.405  2.6182
```

```
anova(with(neg,lm(Transformed ~ Irradiation_dose+Life_stage)))
```

```
## Analysis of Variance Table
##
## Response: Transformed
##                  Df  Sum Sq Mean Sq F value Pr(>F)
## Irradiation_dose  4  12.810  3.2026  1.2817 0.2833
## Life_stage        1  10.546 10.5462  4.2207 0.0429 *
## Residuals        88 219.883  2.4987
## ---
## Signif. codes:  0 '***' 0.001 '**' 0.01 '*' 0.05 '.' 0.1 ' ' 1
```

```
anova(with(neg,lm(Transformed ~
Irradiation_dose*Irradiation_conditions)))
```

```
## Analysis of Variance Table
##
## Response: Transformed
##                                         Df  Sum Sq Mean Sq F value
Pr(>F)
## Irradiation_dose                         4  12.810  3.2026  1.3288
0.26587
## Irradiation_conditions                   1   0.024  0.0243  0.0101
0.92021
## Irradiation_dose:Irradiation_conditions  3  25.546  8.5154  3.5332
```

```
0.01817 *
## Residuals                                    85 204.859  2.4101
## ---
## Signif. codes:  0 '***' 0.001 '**' 0.01 '*' 0.05 '.' 0.1 ' ' 1
```

```
anova(with(neg,lm(Transformed ~ Irradiation_dose*Life_stage)))
```

```
## Analysis of Variance Table
##
## Response: Transformed
##                              Df  Sum Sq Mean Sq F value  Pr(>F)
## Irradiation_dose              4  12.810  3.2026  1.2537 0.29475
## Life_stage                    1  10.546 10.5462  4.1285 0.04533 *
## Irradiation_dose:Life_stage   4   5.304  1.3261  0.5191 0.72189
## Residuals                    84 214.578  2.5545
## ---
## Signif. codes:  0 '***' 0.001 '**' 0.01 '*' 0.05 '.' 0.1 ' ' 1
```

```
#----------------------------------------------------------------------
----------------------------------------


mod1 <- lm(Transformed ~ Irradiation_dose + Irradiation_conditions,
data = neg)
mod2 <- lm(Transformed ~ Irradiation_dose * Irradiation_conditions,
data = neg)
mod3 <- lm(Transformed ~ Irradiation_dose + Life_stage +
Irradiation_conditions, data = neg)
mod4 <- lm(Transformed ~ Irradiation_dose * Life_stage *
Irradiation_conditions, data = neg)
mod5 <- lm(Transformed ~ Irradiation_dose + Life_stage, data = neg)
mod6 <- lm(Transformed ~ Irradiation_dose*Life_stage, data = neg)
mod7 <- lm(Transformed ~ Irradiation_dose, data = neg)
mod8 <- lm(Transformed ~ Irradiation_conditions, data = neg)
mod9 <- lm(Transformed ~  Life_stage, data = neg)
mod10<- lm(Transformed ~ Irradiation_dose+Life_stage, data = neg)

AICc(mod1,mod2,mod3,mod4,mod5,mod6,mod7,mod8,mod9, mod10)
```

```
##       df     AICc
## mod1   7 366.3377
## mod2  10 362.6394
## mod3   8 364.3339
## mod4  19 375.4282
## mod5   7 361.9439
## mod6  11 369.5657
## mod7   6 364.0109
## mod8   3 362.3942
## mod9   3 358.0862
## mod10  7 361.9439
```

```
anova(mod8, mod9)

## Analysis of Variance Table
##
## Model 1: Transformed ~ Irradiation_conditions
## Model 2: Transformed ~ Life_stage
##   Res.Df    RSS Df Sum of Sq F Pr(>F)
## 1     92 243.23
## 2     92 232.34  0    10.896

##no difference, choose either model but went for the lowest

summary(mod8)

##
## Call:
## lm(formula = Transformed ~ Irradiation_conditions, data = neg)
##
## Residuals:
##     Min      1Q  Median      3Q     Max
## -4.7611 -1.2745  0.2815  0.9369  4.5905
##
## Coefficients:
##                                Estimate Std. Error t value Pr(>|t|)
## (Intercept)                     0.24128    0.23469   1.028    0.307
## Irradiation_conditionsNormoxia  0.01968    0.33549   0.059    0.953
##
## Residual standard error: 1.626 on 92 degrees of freedom
## Multiple R-squared:  3.742e-05,  Adjusted R-squared:  -0.01083
## F-statistic: 0.003443 on 1 and 92 DF,  p-value: 0.9533

summary(mod9)

##
## Call:
## lm(formula = Transformed ~ Life_stage, data = neg)
##
## Residuals:
##     Min      1Q  Median      3Q     Max
## -5.0706 -0.9975  0.4321  0.9186  4.2810
##
## Coefficients:
##                          Estimate Std. Error t value Pr(>|t|)
## (Intercept)                0.5704     0.2247   2.538   0.0128 *
## Life_stagePupae (0 dpi)   -0.6826     0.3285  -2.078   0.0405 *
## ---
## Signif. codes:  0 '***' 0.001 '**' 0.01 '*' 0.05 '.' 0.1 ' ' 1
##
## Residual standard error: 1.589 on 92 degrees of freedom
## Multiple R-squared:  0.04483,    Adjusted R-squared:  0.03445
## F-statistic: 4.318 on 1 and 92 DF,  p-value: 0.0405
```

```
#----------------------------------------------------------------------
--------

#======================================================================
==============

neg$Irradiation_dose=as.factor(neg$Irradiation_dose)

label_parse <- function(breaks) {parse(text = breaks)}

Fig3a<-ggplot(neg,aes(x=Irradiation_dose,y=Transformed,
fill=Irradiation_dose)) +
  geom_boxplot() + geom_jitter(width=0.1,alpha=0.2)+
scale_y_continuous(labels = label_parse)
Fig3a
```

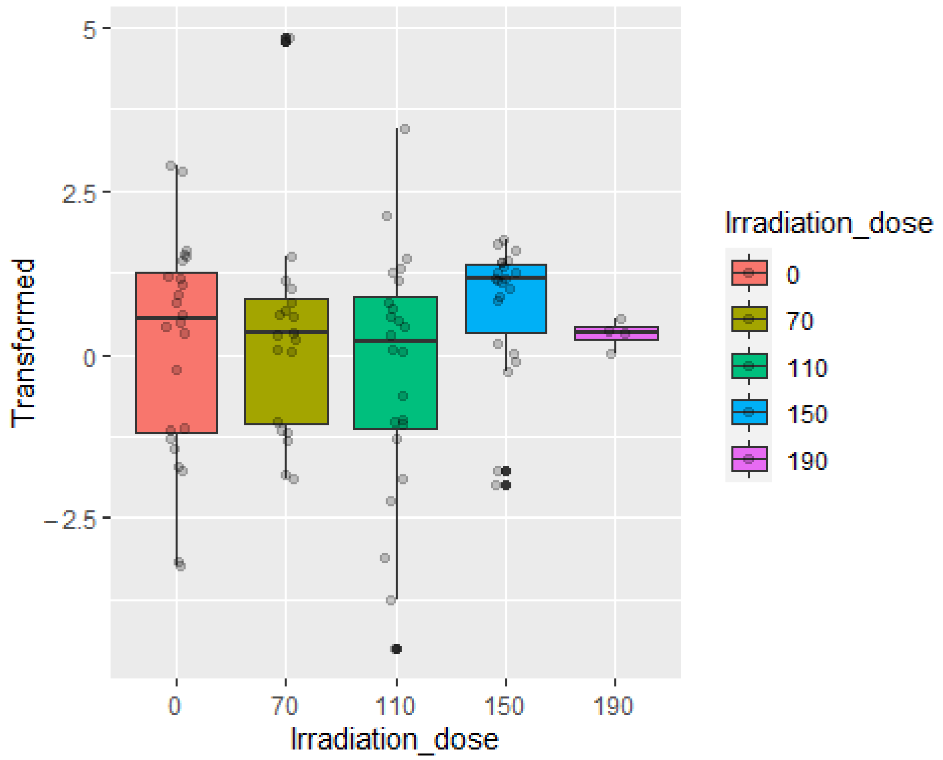


```
tiff("Fig 3a.tiff", width = 4, height = 4, units = 'in', res = 300)
plot(Fig3a+theme_tufte() + theme(axis.line = element_line(size = 1,
colour = "black")) + theme(axis.text.x = element_blank())
+theme(legend.title = element_text(face = "bold")) + theme(legend.text
= element_text(face = "plain"))) + xlab(expression(bold("Irradiation
doses (Gy)"))) + ylab(expression (bold("Transformed relative GmmNegeV
density")))
dev.off()

## png
##   2
```

```
###Irradiation_conditions
#neg$Irradiation_conditions=as.factor(neg$Irradiation_conditions)

label_parse <- function(breaks) {parse(text = breaks)}

Fig3b<-ggplot(neg,aes(x=Irradiation_conditions,y=Transformed,
fill=Irradiation_conditions)) +
  geom_boxplot() + geom_jitter(width=0.1,alpha=0.2)+
scale_y_continuous(labels = label_parse)
Fig3b
```

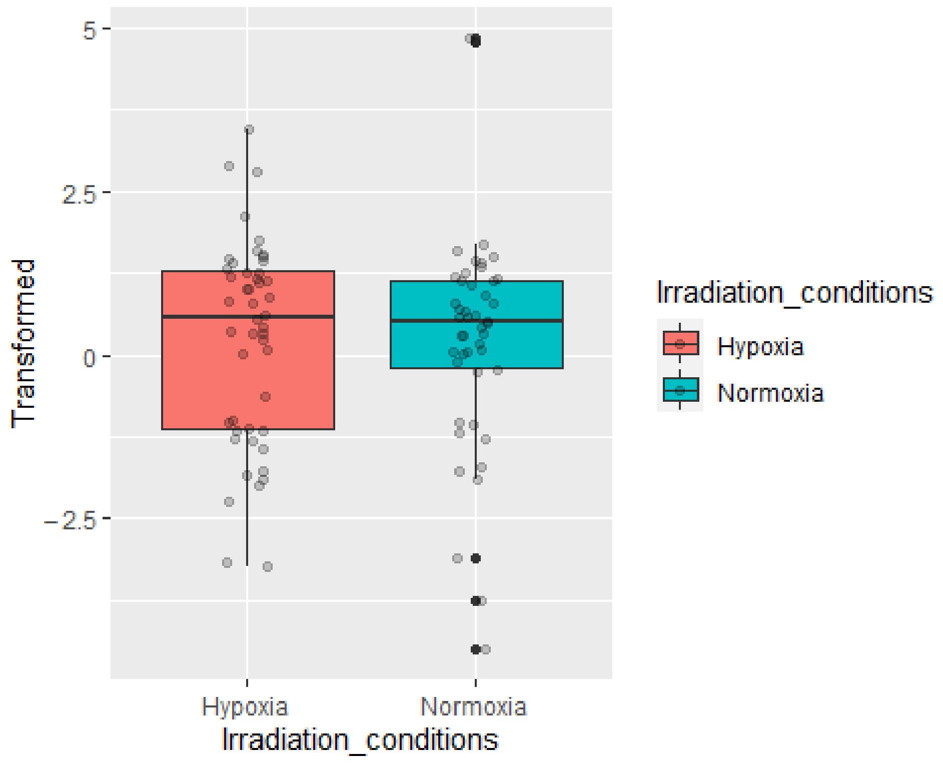


```
tiff("Fig 3b.tiff", width = 4, height = 4, units = 'in', res = 300)
plot(Fig3b+theme_tufte() + theme(axis.line = element_line(size = 1,
colour = "black")) + theme(axis.text.x = element_blank())
+theme(legend.title = element_text(face = "bold")) + theme(legend.text
= element_text(face = "plain"))) + xlab(expression(bold("Irradiation
status"))) + ylab(expression (bold("Transformed relative GmmNegeV
density")))
dev.off()

## png
##   2

###Days_post.irradiation
###neg$Life_stage=as.factor(neg$Life_stage)

label_parse <- function(breaks) {parse(text = breaks)}
```

```
Fig3c<-ggplot(neg,aes(x=Life_stage,y=Transformed, fill=Life_stage)) +
  geom_boxplot() + geom_jitter(width=0.1,alpha=0.2)+
scale_y_continuous(labels = label_parse)
Fig3c
```

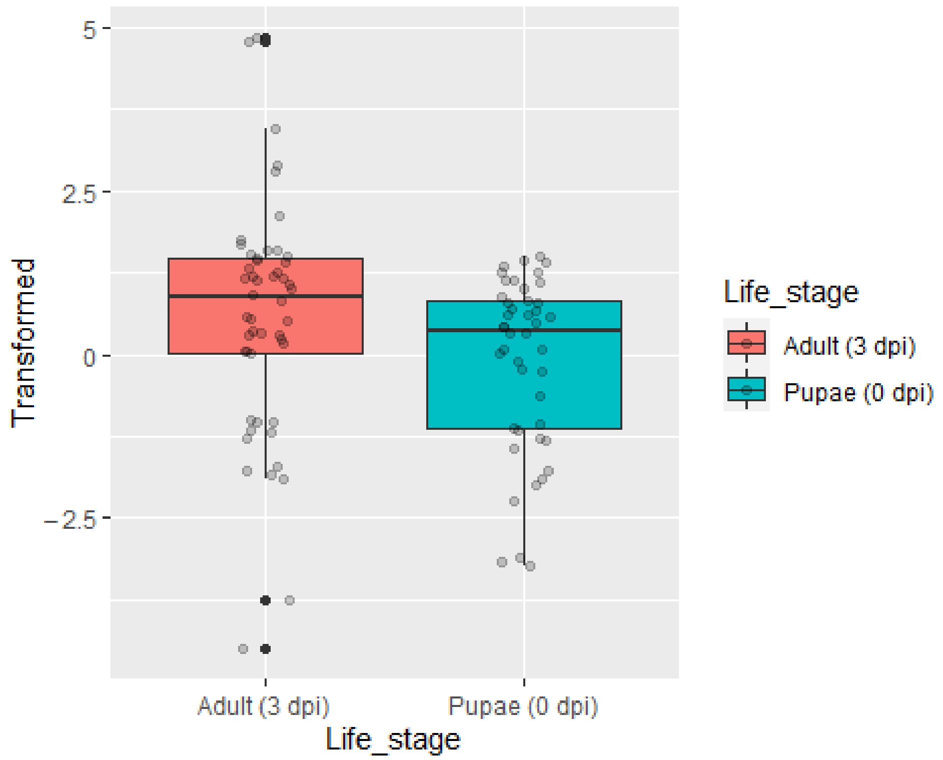


```
tiff("Fig 3c.tiff", width = 4, height = 4, units = 'in', res = 300)
plot(Fig3c+theme_tufte() + theme(axis.line = element_line(size = 1,
colour = "black")) + theme(axis.text.x = element_blank())
+theme(legend.title = element_text(face = "bold")) + theme(legend.text
= element_text(face = "plain"))) + xlab(expression(bold("Days post
irradiation"))) + ylab(expression (bold("Transformed relative GmmNegeV
density")))
dev.off()

## png
##   2

###Irradiation dose according to irradiation status
label_parse <- function(breaks) {parse(text = breaks)}

Fig4a<-ggplot(neg, aes(x=Irradiation_dose,y=Transformed,
colour=Irradiation_conditions)) +
  geom_boxplot(position=position_dodge(0.8))+
  geom_jitter(position=position_dodge(0.8))+ scale_y_continuous(labels
= label_parse)
```

```
Fig4a
```

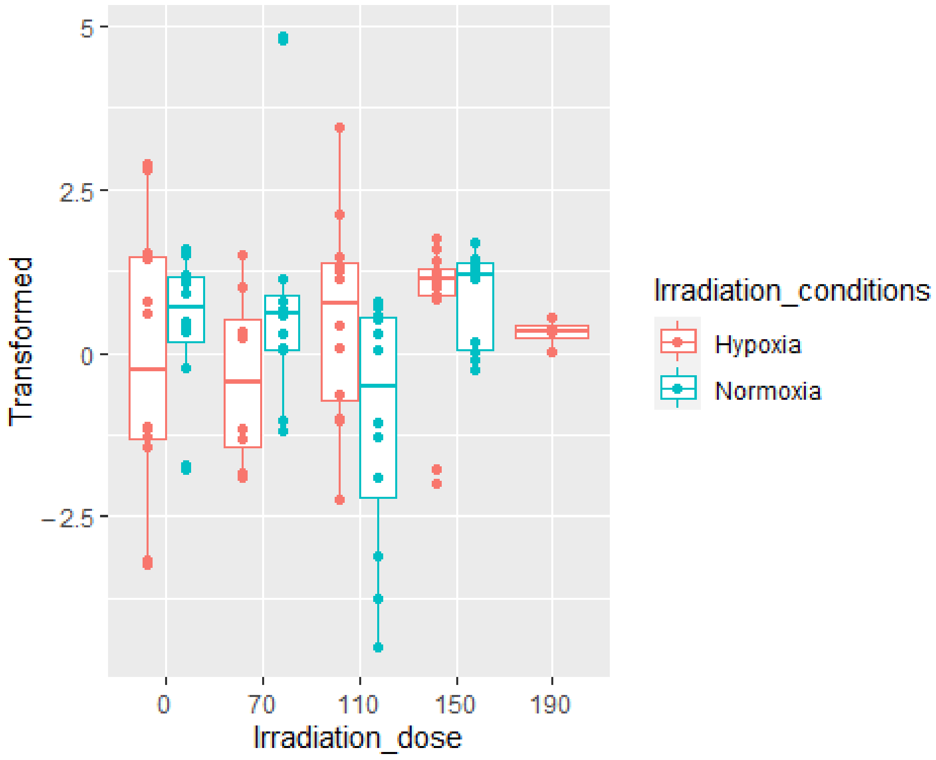


```
tiff("Fig 4a.tiff", width = 7, height = 4, units = 'in',compression =
'lzw',res = 300)
plot(Fig4a+theme_tufte() + theme(axis.line = element_line(size = 1,
colour = "black")) + theme(legend.title= element_text(face = "bold")) +
theme(legend.text = element_text(face = "plain"))) + theme(axis.text.x
= element_text(colour = "black")) + theme(axis.text.y =
element_text(colour = "black")) + xlab(expression(bold("Irradiation
dose (Gy)"))) + ylab(expression(bold("GmmNegeV_ ")~bold("Transformed
normalized density")))
dev.off()

## png
##   2

###Irradiation dose according to days post irradiation
label_parse <- function(breaks) {parse(text = breaks)}

Fig4b<-ggplot(neg, aes(x=Irradiation_dose,y=Transformed,
colour=Life_stage)) +
  geom_boxplot(position=position_dodge(0.8))+
  geom_jitter(position=position_dodge(0.8))+ scale_y_continuous(labels
= label_parse)

Fig4b
```

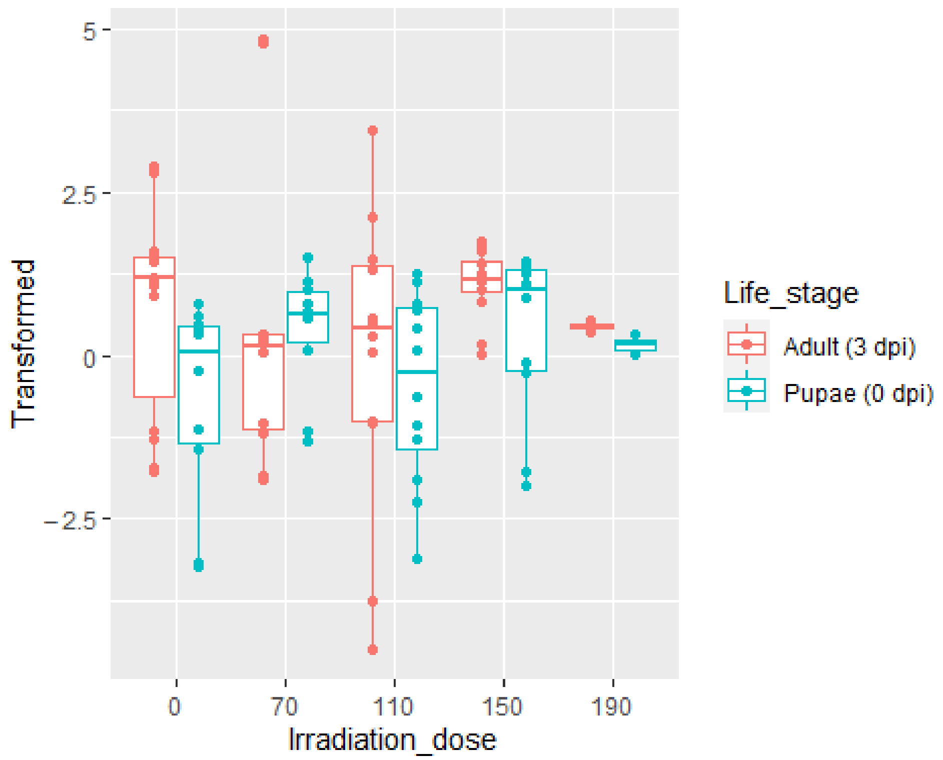


```
tiff("Fig 4b.tiff", width = 7, height = 4, units = 'in',compression =
'lzw',res = 300)
plot(Fig4b+theme_tufte() + theme(axis.line = element_line(size = 1,
colour = "black")) + theme(legend.title= element_text(face = "bold")) +
theme(legend.text = element_text(face = "plain"))) + theme(axis.text.x
= element_text(colour = "black")) + theme(axis.text.y =
element_text(colour = "black")) + xlab(expression(bold("Irradiation
dose (Gy)"))) + ylab(expression(bold ("GmmNegeV_ ")~bold("Transformed
normalized density")))
dev.off()

## png
##   2

#--------------------------------------------------------------------

#GmmNegV-Normoxia
#--------------------------------------------
#NegNorm<- read.csv("GmmNegV_alldata_normalized.csv")
NegNorm<- subset(neg, Irradiation_conditions=="Normoxia")
NegNorm

##       Target Sample Irradiation_dose Dpi    Life_stage
Irradiation_conditions
## 95   GmmNegV    18A                0   3 Adult (3 dpi)
Normoxia
## 96   GmmNegV    18B                0   3 Adult (3 dpi)
```

```
Normoxia
## 99  GmmNegV     9A                0   0 Pupae (0 dpi)
Normoxia
## 100 GmmNegV     9B                0   0 Pupae (0 dpi)
Normoxia
## 103 GmmNegV    17A                0   3 Adult (3 dpi)
Normoxia
## 104 GmmNegV    17B                0   3 Adult (3 dpi)
Normoxia
## 105 GmmNegV     6A                0   3 Adult (3 dpi)
Normoxia
## 106 GmmNegV     6B                0   3 Adult (3 dpi)
Normoxia
## 111 GmmNegV     3a                0   3 Adult (3 dpi)
Normoxia
## 112 GmmNegV     3b                0   3 Adult (3 dpi)
Normoxia
## 117 GmmNegV    14A               70   3 Adult (3 dpi)
Normoxia
## 118 GmmNegV    14B               70   3 Adult (3 dpi)
Normoxia
## 119 GmmNegV    21A               70   0 Pupae (0 dpi)
Normoxia
## 120 GmmNegV    21B               70   0 Pupae (0 dpi)
Normoxia
## 123 GmmNegV     5A               70   0 Pupae (0 dpi)
Normoxia
## 124 GmmNegV     5B               70   0 Pupae (0 dpi)
Normoxia
## 127 GmmNegV    13A               70   0 Pupae (0 dpi)
Normoxia
## 128 GmmNegV    13B               70   0 Pupae (0 dpi)
Normoxia
## 131 GmmNegV    22A               70   3 Adult (3 dpi)
Normoxia
## 132 GmmNegV    22B               70   3 Adult (3 dpi)
Normoxia
## 135 GmmNegV     6A               70   3 Adult (3 dpi)
Normoxia
## 136 GmmNegV     6B               70   3 Adult (3 dpi)
Normoxia
## 141 GmmNegV    15A              110   3 Adult (3 dpi)
Normoxia
## 142 GmmNegV    15B              110   3 Adult (3 dpi)
Normoxia
## 143 GmmNegV    22A              110   0 Pupae (0 dpi)
Normoxia
## 144 GmmNegV    22B              110   0 Pupae (0 dpi)
Normoxia
## 147 GmmNegV     6A              110   0 Pupae (0 dpi)
```

```
Normoxia
## 148 GmmNegV     6B              110   0 Pupae (0 dpi)
Normoxia
## 151 GmmNegV    14A              110   0 Pupae (0 dpi)
Normoxia
## 152 GmmNegV    14B              110   0 Pupae (0 dpi)
Normoxia
## 157 GmmNegV     7A              110   3 Adult (3 dpi)
Normoxia
## 158 GmmNegV     7B              110   3 Adult (3 dpi)
Normoxia
## 161 GmmNegV     4A              110   3 Adult (3 dpi)
Normoxia
## 162 GmmNegV     4B              110   3 Adult (3 dpi)
Normoxia
## 165 GmmNegV    16A              150   3 Adult (3 dpi)
Normoxia
## 166 GmmNegV    16B              150   3 Adult (3 dpi)
Normoxia
## 169 GmmNegV    23A              150   0 Pupae (0 dpi)
Normoxia
## 170 GmmNegV    23B              150   0 Pupae (0 dpi)
Normoxia
## 173 GmmNegV     7A              150   0 Pupae (0 dpi)
Normoxia
## 174 GmmNegV     7B              150   0 Pupae (0 dpi)
Normoxia
## 181 GmmNegV     8A              150   3 Adult (3 dpi)
Normoxia
## 182 GmmNegV     8B              150   3 Adult (3 dpi)
Normoxia
## 183 GmmNegV     5A              150   3 Adult (3 dpi)
Normoxia
## 184 GmmNegV     5B              150   3 Adult (3 dpi)
Normoxia
## 185 GmmNegV     1A                0   0 Pupae (0 dpi)
Normoxia
## 186 GmmNegV     1B                0   0 Pupae (0 dpi)
Normoxia
##     Replicate Expression  Mean.Cq Normalized_expression Transformed
## 95          1   0.539510 26.91000            3.46684231  1.16906697
## 96          1   0.486370 27.07000            3.12536949  1.07702116
## 99          1   2.682830 28.13000            0.78989122 -0.23866353
## 100         1   4.712670 28.02000            1.38752611  0.32221692
## 103         3   1.008980 27.78000            5.75491231  1.60547496
## 104         3   0.903890 28.06000            5.15551119  1.51263603
## 105         3   0.625310 25.68000            3.56657636  1.19407774
## 106         3   0.455630 25.73000            2.59877371  0.91085245
## 111         2   1.121410 26.94000            0.20330849 -1.72693311
## 112         2   1.065870 26.71000            0.19323925 -1.78665215
```

```
## 117          1 118.914050 28.95000          764.13089580  4.85147028
## 118          1 105.121200 29.33000          675.49929310  4.78760271
## 119          2  42.612910 26.04000            1.81524102  0.57879247
## 120          2  43.930260 26.02000            1.87135800  0.60743277
## 123          1  11.207230 25.01000            3.29968452  1.12531886
## 124          1   7.844940 25.13000            2.30974354  0.80305427
## 127          3   9.263410 23.79000            2.00153409  0.67038546
## 128          3   4.944410 24.38000            1.06833285  0.06588137
## 131          3   0.236260 26.01000            1.34755454  0.29388652
## 132          3   0.182220 26.73000            1.03932696  0.03849905
## 135          2   2.080300 26.05000            0.37715256 -1.02423071
## 136          2   1.804070 26.21000            0.32707284 -1.18241362
## 141          1   0.279750 26.65000            1.79764812  0.56961265
## 142          1   0.267380 26.72000            1.71815962  0.52686668
## 143          2  54.450680 25.55000            2.31951087  0.80693441
## 144          2  48.545200 25.79000            2.06794697  0.70078990
## 147          1   1.223990 26.97000            0.36037280 -1.07451714
## 148          1   1.026830 27.19000            0.30232404 -1.27074779
## 151          3   0.305210 29.31000            0.06594637 -3.12444399
## 152          3   0.805570 28.08000            0.17405856 -1.91051283
## 157          2   0.226830 33.84000            0.04112364 -3.75912578
## 158          2   0.134240 33.91000            0.02433734 -4.50015672
## 161          3   0.238190 26.33000            1.35856267  0.30177999
## 162          3   0.186510 26.48000            1.06379581  0.06165263
## 165          1   0.600820 27.23000            3.86081480  1.26360819
## 166          1   0.522780 27.15000            3.35933685  1.14120515
## 169          2 101.249840 25.20000            4.31307937  1.35985054
## 170          2 111.280710 25.17000            4.74037820  1.44108525
## 173          1   3.077020 25.60000            0.90595047 -0.09926004
## 174          1   2.600770 25.51000            0.76573074 -0.27051903
## 181          2   5.681461 26.98748            1.03003300  0.02954710
## 182          2   6.501137 27.14410            1.17863801  0.16301621
## 183          3   1.130850 24.78000            6.45002139  1.70065374
## 184          3   0.798040 25.00000            4.55177527  1.40626577
## 185          2  37.974820 25.58000            1.61766589  0.46960025
## 186          2  35.922460 25.59000            1.53023867  0.41650142

#-------------------------------------------------------------------
anova(with(NegNorm,lm(Transformed~ Life_stage*Irradiation_dose)))

## Analysis of Variance Table
##
## Response: Transformed
##                              Df Sum Sq Mean Sq F value Pr(>F)
## Life_stage                    1  1.305  1.3045  0.5080 0.4804
## Irradiation_dose              3 29.635  9.8783  3.8467 0.0169 *
## Life_stage:Irradiation_dose   3  0.993  0.3309  0.1289 0.9424
## Residuals                    38 97.582  2.5680
## ---
## Signif. codes:  0 '***' 0.001 '**' 0.01 '*' 0.05 '.' 0.1 ' ' 1
```

```
anova(with(NegNorm,lm(Transformed~ Life_stage +Irradiation_dose)))

## Analysis of Variance Table
##
## Response: Transformed
##                  Df Sum Sq Mean Sq F value  Pr(>F)
## Life_stage        1  1.305  1.3045  0.5426 0.46556
## Irradiation_dose  3 29.635  9.8783  4.1086 0.01226 *
## Residuals        41 98.575  2.4043
## ---
## Signif. codes:  0 '***' 0.001 '**' 0.01 '*' 0.05 '.' 0.1 ' ' 1

anova(with(NegNorm,lm(Transformed~  Life_stage)))

## Analysis of Variance Table
##
## Response: Transformed
##            Df  Sum Sq Mean Sq F value Pr(>F)
## Life_stage  1   1.305  1.3045  0.4477 0.5069
## Residuals  44 128.210  2.9139

anova(with(NegNorm,lm(Transformed~  Irradiation_dose)))

## Analysis of Variance Table
##
## Response: Transformed
##                  Df Sum Sq Mean Sq F value  Pr(>F)
## Irradiation_dose  3 30.132 10.0441  4.2447 0.01045 *
## Residuals        42 99.382  2.3662
## ---
## Signif. codes:  0 '***' 0.001 '**' 0.01 '*' 0.05 '.' 0.1 ' ' 1

mod1 <- lm(Transformed ~ Irradiation_dose, data = NegNorm)
mod5 <- lm(Transformed~ Irradiation_dose+Life_stage, data = NegNorm)
mod6 <- lm(Transformed ~ Irradiation_dose*Life_stage, data = NegNorm)
mod11 <- lm(Transformed ~Life_stage, data = NegNorm)


AICc(mod1, mod5, mod6, mod11)

##       df     AICc
## mod1   5 177.4776
## mod5   6 179.7564
## mod6   9 188.1370
## mod11  3 184.2651

anova(mod1, mod5)

## Analysis of Variance Table
##
## Model 1: Transformed ~ Irradiation_dose
## Model 2: Transformed ~ Irradiation_dose + Life_stage
```

```
##   Res.Df    RSS Df Sum of Sq      F Pr(>F)
## 1     42 99.382
## 2     41 98.575  1   0.80711 0.3357 0.5655
```

```
summary(mod1)
```

```
##
## Call:
## lm(formula = Transformed ~ Irradiation_dose, data = NegNorm)
##
## Residuals:
##     Min      1Q  Median      3Q     Max
## -3.4442 -0.8369 -0.0062  0.7774  3.8835
##
## Coefficients:
##                     Estimate Std. Error t value Pr(>|t|)
## (Intercept)           0.4104     0.4441   0.924   0.3606
## Irradiation_dose70    0.5575     0.6280   0.888   0.3797
## Irradiation_dose110  -1.4664     0.6280  -2.335   0.0244 *
## Irradiation_dose150   0.4031     0.6586   0.612   0.5438
## ---
## Signif. codes:  0 '***' 0.001 '**' 0.01 '*' 0.05 '.' 0.1 ' ' 1
##
## Residual standard error: 1.538 on 42 degrees of freedom
## Multiple R-squared:  0.2327, Adjusted R-squared:  0.1778
## F-statistic: 4.245 on 3 and 42 DF,  p-value: 0.01045
```

```
#-------------------------------------------------------------

#FIGURE_normoxia
###NegNorm$Life_stage=as.factor(NegNorm$Life_stage)

label_parse <- function(breaks) {parse(text = breaks)}

FigsS3A<-ggplot(NegNorm, aes(x=Irradiation_dose,y=Transformed, colour=
Life_stage
)) +geom_boxplot(position=position_dodge(0.8))+
  geom_jitter(position=position_dodge(0.8))+ scale_y_continuous(labels
= label_parse)

FigsS3A
```

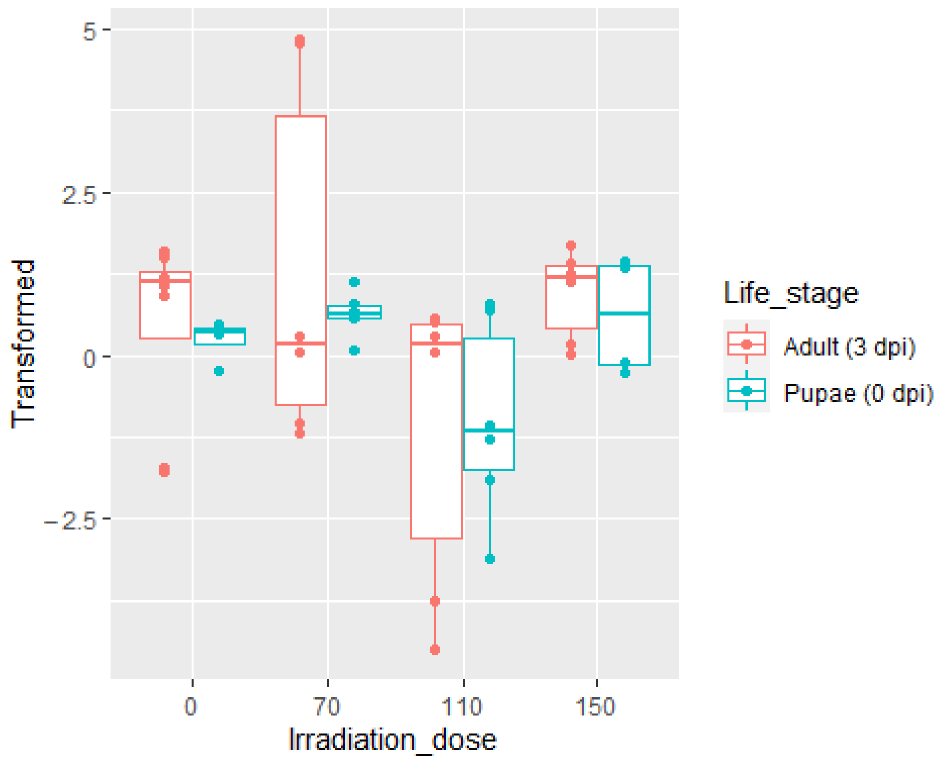


```
tiff("Fig S3A.tiff", width = 7, height = 4, units = 'in',compression =
'lzw',res = 300)
plot(FigsS3A+theme_tufte() + theme(axis.line = element_line(size = 1,
colour = "black")) + theme(legend.title= element_text(face = "bold")) +
theme(legend.text = element_text(face = "plain"))) + theme(axis.text.x
= element_text(colour = "black")) + theme(axis.text.y =
element_text(colour = "black")) + xlab(expression(bold("Irradiation
dose (Gy)"))) + ylab(expression(bold("GmmNegeV_ ")~bold("Normoxia
transformed relative density")))
dev.off()

## png
##   2

#-----------------------------------------------
#NegHypox <- read.csv("GmmNegV_alldata_normalized.csv")
NegHypox <- subset(neg , Irradiation_conditions=="Hypoxia")

#---------------------------------------------------------------------
---
anova(with(NegHypox,lm(Transformed~  Life_stage+Irradiation_dose)))

## Analysis of Variance Table
##
## Response: Transformed
##                     Df Sum Sq Mean Sq F value  Pr(>F)
## Life_stage           1 12.211 12.2113  5.4991 0.02383 *
```

```
## Irradiation_dose  4  8.239  2.0598  0.9276 0.45711
## Residuals        42 93.265  2.2206
## ---
## Signif. codes:  0 '***' 0.001 '**' 0.01 '*' 0.05 '.' 0.1 ' ' 1

anova(with(NegHypox,lm(Transformed~  Life_stage*Irradiation_dose)))

## Analysis of Variance Table
##
## Response: Transformed
##                             Df Sum Sq Mean Sq F value  Pr(>F)
## Life_stage                   1 12.211 12.2113  5.7190 0.02184 *
## Irradiation_dose             4  8.239  2.0598  0.9647 0.43804
## Life_stage:Irradiation_dose  4 12.127  3.0318  1.4199 0.24602
## Residuals                   38 81.138  2.1352
## ---
## Signif. codes:  0 '***' 0.001 '**' 0.01 '*' 0.05 '.' 0.1 ' ' 1

anova(with(NegHypox,lm(Transformed~  Life_stage)))

## Analysis of Variance Table
##
## Response: Transformed
##            Df  Sum Sq Mean Sq F value  Pr(>F)
## Life_stage  1  12.211 12.2113   5.534 0.02299 *
## Residuals  46 101.504  2.2066
## ---
## Signif. codes:  0 '***' 0.001 '**' 0.01 '*' 0.05 '.' 0.1 ' ' 1

anova(with(NegHypox,lm(Transformed~  Irradiation_dose)))

## Analysis of Variance Table
##
## Response: Transformed
##                  Df  Sum Sq Mean Sq F value Pr(>F)
## Irradiation_dose  4   8.239  2.0598  0.8397 0.5076
## Residuals        43 105.476  2.4529

mod1 <- lm(Transformed ~ Irradiation_dose, data = NegHypox)
mod5 <- lm(Transformed~ Irradiation_dose+Life_stage, data = NegHypox)
mod6 <- lm(Transformed ~ Irradiation_dose*Life_stage, data = NegHypox)
mod11 <- lm(Transformed ~Life_stage, data = NegHypox)

AICc(mod1, mod5, mod6, mod11)

##       df     AICc
## mod1   6 188.0566
## mod5   7 184.9018
## mod6  11 190.7488
## mod11  3 178.7108

anova(mod11,mod5)
```

```
## Analysis of Variance Table
##
## Model 1: Transformed ~ Life_stage
## Model 2: Transformed ~ Irradiation_dose + Life_stage
##   Res.Df     RSS Df Sum of Sq      F Pr(>F)
## 1     46 101.504
## 2     42  93.265  4    8.2394 0.9276 0.4571
```

```
summary(mod11)
```

```
##
## Call:
## lm(formula = Transformed ~ Life_stage, data = NegHypox)
##
## Residuals:
##     Min      1Q  Median      3Q     Max
## -2.9834 -1.0864  0.3928  1.0917  2.6966
##
## Coefficients:
##                           Estimate Std. Error t value Pr(>|t|)
## (Intercept)                 0.7457     0.3032   2.459   0.0177 *
## Life_stagePupae (0 dpi)  -1.0088     0.4288  -2.352   0.0230 *
## ---
## Signif. codes:  0 '***' 0.001 '**' 0.01 '*' 0.05 '.' 0.1 ' ' 1
##
## Residual standard error: 1.485 on 46 degrees of freedom
## Multiple R-squared:  0.1074, Adjusted R-squared:  0.08798
## F-statistic: 5.534 on 1 and 46 DF,  p-value: 0.02299
```

```
#----------------------------------------------------------------

###Figure hypoxia
label_parse <- function(breaks) {parse(text = breaks)}

FigS3B<-ggplot(NegHypox, aes(x=Irradiation_dose,y=Transformed, colour=
Life_stage)) +
  geom_boxplot(position=position_dodge(0.8))+
  geom_jitter(position=position_dodge(0.8))+ scale_y_continuous(labels
= label_parse)

FigS3B
```

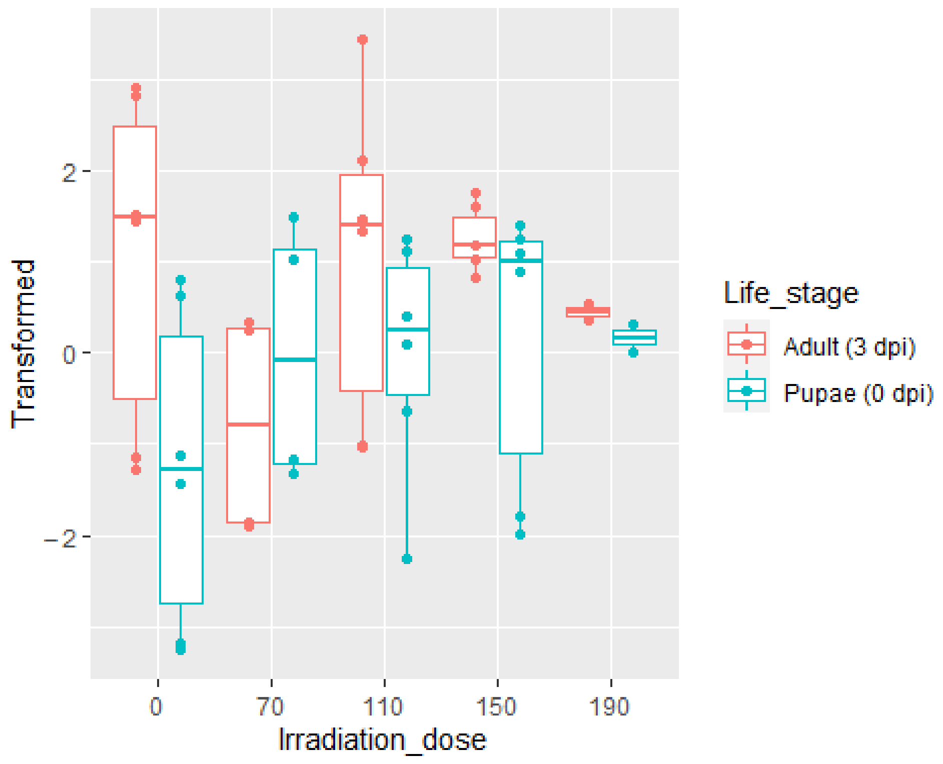


```
tiff("Fig S3B.tiff", width = 7, height = 4, units = 'in',compression =
'lzw',res = 300)
plot(FigS3B+theme_tufte() + theme(axis.line = element_line(size = 1,
colour = "black")) + theme(legend.title= element_text(face = "bold")) +
theme(legend.text = element_text(face = "plain"))) + theme(axis.text.x
= element_text(colour = "black")) + theme(axis.text.y =
element_text(colour = "black")) + xlab(expression(bold("Irradiation
dose (Gy)"))) + ylab(expression(bold("GmmNegeV_ ")~bold("Hypoxia
Transformed relative density")))
dev.off()

## png
##   2
```
