# Supplementary material for: Evaluating the Effect of Irradiation on the Densities of Two RNA Viruses in Glossina morsitans morsitans
Source: Insects. 2023 Apr 20;14(4):397. doi: 10.3390/insects14040397 (PMC10140815; doi:10.3390/insects14040397)
Supplement: Supplementary file 1 [file insects-14-00397-s001.zip › Supplementary Figures proofs.pdf]

Supplementary Figures

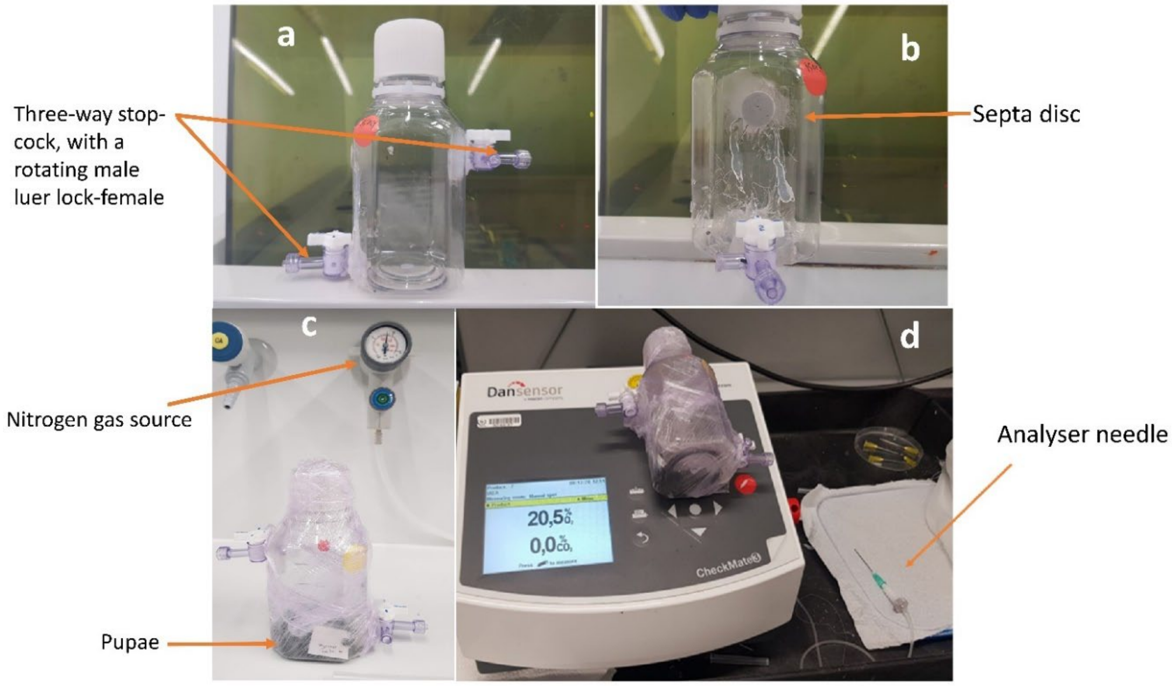

Supplementary Figure S1. Materials and equipment to apply a hypoxic environment before irradiation of pupae. Male pupae were irradiated after the emergence of female pupae, in the presence or absence of oxygen (normoxia) by displacement with nitrogen. The irradiation bottles were fastened with a cling wrap paper to ensure air-tightness all around the bottle, especially after insertion of the gas analyser needle. a) Irradiation bottle for application of hypoxia (viewing angle for the 3-way stop cocks), b) Irradiation bottle for application of hypoxia (viewing angle for septa disc where the analyser needle is inserted), c) Irradiation bottle for the application of hypoxia, containing pupae before irradiation, d) Gas analyser (check mate 3) with a needle for measurement of oxygen levels before and after irradiation. The analyser shows oxygen reading before insertion of needle for measurement of oxygen and carbon-dioxide levels.

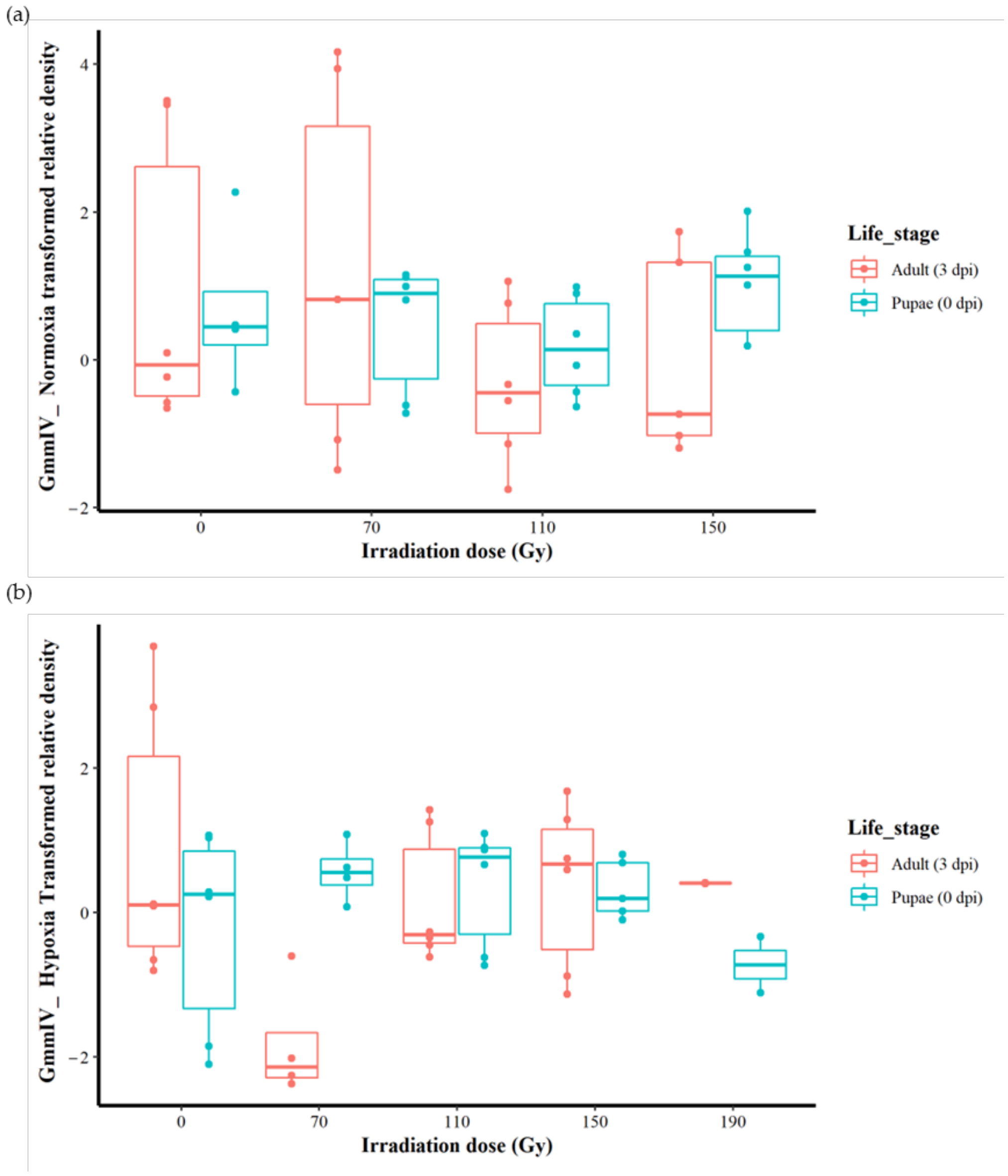

Supplementary Figure S2. Effect irradiation doses on the density of GmmIV in different life stage of *G. morsitans morsitans* following irradiation in different irradiation conditions. a) under normoxia, b) Under hypoxia conditions. Differences in the density of GmmIV as affected by irradiation doses, a comparison between the adult (red) and pupae (blue) stages.

(a)

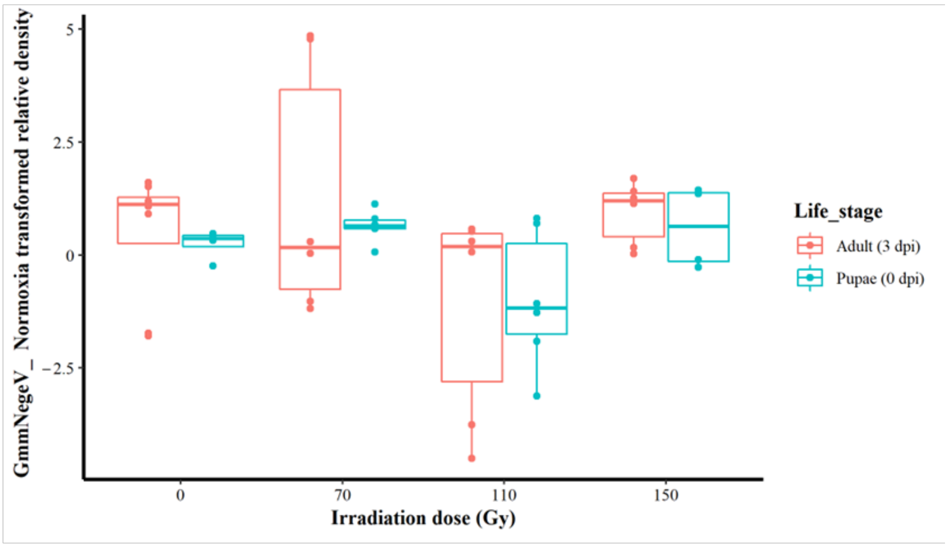

(b)

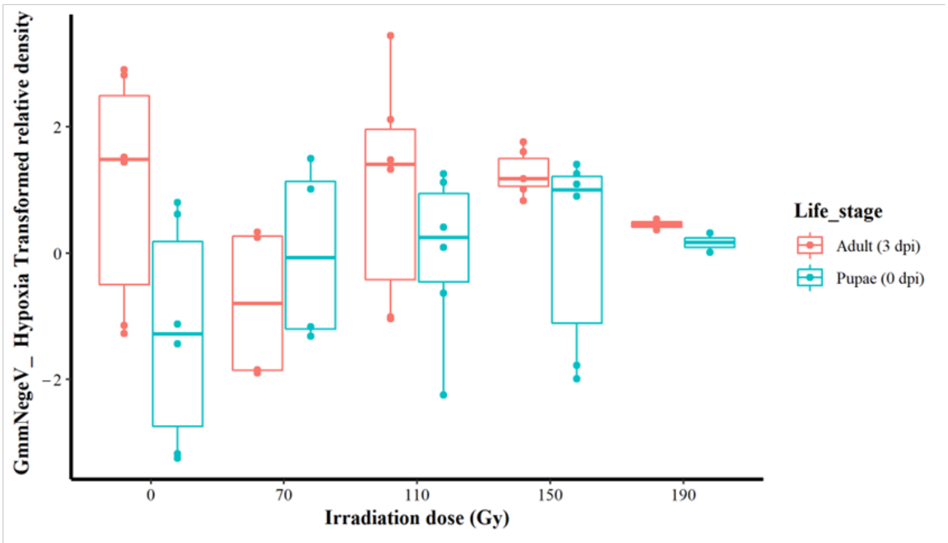

Supplementary Figure S3. Effect irradiation doses on the density of GmmNegeV in different life stage of *G. morsitans morsitans* following irradiation in different irradiation conditions. a): under normoxia, b}: Under hypoxia condition. Differences in the density of GmmNegeV as affected by irradiation doses, a comparison between the adult (red) and pupae (blue) stages.
